# Supplementary figures and images for: Early intervention with a glycerol throat spray containing cold-adapted cod trypsin after self-diagnosis of common cold: A randomised trial
Source: PLoS One. 2022 Jul 5;17(7):e0270699. doi: 10.1371/journal.pone.0270699 (PMC9255730; doi:10.1371/journal.pone.0270699)

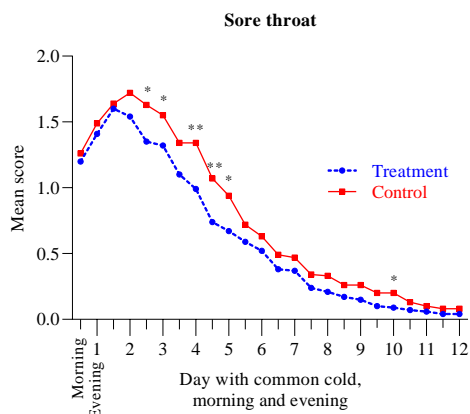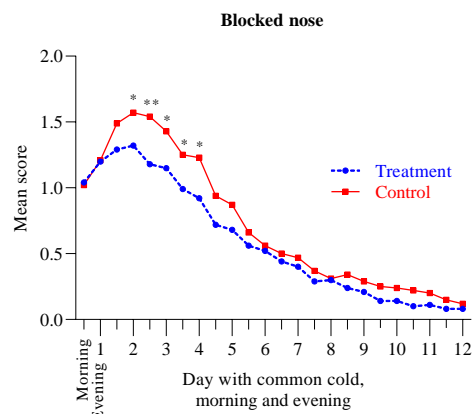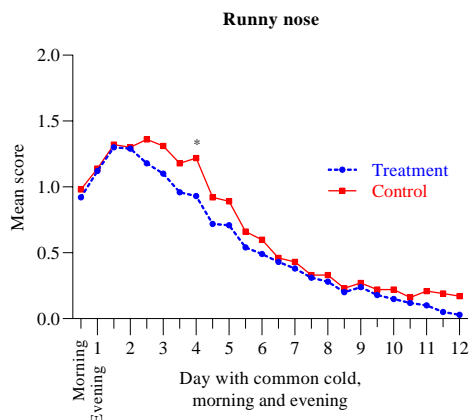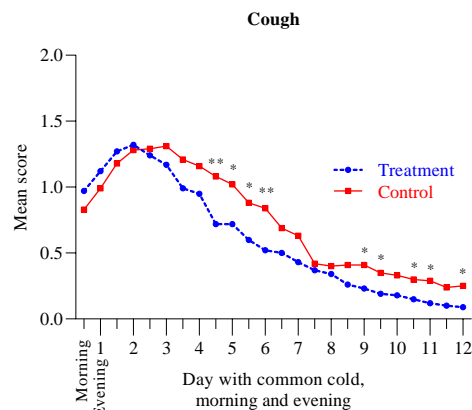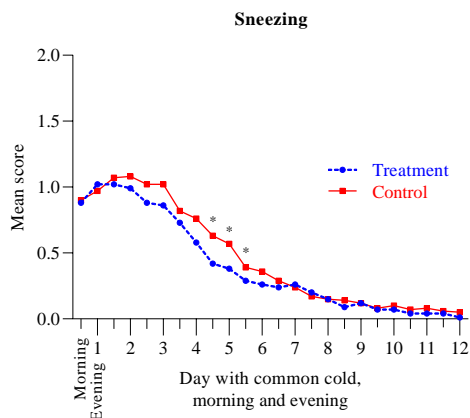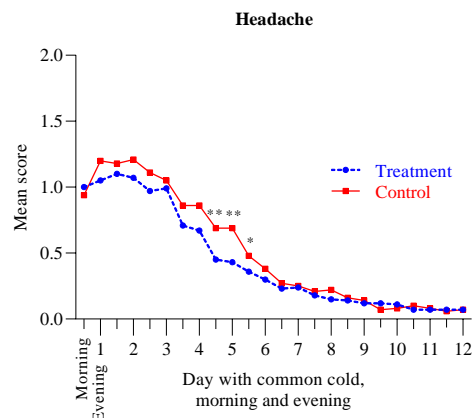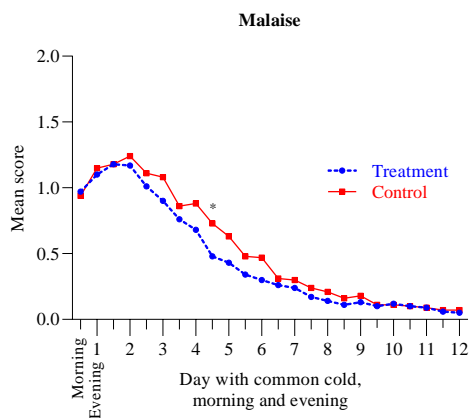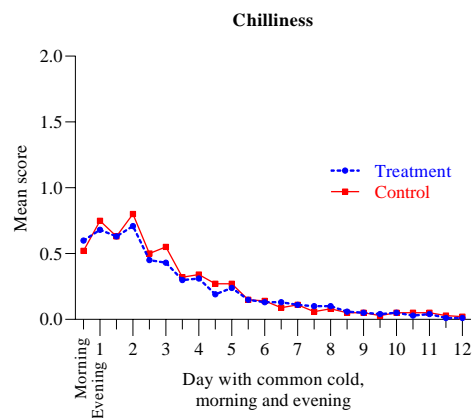

**Figure A: Jackson score individual items morning and evening day 1-12**

Supplement: S1 Fig — (PDF) [file pone.0270699.s002.pdf]

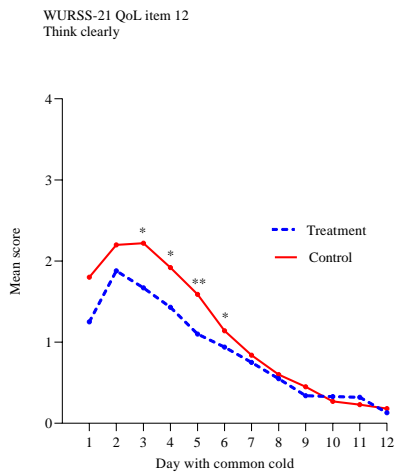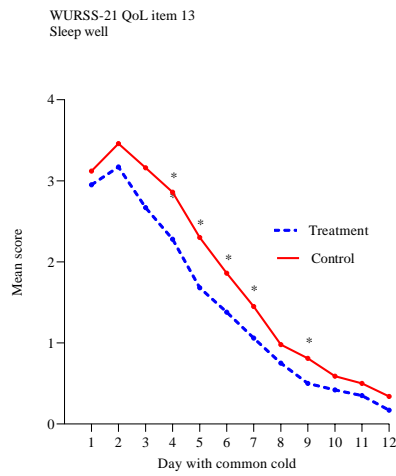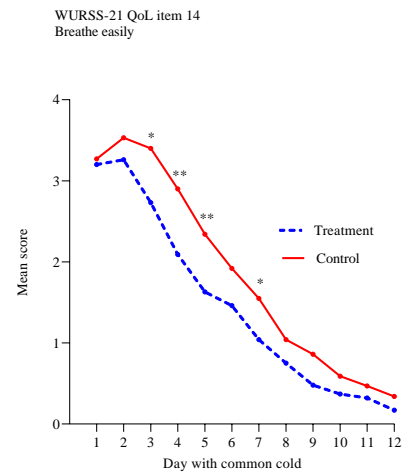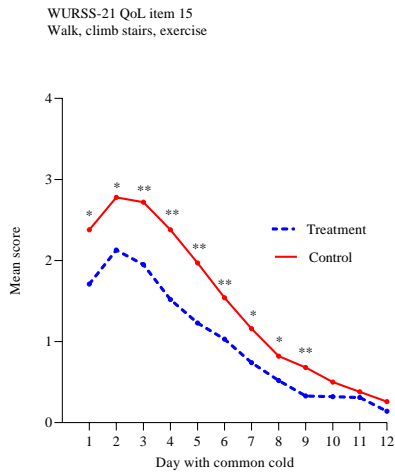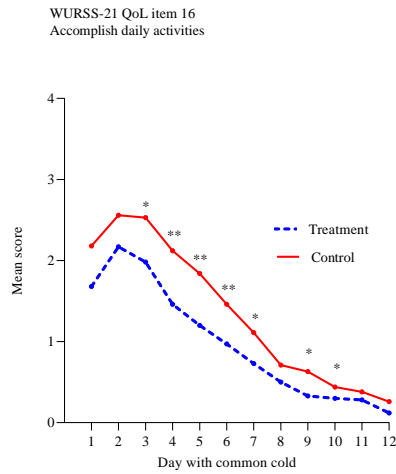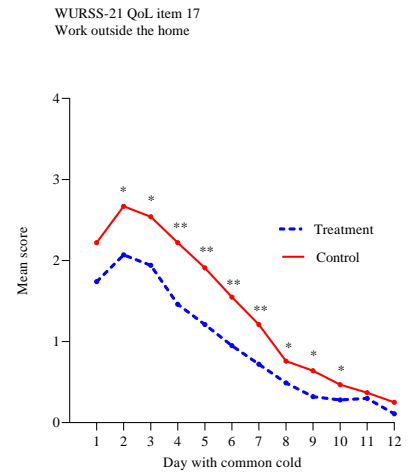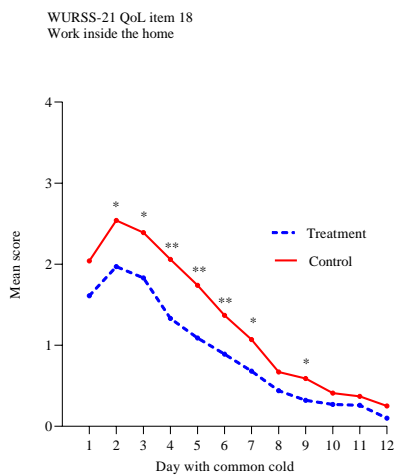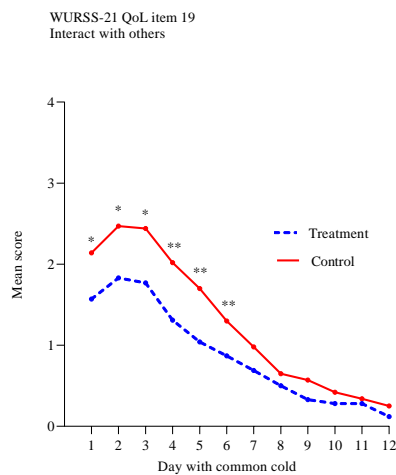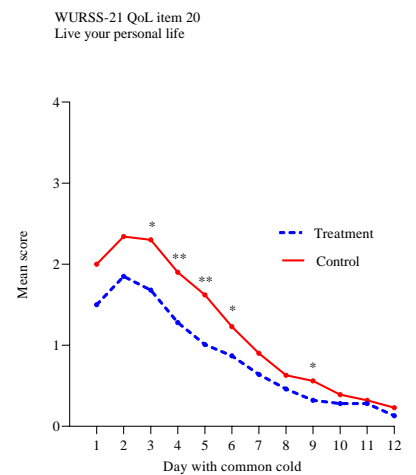

**Figure B: 9-item WURSS-21 QoL domain individual items: Daily mean score day 1-12**

Supplement: S2 Fig — (PDF) [file pone.0270699.s003.pdf]

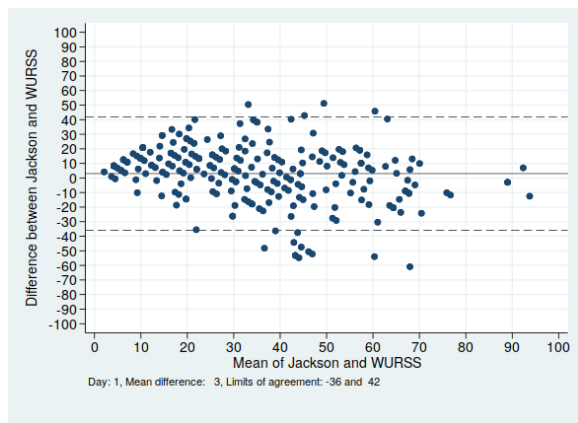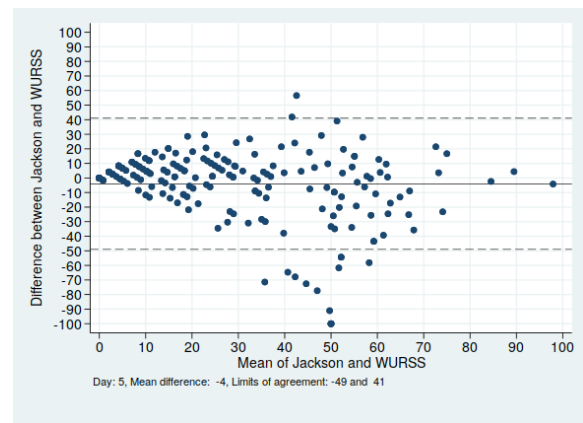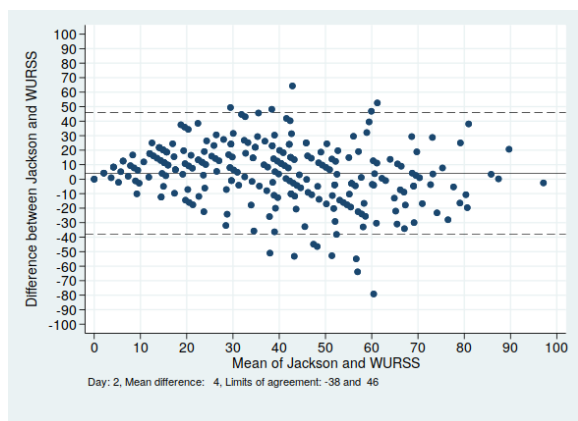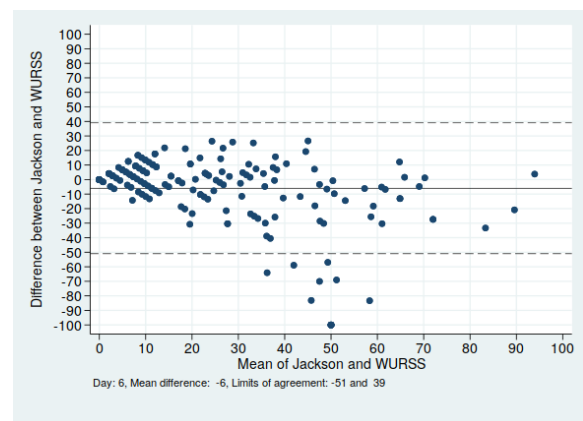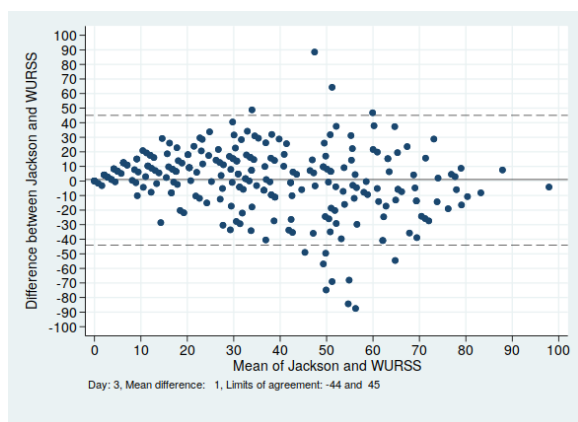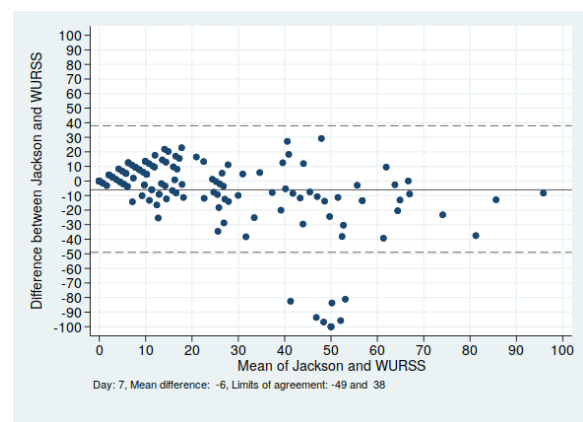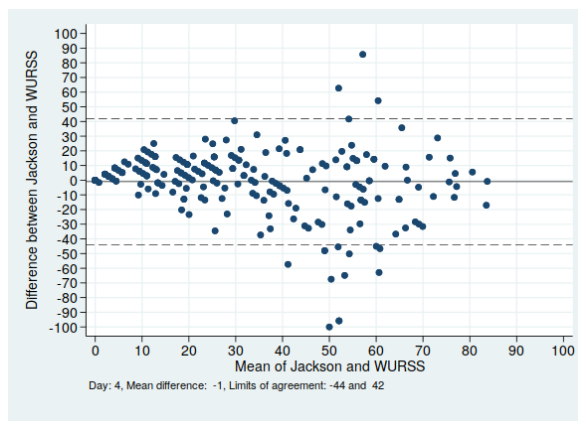

**Figure C: Comparison between Jackson score (evening) and 9-item WURSS-21 QoL domain day 1-7**

Supplement: S3 Fig — (PDF) [file pone.0270699.s004.pdf]
