# Supplementary material for: Early intervention with a glycerol throat spray containing cold-adapted cod trypsin after self-diagnosis of common cold: A randomised trial
Source: PLoS One. 2022 Jul 5;17(7):e0270699. doi: 10.1371/journal.pone.0270699 (PMC9255730; doi:10.1371/journal.pone.0270699)
Supplement: S1 File — (PDF) [file pone.0270699.s005.pdf]

**Clinical investigation plan**	
Investigational Study Product	CMS016317
Study Code	016317
Version	1.0
Date	15th November 2017

Single (investigator)-blind, randomized, parallel-group pilot study to evaluate the use of various assessments of common cold symptoms for proof of efficacy of CMS016317

Document versions

Version	Date	Author	Comments
1.0	15th Nov 2017	Gordana Bothe, PhD	

1 TABLE OF CONTENTS

1 TABLE OF CONTENTS 3
2 SYNOPSIS 6
3 LIST OF TERMS AND ABBREVIATIONS 11
4 INTRODUCTION 12
5 DESCRIPTION OF THE INVESTIGATIONAL PRODUCT 13
6 RISK ASSESSMENT FOR THE INVESTIGATIONAL PRODUCT AND THE CLINICAL INVESTIGATION 14
6.1 PROSPECTIVE CLINICAL BENEFIT 14
6.2 ANTICIPATED ADVERSE DEVICE EFFECTS 14
6.3 RISK ANALYSIS 14
6.3.1 Product Specific Risks 14
6.3.2 Risks due to Manufacturing Errors 14
6.3.3 Risks due to Overdose 15
6.3.4 Risks due to Improper Use 15
6.3.5 Risks due to Interactions with Pharmaceutical Products and/or Contraindications 15
6.3.6 Overall risk Assessment 15
6.4 RISKS ASSOCIATED WITH PARTICIPATION IN THE CLINICAL INVESTIGATION 15
6.5 RISK-TO-BENEFIT RATIO 15
7 OBJECTIVES OF THE CLINICAL INVESTIGATION 16
7.1 EFFICACY PARAMETERS 16
7.2 SAFETY PARAMETERS 16
7.3 FURTHER PARAMETERS 16
8 DESIGN AND DURATION OF THE CLINICAL INVESTIGATION 17
8.1 DESIGN OF THE CLINICAL INVESTIGATION 17
8.2 DURATION OF THE STUDY 17
9 SELECTION OF STUDY SUBJECTS 18
9.1 NUMBER OF SUBJECTS 18
9.2 INCLUSION CRITERIA 18
9.3 CRITERIA FOR CONTINUATION AT V2 18
9.4 EXCLUSION CRITERIA 18
9.5 WOMEN OF CHILD BEARING POTENTIAL 19
10 CLINICAL INVESTIGATION TREATMENT 20
10.1 THE INVESTIGATIONAL STUDY DEVICE 20
10.2 DOSAGE AND USE OF IP 20
10.3 PRODUCTION, PACKAGING AND LABELLING OF IP 21
10.4 STORAGE, INVENTORY, RETURN, TRACEABILITY AND DOCUMENTATION OF THE INVESTIGATIONAL PRODUCT 21
10.5 METHODS OF RANDOMISATION AND BLINDING 21
10.6 BLINDING 22
11 CONCURRENT TREATMENT 23
12 CONDUCT OF THE CLINICAL INVESTIGATION 24
12.1 VISIT 1 (V1, SCREENING, RANDOMISATION) 24
12.2 VISIT 2 (V2), 1– 3 DAYS AFTER SYMPTOM START 24
12.3 VISIT 3 (V3, FINAL), 16 +/-4 DAYS AFTER SYMPTOM START 24
12.4 TERMINATION VISIT (TV) FOR SUBJECTS WITH NO SYMPTOMS DURING THE STUDY PERIOD, 16 WEEKS +/-7 DAYS AFTER V1 25
12.5 DEMOGRAPHIC AND ANTHROPOMETRIC DATA 25

12.6 MEDICAL HISTORY / CLINICAL EXAMINATION 25
12.7 ASSESSMENT OF EFFICACY 25
12.7.1 Subject Diary / Common cold assessment at V2 25
12.7.2 Global Evaluation of the Efficacy by the Subjects 27
12.8 ASSESSMENT OF SAFETY 27
12.8.1 Blood Pressure and Pulse Rate 27
12.8.2 Adverse Events 27
12.8.3 Global Evaluation of Safety by the Subjects 27
12.9 ASSESSMENT OF FURTHER PARAMETERS 28
12.9.1 IP usage 28
12.10 STUDY DISCONTINUATION 28
12.10.1 Withdrawal of Subjects 28
12.10.2 Study Discontinuation Criteria 28

13 SAFETY EVALUATION AND REPORTING 30
13.1 DEFINITIONS 30
13.1.1 Adverse Event (AE) (ISO 14155) 30
13.1.2 Adverse Device Effect (ADE) (ISO 14155) 30
13.1.3 Device Deficiency (ISO 14155) 30
13.1.4 Serious Adverse Event (SAE) (ISO 14155) 30
13.1.5 Serious Adverse Device Effect (SADE) (ISO 14155) 30
13.1.6 Unanticipated Serious Adverse Device Effect (USADE) (ISO 14155) 31
13.1.7 Incidents (MPSV) 31
13.2 DOCUMENTING AND REPORTING ADVERSE EVENTS, ADVERSE DEVICE EFFECTS AND DEVICE DEFICIENCIES 31
13.2.1 Documenting Adverse Events and Adverse Device Effects by the Investigator 31
13.2.2 Documenting and Reporting Device Deficiencies and Incidents by the Investigator 31
13.2.3 Reporting Serious Adverse Events by the Investigator 32
13.2.4 Safety Evaluation and Reporting by the Sponsor 32

14 STATISTICS 33
14.1.1 Objective of the clinical investigation 33
14.1.2 Statistical hypothesis 33
14.1.3 Statistical methods 33
14.1.4 Drop-outs 33
14.1.5 Sample size estimation 34
14.1.6 Statistical analysis plan/report 34

15 ETHICAL AND LEGAL CONSIDERATIONS 35
15.1 REGULATORY ASPECTS 35
15.2 INDEPENDENT ETHICS COMMITTEE 35
15.3 SUBJECT INFORMATION AND INFORMED CONSENT FORM 35
15.4 INVESTIGATOR'S OBLIGATIONS 35
15.5 AMENDMENTS TO THE CIP 35
15.6 DATA PROTECTION AND CONFIDENTIALITY 36

16 QUALITY CONTROL AND QUALITY ASSURANCE 37
16.1 TRAINING 37
16.2 MONITORING 37
16.3 CIP DEVIATIONS 37
16.4 AUDITS AND INSPECTIONS 37

17 DATA HANDLING AND RECORD KEEPING 38
17.1 SOURCE DATA 38
17.2 DATA DOCUMENTATION 38

17.3 INVESTIGATOR SITE FILE 38
17.4 DATA MANAGEMENT 38
17.5 ARCHIVING 39
17.6 PRESENTATION OF DATA – FINAL REPORT 39

18 FINANCING / INSURANCE 40

19 PUBLICATION POLICY 41

20 LITERATURE 42

21 SIGNATURES 43

21.1 STUDY ADMINISTRATIVE STRUCTURE 44
21.2 STUDY FLOW CHART 45

2 SYNOPSIS

Title of the clinical investigation:	Single (investigator)-blind, randomized, parallel-group pilot study to evaluate the use of various assessments of common cold symptoms for proof of efficacy of CMS016317
Study code:	016317
Type of clinical investigation:	Single (investigator)-blind, randomized, parallel-group design, pilot study according to German Act on Medical Devices (MPG) §23b
Investigational product (IP):	CMS016317 is ColdZyme® Mouth Spray, a marketed Class I medical device; with the following composition: glycerol, water, cod trypsin, ethanol (<1 %), calcium chloride, trometamol and menthol.
Medical expert/ Principal investigator:	Prof. Ralf Uebelhack, MD<br>analyze & realize GmbH<br>Waldseeweg 6, 13467 Berlin, Germany<br>Phone: +49 30/40008 160<br>Fax: +49 30/40008 501<br>Email: ruebelhack@analyze-realize.com
Sponsor:	Johan Lindvall<br>Enzymatica AB<br>Ideon Science Park, 22370 Lund, Sweden<br>Phone: +46 46 286 31 00<br>Email: Johan.Lindvall@enzymatica.com
CRO:	analyze & realize GmbH<br>Waldseeweg 6<br>13467 Berlin, Germany<br><br>Project manager<br>Stephanie Seibt
Investigation site	analyze & realize GmbH<br>Weißenseerweg 111, 10369 Berlin, Germany
Clinical investigation objective:	To evaluate the use of various assessments of common cold symptoms for proof of efficacy of CMS016317
Proposed start of the study (first subject in):	Q1 2018
Proposed end of the study (last subject out):	Q1 2019
Visit schedule:	At screening visit (V1), eligible subjects will be randomised to two groups, the verum group and the “optional care only / no IP” group. All subjects will get the subject diary.<br><br>The subjects in the verum group have to use the IP as defined and may use optional care (see the definition in the “Duration of intervention” below) as needed in accordance with the definition of optional care.<br>The subjects in the “optional care only / no IP” group do not receive any IP and may use optional care as needed in accordance with the definition of optional care.<br><br>Within maximally 3 days after answering “yes” to the question on common cold and the onset of at least mild symptom(s) in Jackson scale (see “Duration of intervention”) (for both study groups) as well as the

	first IP use (for verum group), the subject should attend a visit on site (V2) and get an examination by the investigator.<br><br>Between V1 and V2, the study centre will have monthly phone contact with the subjects as a reminder about the study.<br><br>The termination visit (V3) will take place on day 16 (+/-4 days) after first IP use.<br><br>Subject with no symptoms during the study period will only have the termination visit (TV) at a time point of 16 weeks (+/-7 days) after V1.
Duration of intervention:	The IP use (verum group) should start when following conditions have been fulfilled:<br>• answering "Yes" to either of the questions in the subject diary: "Do you think/feel you have a cold?" or "Do you think/feel you are coming down with a cold (might be having first signs of cold)?"<br>• Jackson score of at least 1 (mild = sensible, but not disturbing or irritating) for any symptom except headache<br><br>The IP should be used until 2 days after the subject is symptom free (=answering "No" to the question "Do you think that you are still sick with this respiratory infection?" for 2 days in a row), but not longer than 12 days in total.<br><br>The following treatment is defined as optional care to be applied as needed during the common cold period:<br>• paracetamol (max. 2 g/day),<br>• ibuprofen (max. 400 mg/day),<br>• sea water nose drops or nose spray<br>• as well as antibiotics (in case they are necessarily required).
Duration of study:	Duration of study for each subject depends on the time point of symptom onset (see “Visit schedule”) and may be maximally 16 weeks.
Number of participants:	Expected number of subjects to be assessed for eligibility (at V1): n=300<br>Expected number of subjects to be allocated to trial/randomised: n=300<br>Expected number of subjects to enter the treatment phase (upon experiencing symptoms) and complete V2 and the study: n=200<br>Expected number of subjects to be analysed: n=200<br>Depending on the percentage of subjects from those randomized at V1 who actually delevop symptoms during the study period, the envisaged number of 300 recruited subjects may deviate from the final number of subjects needed.<br>For reasons of practicability/logistics of recruitment, the targeted number of 200 subjects with symptoms may be subject to deviations of maximally +/- 20 subjects.
Inclusion criteria:	1. Men and women<br>2. Age 18 to 70 years old<br>3. Increased risk for common cold (at least 3 self-reported occurencces of common cold within the last 12 months prior to V1) but generally in good health<br>4. Readiness to comply with trial procedures:<br>• Use of IP as recommended (verum group)<br>• Filling in diary

	• Keeping habitual diet and physical activity level<br>5. Women of child-bearing potential:<br>• Have to agree to use appropriate contraception methods<br>• Negative pregnancy testing (beta human chorionic gonadotropin test in urine) at V1<br>Participation is based upon written informed consent by the participant following written and oral information by the investigator regarding nature, purpose, consequences and possible risks of the clinical study.
Criteria for continuation at V2:	Subjects having Visit 2 within maximally 3 days after onset of common cold symptoms according to predefined criteria for required symptoms, and, for verum group, start of IP use, will continue study participation.
Exclusion criteria:	1. Known allergy or hypersensitivity to the components of the investigational product<br>2. History and/or presence of clinically significant condition/ disorder (self-reported), which per investigator's judgement could interfere with the results of the study or the safety of the subject, e.g.:<br>• Nasal disorder (e.g. polyposis, relevant septal deviation, ulcer etc.) and/or reconstructive surgery<br>• Acute/chronic airways disease/disorder (e.g. chronic obstructive lung disease, asthma, chronic cough of any origin)<br>• Acute psychiatric disorders<br>• Any other acute/chronic serious organ or systemic diseases<br>3. Influenza vaccination within the last 3 months prior to V1 and during the study<br>4. Regular use of products that may influence the study outcome (e.g. immune suppressants/immune stimulants including natural health products, analgesics/anti-rheumatics, anti-phlogistics, antitussives/expectorants, mouth or throat therapeutics, decongestants, antibiotics, anti-histaminergic drugs, nasal drops/spray) within the last 4 weeks prior to V1 and during the study (except for the defined optional care)<br>5. Pregnancy or nursing<br>6. History of (in the past 12 months prior to V1) or current abuse of drugs, alcohol or medication<br>7. Participation in the present study of a person living in the same household as the subject<br>8. Inability to comply with study requirements according to investigator’s judgement<br>9. Participation in another clinical study in the 30 days prior to V1 and during the study
Investigational product use:	The verum spray should be applied every second hour up to 6 times daily, each 2 puffs (1 dose).<br>6 times per day with each time 2 puffs per occasion.<br>Depending on the timepoint of treatment onset, there may be less IP applied on the first day of use.
Assessments:	1. Subject diary, questioning on:<br>a. Possible presence/absence of a common cold (twice daily, morning/evening)<br>b. Sore Throat Scale (twice daily, morning/evening)<br>c. Irritated Throat Scale (twice daily, morning/evening)<br>d. Jackson scale (twice daily, morning/evening)<br>e. WURSS-21 Quality of Life (QoL) (once daily, in the evening)<br>f. Use of the IP (verum group)<br>g. Use of any concomitant treatment / remedies

	2. Examination by investigator at V2 incl. assessment of common cold<br>3. Physical examination, vital signs (blood pressure/ pulse measurement) at all visits<br>4. Assessment of adverse events at all visits<br>5. Assessment of device deficiency at V2 and V3<br>6. Urinalysis at V1<br>7. Global evaluation of efficacy by subjects (4-point categorical scale) at V3<br>8. Global evaluation of safety and tolerability by subjects (4- point categorical scale) at V3
**Endpoints**	
Efficacy parameters	Main endpoints:<br>Evaluation of various assessments of common cold symptoms by the observed magnitude of treatment effects:<br>• Sore Throat Scale<br>• Irritated Throat Scale<br>• Jackson scale – total score, local symptoms, systemic symptoms, 2-item score (sore throat & malaise)<br>• WURSS-21 QoL subscore<br>• Percentage of subjects with prevention of cold outburst<br><br>Further endpoints<br>• Duration of common cold symptoms<br>• Use of concomitant treatment / remedies<br>• Global evaluation of efficacy by subjects at study end
Safety parameters	• Physical examination<br>• Blood pressure<br>• Pulse rate<br>• Global evaluation of safety and tolerability by subjects at study end<br>• Assessment of adverse events throughout the study<br>• Assessment of device deficiency at V2 and V3
Further parameters	Assessment of IP usage (based on IP account) in verum group
Biometry:	The present study is principally intended as an exploratory pilot study. Therefore, explorative data analyses shall be performed, including the calculation of known descriptive statistical quantities for the varied scaled variables under investigation. No prospectively specified statistical hypotheses will be proved, but possibly generated statistical hypotheses from the forecited descriptive analyses; the subsequent statistical tests are therefore only to be understood as exploratory ones, i.e. without any confirmative generalization of the results.<br>All end points as well as the concurrent and safety variables will receive an explorative examination and will be descriptively assessed. For the metric data (continuous data) the statistical characteristics will be given (number, mean, standard deviation, median, extremes, quartiles). For

	ordinal data (discrete data) number, median, interquartile range and extremes will be calculated. If found suitable, ordinal data will be considered as supplementary metric data. For all nominal data the frequency distribution will be presented in frequency tables. The values of metric data can be merged in ordinal classes according to clinical criteria to determine their frequency distribution. Explorative estimation for the structural consistency of the groups regarded will be proved by a descriptive comparison of demographic data, values at screening and secondary (further) endpoints.<br><br>At the current stage of knowledge, no reliable information about the underlying scientific object of research (assessments of common cold symptoms) is available. Therefore, no statistical planning of sample size is possible and the number of 200 subjects is only determined by practical needs of the investigator, taking account for the inter- and intra-individual variability and underlying physiological processes.
GCP conformity:	This clinical investigation will be performed based on the principles of the ICH GCP E6 (R2) and ISO 14155.

3 LIST OF TERMS AND ABBREVIATIONS

AE	Adverse Event
ADE	Adverse Device Effect
BMI	Body Mass Index
BfArM	Federal Institute for Drugs and Medical Devices (“Bundesinstitut für Arzneimittel und Medizinprodukte”)
CA	Competent Authority
CHMP	Committee for Medicinal Products for Human Use
CIP	Clinical Investigation Plan
CRA	Clinical Research Associate
CRF	Case Report Form
CRO	Contract Research Organization
EC	Ethics Committee
FAS	Full Analysis Set
FSFV	First subject first visit
GCP	Good Clinical Practice
GI	Gastrointestinal
ICH	International Conference on Harmonization
ICF	Informed Consent Form
IP	Investigational Product
ISF	Investigator Site File
ITT	Intention To Treat
LSO	Last Subject Out
MPG	German Act on Medical Devices (“Medizinproduktegesetz”)
MPSV	Ordinance on Medical Devices Vigilance (“Medizinprodukte-Sicherheitsplanverordnung”)
PP	Per Protocol
SAE	Serious Adverse Event
SADE	Serious Adverse Device Effect
SAP	Statistical Analysis Plan
SOP	Standard Operating Procedure
TMF	Trial Master File
USADE	Unexpected Serious Adverse Device Effect
V	Visit
VCAS	Valid Case Analysis Set
WHO	World Health Organization

All official and professional titles and any references to persons in this document apply to both genders.

4 INTRODUCTION

A common cold is a trivial catarrhal infection of the upper respiratory tract (Tyrrell 1996). Acute respiratory diseases are among the most frequent illnesses at all. They are almost exclusively based on viral infections and mostly show an epidemic progression affecting all age groups, with the well-known autumn/winter peak (Glathe 1992, Wagner 1996).

The typical progression may differ in individual subjects, but in any case it presents a burden for the subject. Most commonly, adult subjects complain of a general feeling of sickness, headache, pain in the limbs, rhinorrhea (runny nose), sore throat and difficulty swallowing, hoarseness, coughing and altered quality of sleep.

An adequate and practice relevant, primary causal (e.g. antiviral) therapy against a trivial acute infection of the upper respiratory tract is currently not available. However, many of the complaints can be reduced by treating the symptoms (Ziegler 1995).

The development of effective treatment options is difficult due to the multiple virus types and the ways of interaction between virus and host (Johnston, 1997; Hendley, 2008). Numerous remedies are available on prescription and over the counter, yet there is still no reliable treatment and the potential side effects may be substantial (Eccles et al., 2010). Effective solutions with minimal side effects (given the nonhazardous nature of the disorder) are required.

CMS016317 is ColdZyme® Mouth Spray (Enzymatica AB, Sweden), a marketed Class I medical device, with the following composition: glycerol, water, cod trypsin, ethanol (<1 %), calcium chloride, trometamol and menthol. It is to be used when exposed to cold viruses or early on when cold symptoms occur. It works by coating the lining of the mucous membrane, creating a protective barrier. The barrier acts osmotically on the cold viruses, trapping them and preventing them from binding with human cells, helping the body to remove them naturally.

The present exploratory study is designed to evaluate the use of various assessments of common cold symptoms for proof of efficacy of CMS016317.

5 DESCRIPTION OF THE INVESTIGATIONAL PRODUCT

CMS016317 is ColdZyme® Mouth Spray, a medical device classified as a Class I device (CE-marked and marketed since 2013) under Council Directive 93/42/EEC on Medical Device and MED-DEV Guidance Document 2.4/1. Rev 8, June 2010, with the following composition: glycerol, water, cod trypsin, ethanol (<1 %), calcium chloride, trometamol and menthol. The manufacturer of ColdZyme® Mouth Spray is Enzymatica AB, the study sponsor.
ColdZyme® Mouth Spray is to be used when exposed to cold viruses or early on when cold symptoms occur. It targets the mouth and throat, where cold viruses take hold and multiply.

The intended use of ColdZyme® Mouth Spray (according to the Instruction for Use, 2016):

- It reduces the probability of catching a cold
- it can help shorten the duration of a cold if used at an early stage of the infection.

In a recent non-clinical study (Stefansson et al, 2017), it was demonstrated that ColdZyme® Mouth Spray has a virus deactivating ability against four major common cold viruses. A virucidal efficacy suspension test was conducted using ColdZyme® Mouth Spray against each of the challenge viruses in suspension. ColdZyme® Mouth Spray deactivated rhinovirus type 1A by 91.7% (1.08 log10), rhinovirus type 42 by 92.8% (1.14 log10), human influenza A virus H3N2 by 96.9% (1.51 log10), respiratory syncytial virus (RSV) by 99.9% (2.94 log10) and adenovirus type 2 by 64.5% (0.45 log10).

Further, a recent clinical study (Clarsund et al., 2017) investigated the performance of ColdZyme® Mouth Spray in rhinovirus-inoculated healthy volunteers. This randomized, doubleblind, placebo-controlled pilot study was conducted on 46 healthy volunteers inoculated with rhinovirus 16 via the nose. Subjects self-administered ColdZyme or placebo 6 times daily for 11 days. Symptoms were recorded daily in a diary. Rhinovirus 16 in nasal and oropharyngeal samples at days 0, 3, 4, 6, 7 and 10 were quantified by RT-qPCR. The primary outcome measure was the reduction in viral load in oropharyngeal samples. Exploratory analysis measuring the total viral load (i.e., area under the curve) for days 3 - 10 in successfully inoculated subjects found that ColdZyme® Mouth Spray treatment resulted in a lower total viral load in the oropharynx ($p = 0.023$). In subjects who experienced symptomatic common cold, treatment with ColdZyme® Mouth Spray resulted in a reduction in the number of days with common cold symptoms from 6.5 to 3.0 days ($p = 0.014$) in comparison to placebo.

6 RISK ASSESSMENT FOR THE INVESTIGATIONAL PRODUCT AND THE CLINICAL INVESTIGATION

6.1 PROSPECTIVE CLINICAL BENEFIT

Based on the available clinical evidence, an improvement of the common cold symptoms is anticipated within the period of treatment with CMS016317 (ColdZyme® Mouth Spray).

6.2 ANTICIPATED ADVERSE DEVICE EFFECTS

The essential requirements relevant for the device safety and performance have been assessed within an extensive risk management process, which incorporates biological evaluation, design, usability, production, quality and regulatory processes and clinical validation. Evaluation of the analysis combining all potential risks has concluded that the ColdZyme® Mouth Spray is free from unacceptable risk. No contraindications for use of the device, other than hypersensitivity to any of the ingredients, have been identified in the risk management process. Risks have been further addressed in post market surveillance and in post market investigations. No new or unacceptable risks have been recorded, and no adverse events related to the use of the ColdZyme® Mouth Spray have been reported during post market investigations. Trypsin is also Generally Regarded As Safe (GRAS) in humans by the US FDA (21 CFR 184, §184.1914).

Therefore, the device is concluded to achieve its intended purpose and fulfil essential requirements regarding safety and performance.

ColdZyme® Mouth Spray should not be used by individuals hypersensitive/allergic to any of the ingredients. Further, it should not be used continuously for more than 30 days, as there is no clinical information available about long-term use.

It is most important not to inhale when applying the spray since this may cause transient asthma-like symptoms, such as coughing and hoarseness; the study subjects will be instructed accordingly.

6.3 RISK ANALYSIS

6.3.1 Product Specific Risks

Safety of ColdZyme® Mouth Spray has been assessed within the risk management process and evaluation of the analysis combining all potential risks has concluded that ColdZyme® Mouth Spray does not impose any unacceptable risk. The essential requirements relevant for ColdZyme® Mouth Spray safety have been handled according to the Risk Management process. Risks have been identified, evaluated according to the essential requirements with applicable harmonized standards, mitigated and, where applicable, controlled via verified control measures.

6.3.2 Risks due to Manufacturing Errors

To mitigate the risk, the production is monitored by means of a quality management system ensuring that the quality of the manufactured product complies with the specified standards.

6.3.3 Risks due to Overdose

To mitigate the risk, the dosing recommendations for the intended use of the device are derived from the clinical evaluation of the product.

6.3.4 Risks due to Improper Use

There is labeling on the device packaging to state proper use and general and specific precautions including 'use by date' statement and proper storage requirements. In the study, the subjects will be trained on proper use (please refer to section 10.2).

6.3.5 Risks due to Interactions with Pharmaceutical Products and/or Contraindications

According to the Instruction for Use (2016), ColdZyme® Mouth Spray should not be used by individuals hypersensitive/allergic to any of the ingredients.

6.3.6 Overall risk Assessment

According to the Risk Management Report, possible risks are acceptable due to low probability of occurrence and existence of sufficient countermeasures.

6.4 RISKS ASSOCIATED WITH PARTICIPATION IN THE CLINICAL INVESTIGATION

All clinical assessments are standard in health care applied for upper respiratory tract infections.

6.5 RISK-TO-BENEFIT RATIO

The current overall risk assessment reveals that, when used as intended, CMS016317 (ColdZyme® Mouth Spray) does not pose an unjustifiable risk. Given the fact that the evidence available suggests beneficial effects with respect to the common cold symptoms, the risk-to-benefit-ratio in the present investigation may be considered as favourable.

7 OBJECTIVES OF THE CLINICAL INVESTIGATION

The main goal of this single (investigator)-blind, randomized, parallel-group design, pilot clinical investigation is to evaluate the use of various assessments of common cold symptoms for proof of efficacy of CMS016317.

7.1 EFFICACY PARAMETERS

Main endpoints:

Evaluation of various assessments of common cold symptoms by the observed magnitude of treatment effects:

- Sore Throat Scale
- Irritated Throat Scale
- Jackson scale – total score, local symptoms, systemic symptoms, 2-item score (sore throat & malaise)
- WURSS-21 QoL subscore
- Percentage of subjects with prevention of cold outburst

Further endpoints

- Duration of common cold symptoms
- Use of concomitant treatment / remedies
- Global evaluation of efficacy by subjects at study end

7.2 SAFETY PARAMETERS

- Physical examination
- Blood pressure
- Pulse rate
- Global evaluation of safety and tolerability by subjects at study end
- Assessment of adverse events throughout the study
- Assessment of device deficiency at V2 and V3

7.3 FURTHER PARAMETERS

- Assessment of IP usage (based on IP account) in verum group

8 DESIGN AND DURATION OF THE CLINICAL INVESTIGATION

8.1 DESIGN OF THE CLINICAL INVESTIGATION

The present clinical investigation is a single (investigator)-blind, randomized, parallel-group design, pilot study.

Screening/ enrolment	Treatment start	Control visit	Treatment stop
All	Onset of symptoms		
Visit 1		2	3
		Within 3 days from start of symptoms	16+/-4 days from start of symptoms

Figure 1: Visit schedule

8.2 DURATION OF THE STUDY

The study duration for each subject is maximally 16 weeks.
The expected start date of this study is Q1 2018. The clinical phase of the study is expected to be completed by Q1 2019.
If the required number of subjects with cold symptoms would not be achieved in the winter season 2017/2018, the recruitment would be stopped between March and September 2018 and continued in the next winter season (2018/2019).

9 SELECTION OF STUDY SUBJECTS

9.1 NUMBER OF SUBJECTS

A total number of expectedly 300 subjects will be enrolled in the study as eligible study participants and randomised at V1. Thereof, the first 200 who attend the V2 should complete the study (i.e. enter the treatment phase upon experiencing symptoms).
Depending on the percentage of subjects from those randomized at V1 who actually delevop symptoms during the study period, the envisaged number of 300 recruited subjects may deviate from the final number of subjects needed.
For reasons of practicability/logistics of recruitment, the targeted number of 200 subjects with symptoms may be subject to deviations of maximally +/- 20 subjects.

9.2 INCLUSION CRITERIA

1. Men and women
2. Age 18 to 70 years old
3. Increased risk for common cold (at least 3 self-reported occurencces of common cold within the last 12 months prior to V1) but generally in good health
4. Readiness to comply with trial procedures:
   - Use of IP as recommended (verum group)
   - Filling in diary
   - Keeping habitual diet and physical activity level
5. Women of child-bearing potential:
   - Have to agree to use appropriate contraception methods
   - Negative pregnancy testing (beta human chorionic gonadotropin test in urine) at V1

Participation is based upon written informed consent by the participant following written and oral information by the investigator regarding nature, purpose, consequences and possible risks of the clinical study.

9.3 CRITERIA FOR CONTINUATION AT V2

Subjects having Visit 2 within maximally 3 days after onset of common cold symptoms according to predefined criteria for required symptoms, and, for verum group, start of IP use, will continue study participation.

9.4 EXCLUSION CRITERIA

1. Known allergy or hypersensitivity to the components of the investigational product
2. History and/or presence of clinically significant condition/ disorder (self-reported), which per investigator's judgement could interfere with the results of the study or the safety of the subject, e.g.:
   - Nasal disorder (e.g. polyposis, relevant septal deviation, ulcer etc.) and/or reconstructive surgery
   - Acute/chronic airways disease/disorder (e.g. chronic obstructive lung disease, asthma, chronic cough of any origin)
   - Acute psychiatric disorders
   - Any other acute/chronic serious organ or systemic diseases
3. Influenza vaccination within the last 3 months prior to V1 and during the study
4. Regular use of products that may influence the study outcome (e.g. immune suppressants/immune stimulants including natural health products, analgesics/anti-

rheumatics, anti-phlogistics, antitussives/expectorants, mouth or throat therapeutics, decongestants, antibiotics, anti-histaminergic drugs, nasal drops/spray) within the last 4 weeks prior to V1 and during the study (except for the defined optional care)

5. Pregnancy or nursing
6. History of (in the past 12 months prior to V1) or current abuse of drugs, alcohol or medication
7. Participation in the present study of a person living in the same household as the subject
8. Inability to comply with study requirements according to investigator's judgement
9. Participation in another clinical study in the 30 days prior to V1 and during the study

9.5 WOMEN OF CHILD BEARING POTENTIAL

Female subjects may not be pregnant at the time of inclusion into the study. Therefore, pregnancy testing will be performed during screening for women with child bearing potential (women of non-childbearing potential are defined as those who either have no uterus, or have undergone ligation of the fallopian tubes, or have permanent cessation of ovarian function due to ovarian failure or surgical removal of the ovaries, or are in the postmenopause as per investigator's judgement). During the informed consent process, the investigator will advise every woman with child bearing potential that she must use medically recognised contraception during the study duration. In case pregnancy occurs, the subject has to withdraw. Any pregnancy during the study must be notified in writing to the sponsor and the project manager of the CRO by the investigator within 24 hours after their awareness of the event by sending the respective form to:

Fredrik Lindberg, MD, PhD;

Phone: +46 46 286 31 00, email: Fredrik.Lindberg@enzymatica.com

Stephanie Seibt;

Phone: +49 30 40008 8 144, Fax: +49 30 40008 501

Initial report must be followed up by a detailed written report.

10 CLINICAL INVESTIGATION TREATMENT

10.1 THE INVESTIGATIONAL STUDY DEVICE

CMS016317 is ColdZyme® Mouth Spray, a Class I medical device (CE-marked) under Council Directive 93/42/EEC on Medical Device and MED-DEV Guidance Document 2.4/1. Rev 8, June 2010; with the following composition: glycerol, water, cod trypsin, ethanol (<1 %), calcium chloride, trometamol and menthol.

ColdZyme® Mouth Spray consists of a 20 ml bottle, pump, spray nozzle and protective cap. A thin plastic label is added to the bottle.

10.2 DOSAGE AND USE OF IP

The spray should be applied every second hour up to 6 times with each time 2 puffs (1 dose) per occasion. At V1, the subject will be instructed on the use and the application demonstrated.

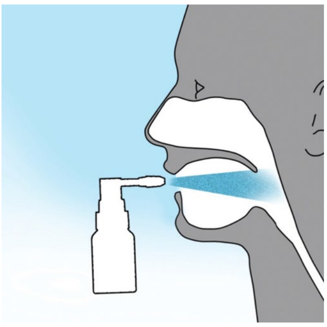

Figure 2: Application of spray

Depending on the timepoint of treatment onset, there may be less IP applied on the first day of use.

The IP use should start when following conditions have been fulfilled:

- answering "Yes" to either of the questions in the subject diary: "Do you think/feel you have a cold?" or "Do you think/feel you are coming down with a cold (might be having first signs of cold)?"
- Jackson score of at least 1 (mild = sensible, but not disturbing or irritating) for any symptom except headache

The IP should be used until 2 days after the subject is symptom free (=answering "No" to the question "Do you think that you are still sick with this respiratory infection?" for 2 days in a row), but not longer than 12 days in total.

10.3 PRODUCTION, PACKAGING AND LABELLING OF IP

Production and packaging of the IP will be taken over by the sponsor, Enzymatica AB. Labelling will be provided by analyze & realize GmbH.
The labels will contain the following information (in German):

- Random code
- Sponsor: Enzymatica AB, Ideon Science Park, 22370 Lund, Sweden
- CRO: analyze & realize GmbH, Waldseeweg 6, 13467 Berlin, phone: +49 30/40008100
- Name of the investigational product: CMS016317
- Batch number:
- Dosage: every second hour up to 6 times with each time 2 puffs (1 dose)
- Content per package:
- Expiry date:
- Study code: 016317
- “For clinical study use only”
- Store at room temperature up to maximally 25 °C
- Keep out of reach of children

10.4 STORAGE, INVENTORY, RETURN, TRACEABILITY AND DOCUMENTATION OF THE INVESTIGATIONAL PRODUCT

The investigational product will be provided to the CRO by the sponsor. The CRO will be responsible for storing the investigational product in a proper place, inaccessible to unauthorized persons.

The CRO will distribute the investigational product to the investigator who will hand it out to the subjects. The investigators will be responsible for storing the IP at the appropriate conditions and inaccessible to unauthorized persons. The investigators will only dispense IP to subjects enrolled in this clinical investigation.

The IP should be stored at room temperature up to maximally 25 °C. Exposure to higher temperature (max 37˚C), e.g. to carry the product in a pocket is tolerated for a maximum of 24 accumulated hours throughout the study period. Subjects are not allowed to carry the IP in a pocket of a clothing piece that may be expected to get a temperature close to the body temperature (e.g. trousers, skirt).

Used IP will be collected from the subjects and the IP accountability (per vial weight) will be performed. After completion of the investigation, the empty and unused portion of the IP (if any) will be returned to the CRO and the unused IP either returned to the sponsor or destroyed (as per agreement with the sponsor).

10.5 METHODS OF RANDOMISATION AND BLINDING

The clinical investigation will be conducted in a single-blind randomised manner. The subjects will be randomised at the screening visit. The randomisation list will be provided to the sponsor’s assigned responsible person (not involved in the study) by the statistician responsible for generation of the randomisation list. The ratio of randomisation between the “IP group” (IP, and optional care as needed) and the “peer-group” (optional care only, no IP) will be 1:1. Random numbers will be assigned to the subjects in a sequential order based on time of random-

isation at each investigational site (several whole blocks will be allocated to each centre). Randomisation list will be concealed to the investigational sites. It will be stored under lock and key by the sponsor until database closure. After database closure and sign-off of the statistical analysis plan, the sponsor's responsible person will provide the randomisation list to the project manager at the CRO and the statistician responsible for the statistical analysis. The study allows for stratification with respect to centres (sites).

10.6 BLINDING

In this single-blind study, the subjects, the study site staff handling the IP and the study monitors will not be blinded to treatment assignment. The investigator performing the cold symptom assessment at V2, the project manager, the statistician and all further study team members will be blinded to treatment assignment.

The unblinded site study staff and the monitor(s) will take utmost care not to unblind the investigator performing the cold symptom assessment at V2 or any further blinded study team members at any occasion during the entire study duration.

The subjects will be instructed not to unblind the investigator.

11 CONCURRENT TREATMENT

The subjects report the concurrent treatment (e.g. other medical devices, medication, any natural health products including food supplements etc.) to the investigator at the first visit. The investigator documents the following information in the CRFs: substance (only for treatment), dosage (only for treatment), start and duration, reason. Subjects will also be advised to inform the investigators of any treatment performed throughout the clinical investigation period.

During the study, the use of any concurrent treatment according to exclusion criteria is not allowed:

- Influenza vaccination within the last 3 months
- Regular use of products that may influence the study outcome (e.g. immune suppressants/immune stimulants including natural health products, analgesics/anti-rheumatics, anti-phlogistics, antitussives/expectorants, mouth or throat therapeutics, decongestants, antibiotics, antihistaminergic drugs, nasal drops/spray) (except for the defined optional care)

At enrollment, the investigator informs the subjects that any concomitant treatment that could possibly influence the outcome of the study is not allowed, except for the defined optional care.

The following treatment is defined as optional care to be applied as needed during the common cold period:

- paracetamol (max. 2 g/day)
- ibuprofen (max. 400 mg/day)
- sea water nose drops or nose spray
- antibiotics (in case they are necessarily required).

12 CONDUCT OF THE CLINICAL INVESTIGATION

12.1 VISIT 1 (V1, SCREENING, RANDOMISATION)

- Oral and written information about the nature, purpose, possible risks and benefits of the study provided to the subjects by the investigator
- Written consent of the subject to participate; the subject understands the requirements of the clinical investigation and is willing to comply
- Questioning and documentation of the medical history, concurrent diseases and treatment, demographic and anthropometric data, and physical examination by the investigator
- Verification that the inclusion criteria are met and that there are no violations of the exclusion criteria
- Measurement of blood pressure and pulse rate
- Urinalysis and pregnancy test (for women with childbearing potential)
- Issue of subject diary and instruction for recording
- Randomisation
- Issue of IP (verum group) and instruction for use
- Questioning and documentation of possible occurrence of adverse events (AEs)

12.2 VISIT 2 (V2), 1– 3 DAYS AFTER SYMPTOM START

- Questioning and documentation of possible occurrence of AEs/device deficiencies
- Questioning and documentation of new or changed concurrent treatment
- Review of the diary by a study team member (except investigator)
- Assessment and documentation of cold
- Checking criteria for further participation
- Physical examination
- Measurement of blood pressure and pulse rate

Between V1 and V2, the study site will have monthly phone contact with the subjects as a reminder about the study.

12.3 VISIT 3 (V3, FINAL), 16 +/-4 DAYS AFTER SYMPTOM START

- Questioning and documentation of possible occurrence of AEs/device deficiencies
- Questioning and documentation of new or changed concurrent treatment
- Return and control of the subject diary by a study team member (except investigator)
- Return of IP (verum group) and accountability
- Physical examination
- Measurement of blood pressure and pulse rate

- Global evaluation of efficacy by subjects (4-point categorical scale)
- Global evaluation of safety and tolerability by subjects (4-point categorical scale)

12.4 TERMINATION VISIT (TV) FOR SUBJECTS WITH NO SYMPTOMS DURING THE STUDY PERIOD, 16 WEEKS +/-7 DAYS AFTER V1

- Questioning and documentation of possible occurrence of AEs
- Questioning and documentation of new or changed concurrent treatment
- Return of the subject diary
- Return of IP (verum group)

12.5 DEMOGRAPHIC AND ANTHROPOMETRIC DATA

The subject's age (years), gender (male/female) and ethnicity will be assessed and documented in the CRF at V1.

12.6 MEDICAL HISTORY / CLINICAL EXAMINATION

At V1, the subject will be questioned by the investigator regarding the medical history and will undergo a clinical examination. Known pre- or co-morbidities as well as any abnormal and/or pathological findings of the examination will be systematically recorded.

A dipstick urinalysis for the assessment of glucose and proteins and a pregnancy test in women with childbearing potential will be performed.

12.7 ASSESSMENT OF EFFICACY

12.7.1 Subject Diary / Common cold assessment at V2

In the daily subject diary, from V1 on, subjects will be asked to answer the questions "Do you think/feel you have a cold?" or "Do you think/feel you are coming down with a cold (might be having first signs of cold)?" (in the morning and in the evening).

From the first day they answer „Yes“ to either of the above questions, they will have to start filling out the cold diary instead, to:

- record their symptoms on the Jackson scale (twice daily, in the morning and in the evening)
- record their symptoms on the Sore Throat Scale (twice daily, in the morning and in the evening)
- record their symptoms on the Irritated Throat Scale (twice daily, in the morning and in the evening)
- fill out the Wisconsin Upper Respiratory Symtom Survey (WURSS-21) Quality of Life section (once daily, in the evening)
- answer the question "Do you think that you are still sick with this respiratory infection?" (once daily, in the evening)
- record the use of IP (verum group only; once daily, in the evening)
- record the use of any concomitant treatment / remedies (once daily, in the evening).

The cold diary should be filled out until 2 days after the subject is symptom free (=answering "No" to the question "Do you think that you are still sick with this respiratory infection?" for 2 days in a row).

The Jackson score (Jackson, 1958) is calculated by summing the following 8 symptom scores: sore throat, blocked nose, runny nose, cough and sneezing (local symptoms) as well as head-ache, malaise, and chilliness (systemic symptoms). Symptoms are assessed on a 4-point scale: 0 = none (symptom not present), 1 = mild (sensible, but not disturbing or irritating), 2 = moderate (symptoms sometimes disturbing/irritating), 3 = severe (symptoms disturbing/irritating most of the time).

The Sore Throat is a 0-10 Likert scale where 0=not sore and 10=very sore.

Irritated Throat Scale is a 0-10 Likert scale where 0=not irritated and 10=very irritated.

WURSS-21 (Barrett et al., 2009) is an evaluative illness-specific quality of life instrument with 21 items, designed to assess the negative impact of acute upper respiratory infection, presumed viral (the common cold). In this study, the QoL part of the WURSS-21 will be applied, from item 12 ("think clearly") to item 20 ("live your personal life").

Wisconsin Upper Respiratory Symptom Survey – 21 --- *Daily Symptom Report*

Day:	*Date:*	*Time:*	*ID:*

Please fill in one circle for each of the following items:

	Not sick	Very mildly		Mildly		Moderately		Severely
	0	1	2	3	4	5	6	7
How sick do you feel **today**?	○	○	○	○	○	○	○	○

Please rate the **average severity** of your cold symptoms over the **last 24 hours** for each symptom:

	Do not have this symptom	Very mild		Mild		Moderate		Severe
	0	1	2	3	4	5	6	7
Runny nose	○	○	○	○	○	○	○	○
Plugged nose	○	○	○	○	○	○	○	○
Sneezing	○	○	○	○	○	○	○	○
Sore throat	○	○	○	○	○	○	○	○
Scratchy throat	○	○	○	○	○	○	○	○
Cough	○	○	○	○	○	○	○	○
Hoarseness	○	○	○	○	○	○	○	○
Head congestion	○	○	○	○	○	○	○	○
Chest congestion	○	○	○	○	○	○	○	○
Feeling tired	○	○	○	○	○	○	○	○

Over the last 24 hours, how much has your cold interfered with your ability to:

	Not at all	Very mildly		Mildly		Moderately		Severely
	0	1	2	3	4	5	6	7
Think clearly	○	○	○	○	○	○	○	○
Sleep well	○	○	○	○	○	○	○	○
Breathe easily	○	○	○	○	○	○	○	○
Walk, climb stairs, exercise	○	○	○	○	○	○	○	○
Accomplish daily activities	○	○	○	○	○	○	○	○
Work outside the home	○	○	○	○	○	○	○	○
Work inside the home	○	○	○	○	○	○	○	○
Interact with others	○	○	○	○	○	○	○	○
Live your personal life	○	○	○	○	○	○	○	○

Compared to yesterday, I feel that my cold is…

Very much better	Somewhat better	A little better	The same	A little worse	Somewhat worse	Very much worse
○	○	○	○	○	○	○

WURSS -21© (Wisconsin Upper Respiratory Symptom Survey) 2004
Created by Bruce Barrett MD PhD et al., UW Department of Family Medicine, 777 S. Mills St. Madison, WI 53715, USA

The IP use should start when following conditions have been fulfilled:

- answering "Yes" to either of the questions in the subject diary: "Do you think/feel you have a cold?" or "Do you think/feel you are coming down with a cold (might be having first signs of cold)?"
- Jackson score of at least 1 (mild = sensible, but not disturbing or irritating) for any symptom except headache

Within maximally 3 days after start of the symptoms, the subject should attend the Visit 2 at the investigational site. If the limitation of 3 days may not be held due to e.g. weekend and/or holiday, the Visit 2 could take place on the day following the last non-working day but needs to be clearly documented in the CRF together with an explanation to the delayed visit. During Visit 2, the investigator will perform a physical examination and the study team (not the investigator) will check the subject diaries. The investigator will examine whether the symptoms reported by the subject are still present and if so, whether they are attributable to common cold or the subject suffers from another ailment (e.g. allergy, influenza, sinusitis, pharyngitis, tonsillitis, laryngitis, reflux disease, infectious mononucleosis etc.).

The IP should be used until 2 days after the subject is symptom free (=answering "No" to the question "Do you think that you are still sick with this respiratory infection?" for 2 days in a row), but not longer than 12 days in total.

A common cold episode is defined by the following criteria, which must be met by the subjects at least two days in a row:

1) answering "Yes" to the question "Do you think that you are still sick with this respiratory infection?" and
2) reporting at least 1 of 4 cold symptoms: nasal discharge (runny nose); nasal obstruction (plugged or congested); sneezing; or sore (scratchy) throat, and
3) scoring at least 2 points on the Jackson scale.

Based on the assessment of the symptoms recorded in the diary by means of the above criteria for a common cold episode, as well as the evaluation of any symptoms by the investigator at V2 (whether they are attributable to common cold), the percentage of subjects with prevention of common cold outburst (upon originally having experienced initial symptoms) will be assessed.

The duration of the cold symptoms is the number of days since start of cold symptoms until the end of the symtpoms (defined as the last day with one or more symptoms followed by at least two symptom-free days (subjects have to answer "No" to the question "Do you think that you are still sick with this respiratory infection?" for 2 days in a row)).

12.7.2 Global Evaluation of the Efficacy by the Subjects

The subjects in the verum group will evaluate the efficacy of the IP (global scaled evaluation with “very good”, “good”, “moderate” and “poor”) at study end.

12.8 ASSESSMENT OF SAFETY

12.8.1 Blood Pressure and Pulse Rate

Sitting blood pressure and pulse rate will be measured using standard products and procedures at all study visits.

12.8.2 Adverse Events

Any AE that occurs during the course of the clinical investigation (for evaluation and reporting see section 13), will be recorded in the CRF.

12.8.3 Global Evaluation of Safety by the Subjects

The subjects in the verum group will evaluate independently the safety of the investigational product by means of a global scaled evaluation with “very good”, “good”, “moderate” and “poor”.

12.9 ASSESSMENT OF FURTHER PARAMETERS

12.9.1 IP usage

Evaluation of IP usage (verum group) will be performed during statistical evaluation at study end, based on the return and documentation of unused investigational product (per vial weight for spray and item counting for capsules) as compared to study duration.

12.10 STUDY DISCONTINUATION

Subjects may withdraw their consent and discontinue their participation in the clinical study at any time, without giving a reason for discontinuation. The investigator may terminate the clinical investigation for single subjects. Further, the sponsor has the right to terminate this clinical investigation.

12.10.1 Withdrawal of Subjects

Reasons for subject’s withdrawal may be for example:

- Serious adverse event (SAE)
- Intolerance of the investigational product
- Required additional therapy due to other complaints, which could influence the safety of the subject or the results of the study
- Clinically significant illness or intake of concurrent medication according to exclusion criteria, which could influence the results of the study
- Subject is not compliant with study requirements (according to investigator judgement)
- Withdrawal of informed consent
- Subjects in the study once the number of treated subjects has been completed

Reason, time and specific details of a subject’s withdrawal are documented in the CRF. Each subject who terminates the study prematurely will be asked to undergo the final visit (corresponding to Visit 3) to be documented in the CRF.

If possible, the IP should be returned by the subject. Subjects who discontinue due to safety reasons should, whenever possible, be seen and assessed by the investigator and be medically treated as appropriate.

12.10.2 Study Discontinuation Criteria

If the clinical investigation has to be discontinued, each subject will be treated as described above.

The sponsor has the right to terminate this clinical investigation, e.g. for the following reasons:

- Serious, insolvable problems with the quality of the data
- Unforeseeable circumstances at the investigational sites, which require discontinuation of the study
- Unacceptable risks

- New scientific or medical knowledge

13 SAFETY EVALUATION AND REPORTING

13.1 DEFINITIONS

13.1.1 Adverse Event (AE) (ISO 14155)

Any untoward medical occurrence, unintended disease or injury, or any untoward clinical signs (including an abnormal laboratory finding) in subjects, users or other persons, whether or not related to the investigational medical device.

This includes events related to the investigational device or the comparator and those related to the procedures involved.

For users or other persons this is restricted to events related to the investigational medical device.

13.1.2 Adverse Device Effect (ADE) (ISO 14155)

Adverse event related to the use of an investigational medical device.

This definition includes adverse events resulting from insufficiencies or inadequacies in the instructions for use, the deployment, the implantation, the installation, the operation, or any malfunction of the investigational medical device.

This includes any event that is a result of a use error or intentional misuse.

13.1.3 Device Deficiency (ISO 14155)

Inadequacy of a medical device with respect to its identity, quality, durability, reliability, safety or performance, such as malfunction, misuse or use error and inadequate labeling.

13.1.4 Serious Adverse Event (SAE) (ISO 14155)

Adverse event that,

- led to death,
- led to serious deterioration in the health of the subject, that either resulted in
  - a life-threatening illness or injury, or
  - a permanent impairment of a body structure or a body function, or
  - in-patient or prolonged hospitalization, or
  - medical or surgical intervention to prevent life-threatening illness or injury or permanent impairment to a body structure or a body function,
- led to foetal distress, foetal death or a congenital abnormality or birth defect.

Planned hospitalization for a pre-existing condition, or a procedure required by the clinical investigation plan (CIP), without serious deterioration in health, is not considered a serious adverse event.

13.1.5 Serious Adverse Device Effect (SADE) (ISO 14155)

Adverse device effect that has resulted in any of the consequences characteristic of a serious adverse event.

13.1.6 Unanticipated Serious Adverse Device Effect (USADE) (ISO 14155)

Serious adverse device effect, which by its nature, incidence, severity or outcome has not been identified in the current version of the risk analysis report.

Anticipated: an effect, which by its nature, incidence, severity or outcome has been previously identified in the risk analysis report. According to the Instruction for use (2016), it is most important not to inhale when applying the spray since this may cause transient asthma-like symptoms, such as coughing and hoarseness; the study subjects will be instructed accordingly (see 10.2).

13.1.7 Incidents (MPSV)

According to the Ordinance on Medical Devices Vigilance (MPSV), an incident is a malfunction, failure or a modification of the features or performance or an inaccurate label or instruction manual for a medical device, which directly or indirectly caused, may have caused in the past, or may cause in the future, death or a serious aggravation of the state of health of a patient, a user or another person.

13.2 DOCUMENTING AND REPORTING ADVERSE EVENTS, ADVERSE DEVICE EFFECTS AND DEVICE DEFICIENCIES

13.2.1 Documenting Adverse Events and Adverse Device Effects by the Investigator

During all examinations, the investigator records any observed AEs and those reported by subjects upon questioning.

If an AE occurs, the seriousness, relationship (not related, unlikely, possible, probable and causal relationship) to the investigational device, time of occurrence and duration of the observed AE, treatment and resolution/outcome will be recorded in the study documentation. All necessary measures are to be taken to determine the cause of the AE and its possible connection to the study.

13.2.2 Documenting and Reporting Device Deficiencies and Incidents by the Investigator

During all examinations after applying the investigational product, the subjects will be asked about any device deficiencies observed. If a device deficiency is reported, the deficiency including the assessment if it may be regarded as an incident will be recorded in the study documentation.

Reportable are all device deficiencies that might have led to a serious adverse event if

a) suitable action had not been taken, or

b) intervention had not been made or

c) if circumstances had been less fortunate, as well as any incidents.

The reporting is handled under the SAE reporting system and will be reported as specified in the form “Report on device deficiencies” (“*Bericht über Mangel des Medizinproduktes / Vorkommnis*”, in the ISF) immediately after becoming aware (but not later than 3 calendar days) to the sponsor and the CRO.

Contacts:

Fredrik Lindberg, MD, PhD;

Phone: +46 46 286 31 00, email: Fredrik.Lindberg@enzymatica.com

Stephanie Seibt;

Phone: +49 30 40008 8 144, Fax: +49 30 40008 501

The initial reports will be followed up until event resolution, or for 14 calendar days after study close-out/subject withdrawal, whichever comes first.

13.2.3 Reporting Serious Adverse Events by the Investigator

All serious adverse events that occur for any reason during the study, will, even when the cause is not connected to the use of the device, be reported by the investigator as specified in the form “Report on serious adverse event” (“*Bericht über schwerwiegende unerwünschte Ereignisse*”, in the ISF) within 24 hours of awareness to the sponsor and the CRO.

Contacts:

Fredrik Lindberg, MD, PhD;

Phone: +46 46 286 31 00, email: Fredrik.Lindberg@enzymatica.com

Stephanie Seibt;

Phone: +49 30 40008 8 144, Fax: +49 30 40008 501

The initial SAE reports will be followed up until event resolution, or for 14 calendar days after study close-out/subject withdrawal, whichever comes first.

13.2.4 Safety Evaluation and Reporting by the Sponsor

The sponsor is responsible for the ongoing safety evaluation of the clinical investigation, reviewing of the device deficiencies, incidents and SAEs reported by the investigator and any further reporting to the Ethics Committee (EC) and the Competent Authority as appropriate.

14 STATISTICS

14.1.1 Objective of the clinical investigation

The main goal of this single (investigator)-blind, randomized, parallel-group pilot clinical investigation is to evaluate the use of various assessments of common cold symptoms for proof of efficacy of CMS016317, by observed magnitude of treatment effects. For details on all endpoints, please refer to section 7.

14.1.2 Statistical hypothesis

The present study is principally intended as an exploratory pilot study. Therefore, explorative data analyses shall be performed, including the calculation of known descriptive statistical quantities for the varied scaled variables under investigation. No prospectively specified statistical hypotheses will be proved, but possibly generated statistical hypotheses from the forecited descriptive analyses; the subsequent statistical tests are therefore only to be understood as exploratory ones, i.e. without any confirmative generalization of the results.

14.1.3 Statistical methods

All end points as well as the concurrent and safety variables will receive an explorative examination and will be descriptively assessed. For the metric data (continuous data) the statistical characteristics will be given (number, mean, standard deviation, median, extremes, quartiles). For ordinal data (discrete data) number, median, interquartile range and extremes will be calculated. If found suitable, ordinal data will be considered as supplementary metric data. For all nominal data the frequency distribution will be presented in frequency tables. The values of metric data can be merged in ordinal classes according to clinical criteria to determine their frequency distribution. Explorative estimation for the structural consistency of the groups regarded will be proved by a descriptive comparison of demographic data, values at screening and secondary (further) endpoints.

In case of generated statistical hypotheses from the above defined descriptive analyses, the subsequent exploratory statistical tests are as follows (for reasons of small samples and/or potentially non-normal distributions):

- Mann-Whitney-U test for comparison independent groups,
- Paired Wilcoxon test for the pre-post comparison within groups,
- Chi-Square test for the comparison of frequencies for independent groups.

All p-values from statistical tests are to be understood as exploratively. That means, the results may deliver interesting insights in the underlying object of research, but those findings don't qualify for a confirmative generalization. Because of the exploratory character of the study no adjustment for multiple testing will be accomplished.

The FAS population will consist of all randomized subjects for which at least one data point after onset of cold symptoms is available, and, for verum group only, who received at least one dose of IP upon onset of cold symptoms. The VCAS population will consist of subjects from FAS without any major violations of the CIP. The assignment to the study populations will be performed before unblinding and start of analysis.

14.1.4 Drop-outs

There will be no replacement of drop-outs after V2.

14.1.5 Sample size estimation

At the current stage of knowledge, no reliable information about the underlying scientific object of research (assessments of common cold symptoms) is available. Therefore, no statistical planning of sample size is possible and the number of 200 subjects is only determined by practical needs of the investigator, taking account for the inter- and intra-individual variability and underlying physiological processes.

The proposed sample size is expectedly required to collect plausible data with respect to the main study goal, the evaluation of various assessments of common cold symptoms for proof of efficacy of CMS016317.

Comparable sample size numbers have been previously reported in common cold studies with natural products (e.g. Auinger et al, 2013; Riede et al, 2013), though these studies were not focussing on the assessment evaluation.

Given the favourable risk-to-benefit-ratio of CMS016317 (see section 6), the study subjects may not be considered as exposed to any substantial risks due to study participation.

14.1.6 Statistical analysis plan/report

Before database closure, a statistical analysis plan (SAP) will be provided, presenting planned statistical analyses as well as any possible deviations or additions of the data analyses originally defined in the CIP before clinical study start. Changes in the planned statistical methods and analyses after approval of the SAP will be documented in the clinical study report. The results of the statistical analyses of the study will be documented in the statistical report. The statistical report will provide the basis for the preparation of the study report.

15 ETHICAL AND LEGAL CONSIDERATIONS

15.1 REGULATORY ASPECTS

This clinical investigation will be performed based on the principles of the World Medical Association (Declaration of Helsinki, version applicable for the involved investigators), ICH GCP E6, German Act on Medical Devices (MPG) §23b and ISO 14155. Further, for reporting of deficiencies, MEDDEV 2.7/3 revision 3 and for reporting of incidents, the Ordinance on Medical Devices Vigilance and MEDDEV 2 12-1 rev. 8 Vigilance will be followed.

15.2 INDEPENDENT ETHICS COMMITTEE

This clinical investigation will be evaluated by an EC before inclusion of the first subject. The CRO will submit the CIP and relevant study documents to the EC to request for opinion.

15.3 SUBJECT INFORMATION AND INFORMED CONSENT FORM

The subject must declare informed consent before participation in the clinical investigation, i.e. before any study specific procedure is performed.

The investigator will ensure that the subject is given full and adequate oral and written information (subject information) about the nature, purpose, consequences and possible risk of the clinical investigation. Subjects must be informed that they are free to withdraw from the study at any time without any resulting disadvantages, how personal and health-related data will be collected and used during the study, and that their identity and medical information will not be disclosed. The subject should be given the opportunity to ask questions and should be allowed sufficient time to consider the information provided. The subject's signed and dated informed consent must be obtained before conducting any study specific procedure. The investigator(s) must store the original, signed and dated informed consent forms in the ISF. A copy of the signed and dated informed consent form must be given to the subject, along with a copy of the insurance conditions.

If new information becomes available that can significantly affect a subject's future health and medical care, that information will be provided to the subject(s) affected in written form. If relevant, all affected subjects will be asked to confirm their continuing informed consent in writing.

15.4 INVESTIGATOR'S OBLIGATIONS

By signing the CIP, the investigator confirms to adhere to the CIP and all applicable national and international regulations and guidelines. The investigator has to ensure that all other study (sub-) investigators and study personnel assisting in the present clinical investigation are qualified by training for their individual responsibilities and functions and informed about the clinical study documents, about the IP, and the investigational procedures.

A delegation log will be set up and updated during the course of the study, in which the investigator will list all study team members including their delegated responsibilities.

The investigators will permit access to source data for study-related monitoring as well as in case of audits and inspections.

15.5 AMENDMENTS TO THE CIP

All amendments to the CIP need agreement between the sponsor and the principal investigator. Every substantial amendment to the CIP or the subject information including the informed consent form needs to be reviewed by the EC prior to implementation. Preparation and submission of any amendments will be performed by the CRO.

Under emergency circumstances, deviations from the CIP to protect the rights, safety and well-being of human subjects may proceed without prior approval of the sponsor and the EC.

15.6 DATA PROTECTION AND CONFIDENTIALITY

The CRO and the study sponsor's representative will ensure the right of the study participants to protection from invasion of privacy. The investigators have to comply with the data protection laws of the Federal Republic of Germany ("Bundesdatenschutzgesetz").

Following consent to participation in the clinical investigation, the subject will be assigned a screening number. Subjects meeting the eligibility criteria will be assigned a random number. The collected data will be made available to the CRO and the study's sponsor only in pseudonymous form, to minimize the chances of matching the data to an individual person. Only the age and gender will be recorded in CRF (no initials, no date of birth). The paper based identification list will be confidentially retained by the investigator.

In case of SAE related documentation provided to the CRO and the study sponsor, the investigator will ensure the pseudonymity of the copies of the documentation provided.

With the receipt of the CIP, the investigator is bound to treat all information contained herein as strictly confidential. He/she is further required to inform his support staff or other personnel that may have access to the CIP of this confidentiality.

16 QUALITY CONTROL AND QUALITY ASSURANCE

Quality assurance and quality control systems will comprise e.g.:

- Training
- Monitoring visits
- Double data entry
- SOPs of the CRO

16.1 TRAINING

The investigator and monitor must make sure that study staff has been appropriately trained and has received the relevant information relating to this study.

16.2 MONITORING

The purpose of monitoring is to verify that the conduct of the clinical investigation complies with the approved CIP, subsequent amendments and applicable regulations. Before the initiation of the clinical investigation, a monitor will verify the adequacy of the investigational site and facilities and discuss with the investigator(s) and other personnel involved in the clinical investigation their responsibilities with regard to the clinical investigation. At the end of the clinical investigation a close-out visit will be performed at the investigational site.

During the clinical investigation, a monitor will have regular contacts with the investigational site, including visits to verify that all data in the CRFs are complete, recorded in a timely manner and consistent with source data, that signed and dated informed consent forms have been obtained from each subject at the time of enrollment and before any clinical study-related procedures are undertaken, that the clinical investigation is being performed according to the CIP, and in accordance with ICH GCP (E6) and applicable regulations.

Further details with respect to monitoring including the extent of source data verification will be laid out in a Monitoring Plan.

16.3 CIP DEVIATIONS

No systematic or prospective deviations from the CIP are tolerated. CIP deviations will be evaluated during blind data review by the sponsor, the CRO, and the biometrician in order to define the study data sets.

16.4 AUDITS AND INSPECTIONS

Authorised representatives of the sponsor and / or regulatory authorities, if applicable, may visit the study centres to perform audits or inspections, including source data verification. The purpose of an audit or inspection is to systematically and independently examine all intervention-related activities and documents to determine whether these activities were conducted, and whether data were recorded, analysed and accurately reported according to the CIP, ICH GCP, ISO 14155 and applicable regulations.

17 DATA HANDLING AND RECORD KEEPING

17.1 SOURCE DATA

All required source data will be recorded in the subject file and subsequently transcribed into CRF. The source data entries must allow to assess whether the study including all procedures was conducted according to CIP. The study diaries and questionnaires act both as source data as well as a part of the CRF.

17.2 DATA DOCUMENTATION

The CRO will provide the investigator with a CRF for each subject. In this CRF, the investigator (or delegate) will in a pseudonymised manner enter all ascertained findings concerning the subject which are documented in the source data. Only the screening and randomization numbers will designate the subject in the CRF.

It is the investigator's responsibility to maintain adequate and accurate CRF to record all observations and other data pertinent to the study. The CRF must be completed with a ballpoint pen, in a correct and legible manner and must be kept current at all times. In no case may corrections be erased or concealed. If a correction should be necessary, the incorrect entry is to be left with a single strike through. The correction is to include the date and signature of the authorized person making the correction.

The investigator confirms by signature the accuracy of all entries in the CRF. The CRF will be checked by the monitor according to the monitoring plan. The investigator should keep subject records separate from the CRF at the investigational site.

17.3 INVESTIGATOR SITE FILE

The investigator will receive a document folder containing the obligations pertaining to him/her during the clinical investigation. It is mainly composed of:

- CIP (and amendments, if applicable)
- Subject information and informed consent
- Ethics committee documents
- CRFs (incl. diaries/questionnaires)
- Form for serious adverse events and report on device deficiencies
- Study initiation monitoring report
- Monitoring log
- Investigational product accountability documentation
- A copy of ICH-GCP (E6) guideline, Declaration of Helsinki
- Screening list
- Subject identification list
- Site signature and delegation sheet
- Clinical investigation insurance documentation

17.4 DATA MANAGEMENT

All collected data will be documented in the CRF and checked by the monitor at the investigational site at regular intervals for plausibility and completeness according to the monitoring

plan. Suspect or missing entries will be explained by a query list and/or questioning the investigator. The validated data will be stored in an appropriate database.

17.5 ARCHIVING

Source data are a component of the subject records and remain at the investigational site. The completed CRFs will be returned to the CRO for data entry and archived along with other study documentation and correspondence concerning the study (Trial Master File) by the sponsor. The ISF including copies of CRFs will remain at the investigational site. The documents will be retained at least 10 years after the end of the clinical investigation.

17.6 PRESENTATION OF DATA – FINAL REPORT

At the end of the clinical investigation, the CRO will write a study report in alignment with the ISO 14155.

18 FINANCING / INSURANCE

The financial sponsor of this clinical investigation is Enzymatica AB. For the event of an injury arising from participation in the clinical investigation, all subjects are covered by a clinical investigation insurance.

19 PUBLICATION POLICY

The results of this clinical investigation may only be published with the written agreement of the study sponsor.

20 LITERATURE

Auinger A, Riede L, Bothe G, Busch R, Gruenwald J. Yeast (1,3)-(1,6)-beta-glucan helps to maintain the body's defence against pathogens: a double-blind, randomized, placebo-controlled, multicentric study in healthy subjects. Eur J Nutr. 2013 Dec;52(8):1913-8.

Clarsund M, Fornbacke M, Uller L, Johnston SL, Emanuelsson CA. (2017) A Randomized, Double-Blind, Placebo-Controlled Pilot Clinical Study on ColdZyme® Mouth Spray against Rhinovirus-Induced Common Cold. Open Journal of Respiratory Diseases, 7, 125-135.

Eccles R, Meier C, Jawad M, Weinmuellner R, Grassauer A, Prieschl-Grassauer E. Efficacy and safety of an antiviral Iota-Carrageenan nasal spray: a randomized, double-blind, placebo-controlled exploratory study in volunteers with early symptoms of the common cold. Respir Res. 2010 Aug 10;11:108. doi: 10.1186/1465-9921-11-108.

Glathe H. Die Influenzaschutzimpfung und ihre Bedeutung für den Individualschutz Medicamentum 5 (1992), 128-135.

Hendley, JO. The host response, not the virus, causes the symptoms of the common cold. Clinical Infectious Diseases, vol. 26, no. 4, pp. 847–848, 1998.

Instruction for Use, 2016.

Jackson GG, Dowling HF, Spiesman IG, Boand AV. Transmission of the common cold to volunteers un-der controlled conditions. Arch Intern Med 1958, 101:267-278.

Johnston, SL. Problems and prospects of developing effective therapy for common cold viruses. Trends in Microbiology, vol. 5, no. 2, pp. 58–63, 1997.

Riede L, Grube B, Gruenwald J. Larch arabinogalactan effects on reducing incidence of upper respiratory infections. Curr Med Res Opin. 2013 Mar;29(3):251-8.

Stefansson B, Gudmundsdottir A, Clarsund M. A medical device forming a protective barrier that deactivates four major common cold viruses, Virol Res Rev, 2017 doi: 10.15761/VRR.1000130 Volume 1(5): 1-3

Tyrrell DAJ. Erkaeltungskrankheit – ein Lehrbuch für die Praxis. G.-Fischer-Verlag, Stuttgart-Jena-New York, 1996.

Wagner H. Pflanzliche Immunstimulanzien. Zur Prophylaxe und Therapie von Erkaeltungskrankheiten. Z Phytother 17 (1996), 79-85.

Ziegler A. Wie gezielt kann Erkaeltungstherapie sein? Aspirin News Letters 7, Nr. 2 (1995), 1-2.

21 SIGNATURES

I declare myself in agreement with this CIP and its appendices.

Study sponsor's representative
Project manager:

Date, Johan Lindvall

Principal investigator/
Medical expert CRO:

Date, Prof. Ralf Uebelhack, MD

Head of Scientific Affairs CRO:

Date, Gordana Bothe, PhD

Project manager CRO:

Date, Stephanie Seibt

Biometrician:

Date, Norman Bitterlich, PhD

21.1 STUDY ADMINISTRATIVE STRUCTURE

Study sponsor
Johan Lindvall
Enzymatica AB
Ideon Science Park, 223 70 Lund, Sweden
Tel: +46 46 286 31 00
email: Johan.Lindvall@enzymatica.com

Project manager CRO
Stephanie Seibt
analyze & realize GmbH
Waldseeweg 6, 13467 Berlin, Germany
Tel: +49 30 40008 144
Fax: +49 30 40008 501
email: sseibt@a-r.com

Principal investigator/Medical expert CRO
Prof. Ralf Uebelhack, MD
analyze & realize GmbH
Weissenseerweg 111, 10369 Berlin, Germany
Tel: +49 30 40008 105
Fax: +49 30 40008 501
email: ruebelhack@a-r.com

Study coordination CRO
Sandra Weiss
analyze & realize GmbH
Waldseeweg 6, 13467 Berlin, Germany
Tel: +49 30 40008 201
Fax: +49 30 40008 501
email: sweiss@a-r.com

Monitoring lead CRO
Yvette Röske, PhD
analyze & realize GmbH
Waldseeweg 6, 13467 Berlin, Germany
Tel: +49 30 40008 104
Fax: +49 30 40008 501
email: yroeske@a-r.com

Data management / Biometry responsible
Norman Bitterlich, PhD
Medicine & Service GmbH
Boettcherstrasse 10, 09117 Chemnitz, Germany
Phone: +49 3 71/560 36 87
Fax: +49 3 71/560 36 99
Email: bitterlich@medizinservice-sachsen.de

21.2 STUDY FLOW CHART

**Procedure/ Assessment**	**Visit 1** **Screening / Randomisation**	**Visit 2** on day 2–3 after start of IP use	**Visit 3** **Final visit*** 16 ± 4 days after start of IP use
Subject information	X		
Written informed consent	X		
Anamnestic, demographic data	X		
Inclusion and exclusion criteria	X		
Medical history/ concurrent diseases	X		
Concurrent treatment	X	X	X
Physical examination	X	X	X
Blood pressure and pulse rate	X	X	X
Documentation of cold symptoms		X	
Urinalysis incl. pregnancy test for women of childbearing potential	X		
Issue of subject diary and instruction	X		
Check of subject diary		X	
Return/control of subject diary			X
Randomisation	X		
Issue of IP** and instruction	X		
Collection of IP and accountability**			X
Adverse events	X	X	X
Device effects		X	X
Global evaluation of efficacy, tolerability and safety by subject			X

* subjects with early termination or without cold symptoms during the entire study duration would only have termnal visit (TV) comprising the assessement of AEs and concurrent treatment as well as the return of IP and diary
** verum group
