# Supplementary material for: Early intervention with a glycerol throat spray containing cold-adapted cod trypsin after self-diagnosis of common cold: A randomised trial
Source: PLoS One. 2022 Jul 5;17(7):e0270699. doi: 10.1371/journal.pone.0270699 (PMC9255730; doi:10.1371/journal.pone.0270699)
Supplement: S2 File — (DOCX) [file pone.0270699.s006.docx]

**PONE-D-21-37327R2**

**Data in manuscript**

- Data reported on rows 208-217. Reference: Table 122 in CI30-018

| **Day** | **V group** | | | **P group** | | | **p_U_ – value** |
| --- | --- | --- | --- | --- | --- | --- | --- |
|  | N | Mean (SD) | Min … Max (Median) | N | Mean (SD) | Min … Max (Median) |  |
| 1 morning | 129 | 7.59 (4.68) | 0 … 20 (7.0) | 127 | 7.46 (5.34) | 0 … 21 (7.0) | 0.654 |
| 1 evening | 136 | 8.74 (4.43) | 1 … 20 (8.0) | 131 | 8.96 (5.00) | 1 … 23 (8.0) | 0.886 |
| 2 morning | 136 | 9.42 (4.52) | 1 … 21 (9.0) | 131 | 9.76 (5.14) | 0 … 24 (9.0) | 0.729 |
| 2 evening | 136 | 9.42 (4.79) | 0 … 24 (9.0) | 131 | 10.30 (5.18) | 0 … 24 (10.0) | 0.176 |
| 3 morning | 136 | 8.29 (5.07) | 0 … 22 (7.0) | 131 | 9.59 (5.31) | 0 … 22 (9.0) | **0.035** |
| 3 evening | 136 | 7.96 (5.15) | 0 … 22 (8.0) | 131 | 9.32 (5.49) | 0 … 23 (9.0) | **0.045** |
| 4 morning | 136 | 6.57 (5.45) | 0 … 23 (6.0) | 131 | 7.86 (5.48) | 0 … 20 (7.0) | **0.038** |
| 4 evening | 136 | 6.05 (5.34) | 0 … 24 (5.0) | 131 | 7.83 (5.65) | 0 … 20 (7.0) | **0.008** |
| 5 morning | 136 | 4.43 (4.66) | 0 … 20 (3.0) | 131 | 6.34 (5.51) | 0 … 20 (5.0) | **0.004** |
| 5 evening | 136 | 4.29 (4.82) | 0 … 23 (3.0) | 131 | 5.89 (5.58) | 0 … 22 (5.0) | **0.019** |
| 6 morning | 136 | 3.42 (4.41) | 0 … 19 (2.0) | 131 | 4.43 (4.80) | 0 … 21 (3.0) | 0.056 |
| 6 evening | 136 | 3.05 (4.09) | 0 … 19 (1.5) | 131 | 4.01 (4.85) | 0 … 23 (2.0) | 0.101 |
| 7 morning | 136 | 2.62 (4.02) | 0 … 16 (0.0) | 131 | 3.08 (4.18) | 0 … 22 (1.0) | 0.197 |
| 7 evening | 136 | 2.43 (4.05) | 0 … 16 (0.0) | 131 | 2.91 (4.31) | 0 … 22 (1.0) | 0.113 |
| 8 morning | 136 | 1.85 (3.58) | 0 … 18 (0.0) | 131 | 2.12 (3.54) | 0 … 18 (0.0) | 0.348 |
| 8 evening | 136 | 1.69 (3.39) | 0 … 18 (0.0) | 131 | 2.04 (3.55) | 0 … 19 (0.0) | 0.297 |
| 9 morning | 136 | 1.27 (2.72) | 0 … 15 (0.0) | 131 | 1.74 (3.15) | 0 … 15 (0.0) | 0.121 |
| 9 evening | 136 | 1.25 (2.82) | 0 … 15 (0.0) | 131 | 1.73 (3.25) | 0 … 17 (0.0) | 0.140 |
| 10 morning | 136 | 0.93 (2.47) | 0 … 13 (0.0) | 131 | 1.31 (2.83) | 0 … 16 (0.0) | 0.067 |
| 10 evening | 136 | 0.91 (2.50) | 0 … 12 (0.0) | 131 | 1.32 (3.14) | 0 … 20 (0.0) | **0.040** |
| 11 morning | 136 | 0.68 (2.04) | 0 … 12 (0.0) | 131 | 1.12 (3.02) | 0 … 20 (0.0) | **0.033** |
| 11 evening | 136 | 0.63 (1.92) | 0 … 11 (0.0) | 131 | 1.10 (3.16) | 0 … 22 (0.0) | **0.023** |
| 12 morning | 136 | 0.46 (1.43) | 0 … 8 (0.0) | 131 | 0.89 (2.84) | 0 … 21 (0.0) | 0.116 |
| 12 evening | 136 | 0.39 (1.35) | 0 … 9 (0.0) | 131 | 0.82 (2.76) | 0 … 20 (0.0) | 0.102 |
| 13 morning | 136 | 0.27 (1.05) | 0 … 7 (0.0) | 131 | 0.71 (2.61) | 0 … 17 (0.0) | 0.424 |
| 13 evening | 136 | 0.22 (0.86) | 0 … 6 (0.0) | 131 | 0.66 (2.49) | 0 … 16 (0.0) | 0.312 |
| 14 morning | 136 | 0.18 (0.86) | 0 … 6 (0.0) | 131 | 0.56 (2.13) | 0 … 16 (0.0) | 0.060 |
| 14 evening | 136 | 0.16 (0.74) | 0 … 6 (0.0) | 131 | 0.57 (2.32) | 0 … 18 (0.0) | 0.179 |
| 15 morning | 136 | 0.12 (0.67) | 0 … 6 (0.0) | 131 | 0.46 (1.92) | 0 … 14 (0.0) | 0.143 |
| 15 evening | 136 | 0.12 (0.62) | 0 … 5 (0.0) | 131 | 0.53 (2.17) | 0 … 15 (0.0) | 0.135 |
| 16 morning | 136 | 0.06 (0.42) | 0 … 4 (0.0) | 131 | 0.47 (1.92) | 0 … 13 (0.0) | **0.013** |
| 16 evening | 136 | 0.03 (0.27) | 0 … 3 (0.0) | 131 | 0.44 (2.01) | 0 … 14 (0.0) | **0.008** |
| 17 morning | 136 | 0.02 (0.26) | 0 … 3 (0.0) | 131 | 0.19 (0.99) | 0 … 9 (0.0) | **0.024** |
| 17 evening | 136 | 0.00 |  | 131 | 0.19 (0.99) | 0 … 9 (0.0) | **0.006** |
| 18 morning | 136 | 0.00 |  | 131 | 0.15 (0.96) | 0 … 10 (0.0) | **0.013** |
| 18 evening | 136 | 0.00 |  | 131 | 0.08 (0.42) | 0 … 3 (0.0) | **0.027** |
| 19 morning | 136 | 0.00 |  | 131 | 0.06 (0.41) | 0 … 3 (0.0) | 0.117 |
| 19 evening | 136 | 0.00 |  | 131 | 0.02 (0.12) | 0 … 1 (0.0) | 0.240 |
| 20 morning | 136 | 0.00 |  | 131 | 0.00 |  | 1.000 |
| 20 evening | 136 | 0.00 |  | 131 | 0.00 |  | 1.000 |

- Data reported on row 219: Reference Table 133 in CI30-018

| **JS: Total score AUC (12 days)** | **N** | **Mean** | **SD** | **Min** | **Q25** | **Median** | **Q75** | **Max** | **CI** |
| --- | --- | --- | --- | --- | --- | --- | --- | --- | --- |
| total | 267 | 49.37 | 34.30 | 1.8 | 23.25 | 40.93 | 66.00 | 186.8 | 45.2 … 53.6 |
| V group | 136 | 45.07 | 32.47 | 2.8 | 22.11 | 36.54 | 58.70 | 157.2 | 39.5 … 50.6 |
| P group | 131 | 53.84 | 35.67 | 1.8 | 26.00 | 49.00 | 73.50 | 186.8 | 47.6 … 60.1 |
| p_U_ value | | 0.023 | | | | | | |  |

- Data reported on row 234: Reference Table 178 in CI30-018

| **Sum Score of WURSS-21**  **AUC (12 days)** | **N** | **Mean** | **SD** | **Min** | **Q25** | **Median** | **Q75** | **Max** | **CI** |
| --- | --- | --- | --- | --- | --- | --- | --- | --- | --- |
| total | 252 | 133.0 | 118.7 | 0.0 | 43.5 | 100.0 | 191.0 | 586.5 | 118.2 … 147.7 |
| V group | 127 | 113.6 | 107.7 | 0.0 | 38.0 | 89.0 | 141.5 | 524.0 | 94.6 … 132.5 |
| P group | 125 | 152.7 | 126.3 | 0.0 | 53.0 | 123.0 | 217.0 | 586.5 | 130.3 … 175.1 |
| p_U_ value | | 0.006 | | | | | | |  |

- Data reported on row 247: Reference Table 1 in CI30-019

| **Duration of cold [days]** | **N** | **Mean** | **SD** | **Min** | **Q25** | **Median** | **Q75** | **Max** | **CI** |
| --- | --- | --- | --- | --- | --- | --- | --- | --- | --- |
| total | 267 | 6.67 | 3.70 | 0 | 4.0 | 6.0 | 8.0 | 19 | 6.3 … 7.8 |
| V group | 136 | 6.26 | 3.27 | 1 | 4.0 | 6.0 | 8.0 | 16 | 5.7 … 6.9 |
| P group | 131 | 7.10 | 4.06 | 0 | 4.0 | 6.0 | 9.5 | 19 | 6.3 … 7.9 |
| p_U_ value | | 0.125 | | | | | | |  |

- Data reported on row 249: Reference Table 128 in CI30-018

| **JS: Total score Duration [days]** | **N** |  | **Mean** | **SD** | **Min** | **Q25** | **Median** | **Q75** | **Max** | **CI** |
| --- | --- | --- | --- | --- | --- | --- | --- | --- | --- | --- |
| total | 267 |  | 7.59 | 3.79 | 1 | 5.0 | 7.0 | 10.0 | 19 | 7.1 … 8.1 |
| V group | 136 |  | 7.11 | 3.37 | 2 | 4.5 | 6.0 | 9.0 | 17 | 6.5 … 7.7 |
| P group | 131 |  | 8.08 | 4.13 | 1 | 5.0 | 7.0 | 11.0 | 19 | 7.3 … 8.8 |
| p_U_ value | |  | 0.071 | | | | | | |  |

- Data reported on row 250: Reference Table 128 in CI30-018

| **Sum score WURSS-21**  **Duration [days]** | **N** | **Mean** | **SD** | **Min** | **Q25** | **Median** | **Q75** | **Max** | **CI** |
| --- | --- | --- | --- | --- | --- | --- | --- | --- | --- |
| total | 252 | 7.03 | 3.88 | 0 | 4.0 | 6.0 | 9.0 | 20 | 6.5 … 7.6 |
| V group | 127 | 6.46 | 3.48 | 0 | 4.0 | 6.0 | 8.0 | 18 | 5.8 … 7.1 |
| P group | 125 | 7.62 | 4.18 | 0 | 4.0 | 7.0 | 10.0 | 20 | 6.8 … 8.4 |
| p_U_ value | | 0.030 | | | | | | |  |

- Data reported on row 264: Reference Table 8 in CI30-019

| **Number of days with CM (7 days)** | **N** | **Mean** | | **SD** | **Min** |  | **Q25** | **Median** | **Q75** | **Max** | **CI** |
| --- | --- | --- | --- | --- | --- | --- | --- | --- | --- | --- | --- |
| total | 267 | 2.7 | | 3.1 | 0 |  | 0.0 | 0.0 | 6.0 | 7 | 2.3 … 3.1 |
| V group | 136 | 2.3 | | 3.0 | 0 |  | 0.0 | 0.0 | 6.0 | 7 | 1.7 … 2.9 |
| P group | 131 | 3.2 | | 3.2 | 0 |  | 0.0 | 0.0 | 7.0 | 7 | 2.6 … 3.8 |
| p_u_ value | |  | 0.032 | | | | | | | |  |

- Data reported on row 265: Reference Table 9 in CI30-019

| **Number of days with CM (14 days)** | **N** | **Mean** | **SD** | **Min** | **Q25** | **Median** | **Q75** | **Max** | **CI** |
| --- | --- | --- | --- | --- | --- | --- | --- | --- | --- |
| total | 267 | 5.8 | 6.6 | 0 | 0.0 | 0.0 | 13.0 | 14 | 5.0 … 6.7 |
| V group | 136 | 5.0 | 6.4 | 0 | 0.0 | 0.0 | 13.0 | 14 | 3.9 … 6.1 |
| P group | 131 | 6.7 | 6.7 | 0 | 0.0 | 11.0 | 14.0 | 14 | 5.5 … 7.9 |
| p_u_ value | | 0.032 | | | | | | |  |

- Data reported on row 270: Reference Table 194 in CI30-018.

| **Global Assessment Efficacy (subjects)** | **V group**  **(N = 115)** | |
| --- | --- | --- |
|  | number | percentage |
| Very good | 54 | 47.0% |
| Good | 41 | 35.7% |
| Moderate | 16 | 13.9% |
| Poor | 4 | 3.5% |

**Data in tables and figures**

**Table 1: Baseline characteristics of the full analysis set (FAS) population**

Reference: Tables 2, 3 and 4 in REP-18031 and tables 8, 10 and 12 in CI30-018.

| **Age [years]** | **N** | **Mean** | **SD** | **MIN** | **Q25** | **Median** | **Q75** | **MAX** |
| --- | --- | --- | --- | --- | --- | --- | --- | --- |
| total | 267 | 40.6 | 13.6 | 18 | 28.0 | 40.0 | 53.0 | 68 |
| **V group** | 136 | 42.7 | 13.8 | 18 | 30.0 | 45.0 | 53.5 | 67 |
| **P group** | 131 | 38.3 | 13.2 | 18 | 28.0 | 37.0 | 49.0 | 68 |
| p-value | 0.010 |  |  |  |  |  |  |  |

| **Gender** | **total (N = 267)** | | **V group (N = 136)** | | **P group (N = 131)** | |
| --- | --- | --- | --- | --- | --- | --- |
|  | **number** | **percentage** | **number** | **percentage** | **Number** | **percentage** |
| **Male** | 77 | 28.8% | 44 | 32.4% | 33 | 25.2% |
| **Female** | 190 | 71.2% | 92 | 67.6% | 98 | 74.8% |
| p-value | | | 0.225 | | | |

| **Ethnicity** | **total (N = 267)** | | **V group (N = 136)** | | **P group (N = 131)** | |
| --- | --- | --- | --- | --- | --- | --- |
|  | **number** | **percentage** | **number** | **percentage** | **Number** | **percentage** |
| **Caucasian** | 259 | 97.0% | 132 | 97.1% | 127 | 96.9% |
| **Asian** | 3 | 1.1% | 2 | 1.5% | 1 | 0.8% |
| **Other** | 5 | 1.9% | 2 | 1.5% | 3 | 2.3% |
| p-value | | | 0.877 | | | |

| **syst. BP**  **[mmHg] v1** | **N** | **Mean** | **SD** | **Min** | | **Q25** | **Median** | **Q75** | **Max** |
| --- | --- | --- | --- | --- | --- | --- | --- | --- | --- |
| total | 267 | 125.8 | 12.0 | 92 | | 118.5 | 125.0 | 133.0 | 160 |
| V group | 136 | 126.6 | 12.0 | 92 | | 120.0 | 126.5 | 135.0 | 160 |
| P group | 131 | 124.8 | 12.1 | 99 | | 116.0 | 125.0 | 131.0 | 160 |
| pu value | | | | | 0.206 | | | | |

| **diast. BP**  **[mmHg] v1** | **N** |  | **Mean** | **SD** | **Min** | | **Q25** | **Median** | **Q75** | **Max** |
| --- | --- | --- | --- | --- | --- | --- | --- | --- | --- | --- |
| total | 267 |  | 77.7 | 7.8 | 55 | | 71.0 | 80.0 | 83.0 | 100 |
| V group | 136 |  | 78.2 | 7.9 | 55 | | 71.0 | 80.0 | 84.0 | 91 |
| P group | 131 |  | 77.0 | 7.6 | 60 | | 70.5 | 78.0 | 81.0 | 100 |
|  | pu value | | | | | 0.135 | | | | |

| **Pulse**  **[bpm] v1** | **N** | **Mean** | **SD** | **Min** | | **Q25** | **Median** | **Q75** | **Max** |
| --- | --- | --- | --- | --- | --- | --- | --- | --- | --- |
| total | 267 | 72.6 | 8.2 | 47 | | 68.0 | 72.0 | 78.0 | 104 |
| V group | 136 | 73.2 | 7.9 | 47 | | 68.0 | 72.0 | 79.0 | 98 |
| P group | 131 | 72.0 | 8.6 | 56 | | 67.0 | 71.0 | 76.5 | 104 |
| pu value | | | | | 0.051 | | | | |

**Table 2: Number of subjects (% of subjects in respective group) without symptoms for each individual item of the Jackson score during the first 8 days of the common cold (mean of morning and evening ratings).**

Reference: Tables 59, 67, 75, 83, 91, 99, 107 and 115 in CI30-018.

| **Subjects without sore throat** | **V group (N = 136)** | | **P group (N = 131)** | | **pexF value** |
| --- | --- | --- | --- | --- | --- |
|  | Number | Percentage | Number | Percentage |  |
| Day 1 | 20 | 14.7% | 19 | 14.5% | 1.000 |
| Day 2 | 12 | 8.8% | 18 | 13.7% | 0.246 |
| Day 3 | 21 | 15.4% | 21 | 16.0% | 1.000 |
| Day 4 | 38 | 27.9% | 33 | 25.2% | 0.678 |
| Day 5 | 68 | 50.0% | 46 | 35.1% | **0.019** |
| Day 6 | 79 | 58.1% | 67 | 51.1% | 0.270 |
| Day 7 | 96 | 70.6% | 82 | 62.6% | 0.194 |
| Day 8 | 110 | 80.9% | 98 | 74.8% | 0.242 |
| Day 9 | 118 | 86.8% | 103 | 78.6% | 0.104 |
| Day 10 | 124 | 91.2% | 110 | 84.0% | 0.094 |
| Day 11 | 127 | 93.4% | 117 | 89.3% | 0.279 |
| Day12 | 130 | 95.6% | 123 | 93.9% | 0.592 |
| Day 13 | 132 | 97.1% | 124 | 94.7% | 0.370 |
| Day 14 | 134 | 98.5% | 123 | 93.9% | 0.057 |
| Day 15 | 135 | 99.3% | 125 | 95.4% | 0.062 |
| Day 16 | 136 | 100% | 125 | 95.4% | **0.013** |
| Day 17 | 136 | 100% | 128 | 97.7% | 0.117 |
| Day 18 | 136 | 100% | 128 | 97.7% | 0.117 |
| Day 19 | 136 | 100% | 129 | 98.5% | 0.240 |
| Day 20 | 136 | 100% | 131 | 100% | 1.000 |

| **Subjects without blocked nose** | **V group (N = 136)** | | **P group (N = 131)** | | **pexF value** |
| --- | --- | --- | --- | --- | --- |
|  | Number | Percentage | Number | Percentage |  |
| Day 1 | 38 | 27.9% | 40 | 30.5% | 0.687 |
| Day 2 | 34 | 25.0% | 30 | 22.9% | 0.775 |
| Day 3 | 43 | 31.6% | 25 | 19.1% | **0.024** |
| Day 4 | 53 | 39.0% | 36 | 27.5% | 0.052 |
| Day 5 | 67 | 49.3% | 53 | 40.5% | 0.176 |
| Day 6 | 82 | 60.3% | 72 | 55.0% | 0.389 |
| Day 7 | 93 | 68.4% | 83 | 63.4% | 0.439 |
| Day 8 | 104 | 76.5% | 94 | 71.8% | 0.575 |
| Day 9 | 109 | 80.1% | 96 | 73.3% | 0.195 |
| Day 10 | 121 | 89.0% | 105 | 80.2% | 0.061 |
| Day 11 | 123 | 90.4% | 109 | 83.2% | 0.102 |
| Day12 | 128 | 94.1% | 119 | 90.8% | 0.358 |
| Day 13 | 130 | 95.6% | 119 | 90.8% | 0.146 |
| Day 14 | 132 | 97.1% | 120 | 91.6% | 0.065 |
| Day 15 | 133 | 97.8% | 123 | 93.9% | 0.131 |
| Day 16 | 135 | 99.3% | 123 | 93.9% | **0.018** |
| Day 17 | 136 | 100% | 128 | 97.7% | 0.117 |
| Day 18 | 136 | 100% | 129 | 98.5% | 0.240 |
| Day 19 | 136 | 100% | 130 | 99.2% | 0.491 |
| Day 20 | 136 | 100% | 131 | 100% | 1.000 |

| **Subjects without**  **runny nose** | **V group (N = 136)** | | **P group (N = 131)** | | **pexF value** |
| --- | --- | --- | --- | --- | --- |
|  | Number | Percentage | Number | Percentage |  |
| Day 1 | 42 | 30.9% | 41 | 31.3% | 1.000 |
| Day 2 | 33 | 24.3% | 32 | 24.4% | 1.000 |
| Day 3 | 38 | 27.9% | 32 | 24.4% | 0.578 |
| Day 4 | 51 | 37.5% | 39 | 29.8% | 0.197 |
| Day 5 | 65 | 47.8% | 54 | 41.2% | 0.325 |
| Day 6 | 81 | 59.6% | 69 | 52.7% | 0.269 |
| Day 7 | 88 | 64.7% | 82 | 62.6% | 0.799 |
| Day 8 | 103 | 75.7% | 95 | 72.5% | 0.578 |
| Day 9 | 114 | 83.8% | 101 | 77.1% | 0.216 |
| Day 10 | 118 | 86.8% | 107 | 81.7% | 0.313 |
| Day 11 | 125 | 91.9% | 111 | 84.7% | 0.085 |
| Day 12 | 129 | 94.9% | 115 | 87.8% | **0.049** |
| Day 13 | 131 | 96.3% | 121 | 92.4% | 0.190 |
| Day 14 | 130 | 95.6% | 122 | 93.1% | 0.434 |
| Day 15 | 132 | 97.1% | 124 | 94.7% | 0.370 |
| Day 16 | 134 | 98.5% | 124 | 94.7% | 0.098 |
| Day 17 | 135 | 99.3% | 127 | 96.9% | 0.207 |
| Day 18 | 136 | 100% | 129 | 98.5% | 0.240 |
| Day 19 | 136 | 100% | 130 | 99.2% | 0.491 |
| Day 20 | 136 | 100% | 131 | 100% | 1.000 |

| **Subjects without cough** | **V group (N = 136)** | | **P group (N = 131)** | | **pexF value** |
| --- | --- | --- | --- | --- | --- |
|  | Number | Percentage | Number | Percentage |  |
| Day 1 | 46 | 33.8% | 55 | 42.0% | 0.207 |
| Day 2 | 29 | 21.3% | 41 | 31.3% | 0.071 |
| Day 3 | 33 | 24.3% | 33 | 25.2% | 0.888 |
| Day 4 | 56 | 41.2% | 38 | 29.0% | **0.041** |
| Day 5 | 74 | 54.4% | 51 | 38.9% | **0.014** |
| Day 6 | 86 | 63.2% | 59 | 45.0% | **0.003** |
| Day 7 | 93 | 68.4% | 74 | 56.5% | 0.076 |
| Day 8 | 102 | 75.0% | 93 | 71.0% | 0.492 |
| Day 9 | 108 | 79.4% | 94 | 71.8% | 0.156 |
| Day 10 | 116 | 85.3% | 100 | 76.3% | 0.086 |
| Day 11 | 120 | 88.2% | 103 | 78.6% | **0.047** |
| Day 12 | 125 | 91.9% | 110 | 84.0% | **0.059** |
| Day 13 | 129 | 94.9% | 117 | 89.3% | 0.113 |
| Day 14 | 131 | 96.3% | 121 | 92.4% | 0.190 |
| Day 15 | 132 | 97.1% | 121 | 92.4% | 0.103 |
| Day 16 | 134 | 98.5% | 121 | 92.4% | **0.018** |
| Day 17 | 135 | 99.3% | 124 | 94.7% | **0.033** |
| Day 18 | 136 | 100% | 125 | 95.4% | **0.013** |
| Day 19 | 136 | 100% | 129 | 98.5% | 0.240 |
| Day 20 | 136 | 100% | 131 | 100% | 1.000 |

| **Subjects without sneezing** | **V group (N = 136)** | | **P group (N = 131)** | | **pexF value** |
| --- | --- | --- | --- | --- | --- |
|  | Number | Percentage | Number | Percentage |  |
| Day 1 | 31 | 22.8% | 44 | 33.6% | 0.057 |
| Day 2 | 36 | 26.5% | 35 | 26.7% | 1.000 |
| Day 3 | 42 | 30.9% | 36 | 27.5% | 0.591 |
| Day 4 | 65 | 47.8% | 52 | 39.7% | 0.217 |
| Day 5 | 90 | 66.2% | 67 | 51.1% | **0.013** |
| Day 6 | 104 | 76.5% | 81 | 61.8% | **0.012** |
| Day 7 | 107 | 78.7% | 98 | 74.8% | 0.472 |
| Day 8 | 116 | 85.3% | 113 | 86.3% | 0.862 |
| Day 9 | 123 | 90.4% | 115 | 87.8% | 0.557 |
| Day 10 | 129 | 94.9% | 120 | 91.6% | 0.335 |
| Day 11 | 130 | 95.6% | 124 | 94.7% | 0.782 |
| Day 12 | 130 | 95.6% | 125 | 95.4% | 1.000 |
| Day 13 | 134 | 98.5% | 125 | 95.4% | 0.166 |
| Day 14 | 133 | 97.8% | 125 | 95.4% | 0.327 |
| Day 15 | 135 | 99.3% | 126 | 96.2% | 0.115 |
| Day 16 | 136 | 100% | 125 | 95.4% | **0.013** |
| Day 17 | 136 | 100% | 130 | 99.2% | 0.491 |
| Day 18 | 136 | 100% | 130 | 99.2% | 0.491 |
| Day 19 | 136 | 100% | 131 | 100% | 1.000 |
| Day 20 | 136 | 100% | 131 | 100% | 1.000 |

| **Subjects without headache** | **V group (N = 136)** | | **P group (N = 131)** | | **pexF value** |
| --- | --- | --- | --- | --- | --- |
|  | Number | Percentage | Number | Percentage |  |
| Day 1 | 47 | 34.6% | 37 | 28.2% | 0.293 |
| Day 2 | 45 | 33.1% | 34 | 26.0% | 0.228 |
| Day 3 | 47 | 34.6% | 32 | 24.4% | 0.082 |
| Day 4 | 67 | 49.3% | 51 | 38.9% | 0.109 |
| Day 5 | 92 | 67.6% | 62 | 47.3% | **0.001** |
| Day 6 | 104 | 76.5% | 83 | 63.4% | **0.023** |
| Day 7 | 116 | 85.3% | 101 | 77.1% | 0.116 |
| Day 8 | 120 | 88.2% | 110 | 84.0% | 0.377 |
| Day 9 | 122 | 89.7% | 113 | 86.3% | 0.453 |
| Day 10 | 125 | 91.9% | 125 | 95.4% | 0.318 |
| Day 11 | 129 | 94.9% | 122 | 93.1% | 0.613 |
| Day 12 | 129 | 94.9% | 126 | 96.2% | 0.770 |
| Day 13 | 132 | 97.1% | 125 | 95.4% | 0.534 |
| Day 14 | 134 | 98.5% | 125 | 95.4% | 0.166 |
| Day 15 | 133 | 97.8% | 126 | 96.2% | 0.494 |
| Day 16 | 134 | 98.5% | 126 | 96.2% | 0.275 |
| Day 17 | 136 | 100% | 129 | 98.5% | 0.240 |
| Day 18 | 136 | 100% | 129 | 98.5% | 0.240 |
| Day 19 | 136 | 100% | 130 | 99.2% | 0.491 |
| Day 20 | 136 | 100% | 131 | 100% | 1.000 |

| **Subjects without malaise** | **V group (N = 136)** | | **P group (N = 131)** | | **pexF value** |
| --- | --- | --- | --- | --- | --- |
|  | Number | Percentage | Number | Percentage |  |
| Day 1 | 48 | 35.3% | 45 | 34.4% | 0.898 |
| Day 2 | 40 | 29.4% | 37 | 28.2% | 0.893 |
| Day 3 | 47 | 34.6% | 41 | 31.3% | 0.604 |
| Day 4 | 65 | 47.8% | 58 | 44.3% | 0.624 |
| Day 5 | 84 | 61.8% | 65 | 49.6% | **0.049** |
| Day 6 | 101 | 74.3% | 86 | 65.6% | 0.142 |
| Day 7 | 111 | 81.6% | 99 | 75.6% | 0.236 |
| Day 8 | 121 | 89.0% | 108 | 82.4% | 0.161 |
| Day 9 | 125 | 91.9% | 115 | 87.8% | 0.312 |
| Day 10 | 127 | 93.4% | 121 | 92.4% | 0.815 |
| Day 11 | 127 | 93.4% | 123 | 93.9% | 1.000 |
| Day 12 | 128 | 94.1% | 126 | 96.2% | 0.572 |
| Day 13 | 131 | 96.3% | 127 | 96.9% | 1.000 |
| Day 14 | 135 | 99.3% | 126 | 96.2% | 0.115 |
| Day 15 | 135 | 99.3% | 125 | 95.4% | 0.062 |
| Day 16 | 135 | 99.3% | 126 | 96.2% | 0.115 |
| Day 17 | 136 | 100% | 130 | 99.2% | 0.491 |
| Day 18 | 136 | 100% | 130 | 99.2% | 0.491 |
| Day 19 | 136 | 100% | 131 | 100% | 1.000 |
| Day 20 | 136 | 100% | 131 | 100% | 1.000 |

| **Subjects without chilliness** | **V group (N = 136)** | | **P group (N = 131)** | | **pexF value** |
| --- | --- | --- | --- | --- | --- |
|  | Number | Percentage | Number | Percentage |  |
| Day 1 | 72 | 52.9% | 70 | 53.4% | 1.000 |
| Day 2 | 72 | 52.9% | 71 | 54.2% | 0.902 |
| Day 3 | 86 | 63.2% | 78 | 59.5% | 0.615 |
| Day 4 | 108 | 79.4% | 95 | 72.5% | 0.200 |
| Day 5 | 114 | 83.8% | 102 | 77.9% | 0.276 |
| Day 6 | 126 | 92.6% | 116 | 88.5% | 0.296 |
| Day 7 | 127 | 93.4% | 120 | 91.6% | 0.646 |
| Day 8 | 128 | 94.1% | 124 | 94.7% | 1.000 |
| Day 9 | 131 | 96.3% | 126 | 96.2% | 1.000 |
| Day 10 | 132 | 97.1% | 127 | 96.9% | 1.000 |
| Day 11 | 132 | 97.1% | 127 | 96.9% | 1.000 |
| Day 12 | 134 | 98.5% | 128 | 97.7% | 0.679 |
| Day 13 | 134 | 98.5% | 128 | 97.7% | 0.679 |
| Day 14 | 136 | 100% | 129 | 98.5% | 0.240 |
| Day 15 | 135 | 99.3% | 127 | 96.9% | 0.207 |
| Day 16 | 136 | 100% | 128 | 97.7% | 0.117 |
| Day 17 | 136 | 100% | 131 | 100% | 1.000 |
| Day 18 | 136 | 100% | 131 | 100% | 1.000 |
| Day 19 | 136 | 100% | 131 | 100% | 1.000 |
| Day 20 | 136 | 100% | 131 | 100% | 1.000 |

**Table 3: Number of subjects (% of subjects in respective group) without symptoms for each individual item of the 9-item WURSS-21 QoL domain during the first 8 days of the common cold (evening ratings).**

Reference: Tables 155, 166, 177, 188, 199, 210, 221, 232 and 243 in CI30-019.

| **Subjects without symptoms of item 12** | **V group (N = 127)** | | **P group (N = 125)** | | **pexF value** |
| --- | --- | --- | --- | --- | --- |
|  | Number | Percentage | Number | Percentage |  |
| Day 1 | 63 | 49.6% | 54 | 43.2% | 0.316 |
| Day 2 | 51 | 40.2% | 34 | 27.2% | **0.033** |
| Day 3 | 58 | 45.7% | 37 | 29.6% | **0.009** |
| Day 4 | 68 | 53.5% | 44 | 35.2% | **0.002** |
| Day 5 | 83 | 65.4% | 52 | 41.6% | **<0.001** |
| Day 6 | 90 | 70.9% | 65 | 52.0% | **0.003** |
| Day 7 | 98 | 77.2% | 84 | 67.2% | 0.092 |
| Day 8 | 106 | 83.5% | 92 | 73.6% | 0.066 |

| **Subjects without symptoms of item 13** | **V group (N = 127)** | | **P group (N = 125)** | | **pexF value** |
| --- | --- | --- | --- | --- | --- |
|  | Number | Percentage | Number | Percentage |  |
| Day 1 | 29 | 22.8% | 24 | 19.2% | 0.538 |
| Day 2 | 21 | 16.5% | 12 | 9.6% | 0.135 |
| Day 3 | 28 | 22.0% | 21 | 16.8% | 0.341 |
| Day 4 | 40 | 31.5% | 27 | 21.6% | **0.088** |
| Day 5 | 57 | 44.9% | 38 | 30.4% | **0.020** |
| Day 6 | 69 | 54.3% | 47 | 37.6% | **0.008** |
| Day 7 | 83 | 65.4% | 61 | 48.8% | **0.011** |
| Day 8 | 95 | 74.8% | 78 | 62.4% | **0.042** |

| **Subjects without symptoms of item 14** | **V group (N = 127)** | | **P group (N = 125)** | | **pexF value** |
| --- | --- | --- | --- | --- | --- |
|  | Number | Percentage | Number | Percentage |  |
| Day 1 | 20 | 15.7% | 22 | 17.6% | 0.737 |
| Day 2 | 18 | 14.2% | 15 | 12.0% | 0.710 |
| Day 3 | 26 | 20.5% | 18 | 14.4% | 0.246 |
| Day 4 | 42 | 33.1% | 28 | 22.4% | 0.068 |
| Day 5 | 55 | 43.3% | 39 | 31.2% | 0.052 |
| Day 6 | 61 | 48.0% | 47 | 37.6% | 0.100 |
| Day 7 | 80 | 63.0% | 59 | 47.2% | **0.016** |
| Day 8 | 89 | 70.1% | 78 | 62.4% | 0.231 |

| **Subjects without symptoms of item 15** | **V group (N = 127)** | | **P group (N = 125)** | | **pexF value** |
| --- | --- | --- | --- | --- | --- |
|  | Number | Percentage | Number | Percentage |  |
| Day 1 | 48 | 37.8% | 36 | 28.8% | 0.143 |
| Day 2 | 37 | 29.1% | 26 | 20.8% | 0.146 |
| Day 3 | 44 | 34.6% | 29 | 23.2% | 0.052 |
| Day 4 | 59 | 46.5% | 31 | 24.8% | **<0.001** |
| Day 5 | 70 | 55.1% | 43 | 34.4% | **0.001** |
| Day 6 | 84 | 66.1% | 55 | 44.4% | **0.001** |
| Day 7 | 95 | 74.8% | 73 | 58.4% | **0.007** |
| Day 8 | 104 | 81.9% | 89 | 71.2% | 0.053 |

| **Subjects without symptoms of item 16** | **V group (N = 127)** | | **P group (N = 125)** | | **pexF value** |
| --- | --- | --- | --- | --- | --- |
|  | Number | Percentage | Number | Percentage |  |
| Day 1 | 46 | 36.2% | 36 | 28.8% | 0.228 |
| Day 2 | 33 | 26.0% | 26 | 20.8% | 0.373 |
| Day 3 | 43 | 33.9% | 29 | 23.2% | 0.070 |
| Day 4 | 63 | 49.6% | 36 | 28.8% | **0.001** |
| Day 5 | 77 | 60.6% | 50 | 40.0% | **0.002** |
| Day 6 | 89 | 70.1% | 58 | 46.4% | **<0.001** |
| Day 7 | 97 | 76.4% | 78 | 62.4% | **0.020** |
| Day 8 | 106 | 83.5% | 93 | 74.4% | 0.090 |

| **Subjects without symptoms of item 17** | **V group (N = 127)** | | **P group (N = 125)** | | **pexF value** |
| --- | --- | --- | --- | --- | --- |
|  | Number | Percentage | Number | Percentage |  |
| Day 1 | 50 | 39.4% | 39 | 31.2% | 0.189 |
| Day 2 | 41 | 32.3% | 24 | 19.2% | **0.021** |
| Day 3 | 47 | 37.0% | 34 | 27.2% | 0.107 |
| Day 4 | 61 | 48.0% | 38 | 30.4% | **0.005** |
| Day 5 | 77 | 60.6% | 49 | 39.2% | **0.001** |
| Day 6 | 91 | 71.7% | 60 | 48.0% | **<0.001** |
| Day 7 | 97 | 76.4% | 76 | 60.8% | **0.010** |
| Day 8 | 107 | 84.3% | 91 | 72.8% | **0.032** |

| **Subjects without symptoms of item 18** | **V group (N = 127)** | | **P group (N = 125)** | | **pexF value** |
| --- | --- | --- | --- | --- | --- |
|  | Number | Percentage | Number | Percentage |  |
| Day 1 | 51 | 40.2% | 40 | 32.0% | 0.192 |
| Day 2 | 40 | 31.5% | 28 | 22.4% | 0.119 |
| Day 3 | 46 | 36.2% | 34 | 27.2% | 0.138 |
| Day 4 | 66 | 52.0% | 41 | 32.8% | **0.002** |
| Day 5 | 82 | 64.6% | 55 | 44.0% | **0.002** |
| Day 6 | 91 | 71.7% | 62 | 49.6% | **<0.001** |
| Day 7 | 99 | 78.0% | 80 | 64.0% | **0.018** |
| Day 8 | 109 | 85.8% | 95 | 76.0% | 0.055 |

| **Subjects without symptoms of item 19** | **V group (N = 127)** | | **P group (N = 125)** | | **pexF value** |
| --- | --- | --- | --- | --- | --- |
|  | Number | Percentage | Number | Percentage |  |
| Day 1 | 49 | 38.6% | 42 | 33.6% | 0.433 |
| Day 2 | 51 | 40.2% | 34 | 27.2% | **0.033** |
| Day 3 | 51 | 40.2% | 35 | 28.0% | **0.047** |
| Day 4 | 67 | 52.8% | 41 | 32.8% | **0.002** |
| Day 5 | 77 | 60.6% | 55 | 44.0% | **0.011** |
| Day 6 | 90 | 70.9% | 65 | 52.0% | **0.003** |
| Day 7 | 95 | 74.8% | 83 | 66.4% | 0.167 |
| Day 8 | 106 | 83.5% | 95 | 76.0% | 0.160 |

| **Subjects without symptoms of item 20** | **V group (N = 127)** | | **P group (N = 125)** | | **pexF value** |
| --- | --- | --- | --- | --- | --- |
|  | Number | Percentage | Number | Percentage |  |
| Day 1 | 48 | 37.8% | 43 | 34.4% | 0.602 |
| Day 2 | 43 | 33.9% | 33 | 26.4% | 0.218 |
| Day 3 | 53 | 41.7% | 35 | 28.0% | **0.025** |
| Day 4 | 68 | 53.6% | 42 | 33.6% | **0.002** |
| Day 5 | 83 | 65.4% | 56 | 44.8% | **0.001** |
| Day 6 | 92 | 72.4% | 71 | 56.8% | **0.012** |
| Day 7 | 98 | 77.2% | 88 | 70.4% | 0.253 |
| Day 8 | 108 | 85.0% | 98 | 78.4% | 0.194 |

**Fig 1: Trial profile**

Based on the format of the CONSORT Flow Diagram, adapted from Table 1 of CI30-020.

| **Analysis population** | **total** | | **V group** | | **P group** | |
| --- | --- | --- | --- | --- | --- | --- |
|  | number | percentage | number | percentage | number | percentage |
| total | 400 | 100% | 200 | 50.0% | 200 | 50.0% |
| FAS | 267 | 66.8% | 136 | 50.9% | 131 | 49.1% |
| VCAS | 261 | 65.2% | 131 | 50.2% | 130 | 49.8% |

**Fig 2: Forest plot analyses of individual items of 9-item WURSS-21 QoL domain and 8-item Jackson symptom ratings**

References: Jackson score: Tables 65, 73, 81, 89, 97, 105, 113 and 121 in CI30-018.

9-item WURSS QoL: Tables 162, 173, 184, 195, 206, 217, 228, 239 and 250 in CI30-019.

Individual items of the WURSS-21 and Jackson scores were compared between the treatment groups using raw data and normal deviate tests (z-tests) of the mean AUCs. The results are presented in the forest plot above produced by the package forest plot with R version 3.5.2.

| **JS: Score throat AUC (12 days)** | **N** | **Mean** | **SD** | **Min** | **Q25** | **Median** | **Q75** | **Max** | **CI** |
| --- | --- | --- | --- | --- | --- | --- | --- | --- | --- |
| total | 267 | 7.94 | 5.74 | 0.0 | 3.50 | 7.00 | 11.88 | 28.8 | 7.2 … 8.7 |
| V group | 136 | 7.13 | 5.15 | 0.0 | 3.25 | 6.00 | 9.12 | 23.8 | 6.2 … 8.1 |
| P group | 131 | 8.78 | 6.20 | 0.0 | 4.00 | 7.75 | 12.50 | 28.8 | 7.7 … 9.9 |
| pu value | | 0.035 | | | | | | |  |

| **JS: Blocked nose AUC (12 days)** | **N** | **Mean** | **SD** | **Min** | **Q25** | **Median** | **Q75** | **Max** | **CI** |
| --- | --- | --- | --- | --- | --- | --- | --- | --- | --- |
| total | 267 | 7.51 | 6.26 | 0.0 | 2.75 | 6.75 | 11.25 | 31.8 | 6.7 … 8.3 |
| V group | 136 | 6.74 | 6.00 | 0.0 | 1.25 | 5.50 | 10.50 | 26.2 | 5.7 … 7.8 |
| P group | 131 | 8.31 | 6.44 | 0.0 | 3.50 | 7.50 | 11.62 | 31.8 | 7.1 … 9.5 |
| pu value | | 0.038 | | | | | | |  |

| **JS: Runny nose AUC (12 days)** | **N** | **Mean** | **SD** | **Min** | **Q25** | **Median** | **Q75** | **Max** | **CI** |
| --- | --- | --- | --- | --- | --- | --- | --- | --- | --- |
| total | 267 | 7.17 | 5.64 | 0.0 | 2.75 | 6.50 | 10.50 | 27.8 | 6.4 … 7.9 |
| V group | 136 | 6.61 | 5.36 | 0.0 | 2.62 | 5.50 | 9.38 | 27.8 | 5.7 … 7.6 |
| P group | 131 | 7.74 | 5.89 | 0.0 | 2.88 | 7.50 | 11.88 | 24.8 | 6.7 … 8.8 |
| pu value | | 0.131 | | | | | | |  |

| **JS: Cough AUC (12 days)** | **N** | **Mean** | **SD** | **Min** | **Q25** | **Median** | **Q75** | **Max** | **CI** |
| --- | --- | --- | --- | --- | --- | --- | --- | --- | --- |
| total | 267 | 7.79 | 7.11 | 0.0 | 2.00 | 6.00 | 11.00 | 31.5 | 6.9 … 8.7 |
| V group | 136 | 6.99 | 6.40 | 0.0 | 2.00 | 5.50 | 9.75 | 29.8 | 5.9 … 8.1 |
| P group | 131 | 8.62 | 7.72 | 0.0 | 2.00 | 7.50 | 13.00 | 31.5 | 7.2 … 10.0 |
| pu value | | 0.132 | | | | | | |  |

| **JS: Sneezing AUC (12 days)** | **N** | **Mean** | **SD** | **Min** | **Q25** | **Median** | **Q75** | **Max** | **CI** |
| --- | --- | --- | --- | --- | --- | --- | --- | --- | --- |
| total | 267 | 4.93 | 4.35 | 0.0 | 1.50 | 4.00 | 7.00 | 18.8 | 4.4 … 5.5 |
| V group | 136 | 4.57 | 4.22 | 0.0 | 1.50 | 3.50 | 6.25 | 18.5 | 3.8 … 5.3 |
| P group | 131 | 5.31 | 4.46 | 0.0 | 2.00 | 4.50 | 7.75 | 18.8 | 4.5 … 6.1 |
| pu value | | 0.141 | | | | | | |  |

| **JS: Headache AUC (12 days)** | **N** | **Mean** | **SD** | **Min** | **Q25** | **Median** | **Q75** | **Max** | **CI** |
| --- | --- | --- | --- | --- | --- | --- | --- | --- | --- |
| total | 267 | 5.47 | 5.24 | 0.0 | 1.25 | 4.25 | 7.88 | 27.8 | 4.8 … 6.2 |
| V group | 136 | 5.06 | 5.58 | 0.0 | 0.75 | 3.50 | 7.00 | 27.8 | 4.1 … 6.1 |
| P group | 131 | 5.91 | 4.85 | 0.0 | 2.50 | 5.00 | 8.75 | 24.2 | 5.0 … 6.8 |
| pu value | | 0.025 | | | | | | |  |

| **JS: Malaise AUC (12 days)** | **N** | **Mean** | **SD** | **Min** | **Q25** | **Median** | **Q75** | **Max** | **CI** |
| --- | --- | --- | --- | --- | --- | --- | --- | --- | --- |
| total | 267 | 5.61 | 5.81 | 0.0 | 1.12 | 4.00 | 7.75 | 31.0 | 4.9 … 6.4 |
| V group | 136 | 5.16 | 5.60 | 0.0 | 0.50 | 4.00 | 7.50 | 31.0 | 4.2 … 6.2 |
| P group | 131 | 6.08 | 6.01 | 0.0 | 1.62 | 4.50 | 8.62 | 26.5 | 5.0 … 7.2 |
| pu value | | 0.183 | | | | | | |  |

| **JS: Chilliness AUC (12 days)** | **N** | **Mean** | **SD** | **Min** | **Q25** | **Median** | **Q75** | **Max** | **CI** |
| --- | --- | --- | --- | --- | --- | --- | --- | --- | --- |
| total | 267 | 2.70 | 4.17 | 0.0 | 0.00 | 1.00 | 3.50 | 25.0 | 2.2 … 3.3 |
| V group | 136 | 2.61 | 4.31 | 0.0 | 0.00 | 1.00 | 3.38 | 25.0 | 1.8 … 3.4 |
| P group | 131 | 2.80 | 4.04 | 0.0 | 0.00 | 1.00 | 4.25 | 22.8 | 2.1 … 3.6 |
| pu value | |  | | | | | | |  |

| **Item 12 WURSS-21**  **AUC (12 days)** | **N** | **Mean** | **SD** | **Min** | **Q25** | **Median** | **Q75** | **Max** | **CI** |
| --- | --- | --- | --- | --- | --- | --- | --- | --- | --- |
| total | 252 | 12.0 | 13.5 | 0.0 | 1.0 | 7.5 | 18.0 | 67.5 | 10.2 … 13.7 |
| V group | 127 | 10.6 | 13.8 | 0.0 | 0.0 | 5.0 | 15.0 | 67.0 | 8.2 … 13.1 |
| P group | 125 | 13.3 | 13.2 | 0.0 | 3.0 | 9.0 | 20.0 | 67.5 | 11.0 … 15.7 |
| p_U_ value | | 0.008 | | | | | | |  |
| **Item 13 WURSS-21**  **AUC (12 days)** | **N** | **Mean** | **SD** | **Min** | **Q25** | **Median** | **Q75** | **Max** | **CI** |
| total | 252 | 19.25 | 14.82 | 0.0 | 8.00 | 15.75 | 28.50 | 70.0 | 17.4 … 21.1 |
| V group | 127 | 17.29 | 13.93 | 0.0 | 8.00 | 13.00 | 25.00 | 70.0 | 14.8 … 19.7 |
| P group | 125 | 21.25 | 15.28 | 0.0 | 9.00 | 20.00 | 31.50 | 64.5 | 18.5 … 24.0 |
| p_U_ value | | 0.039 | | | | | | |  |

| **Item 14 WURSS-21**  **AUC (12 days)** | **N** | **Mean** | **SD** | **Min** | **Q25** | **Median** | **Q75** | **Max** | **CI** |
| --- | --- | --- | --- | --- | --- | --- | --- | --- | --- |
| total | 252 | 19.70 | 14.33 | 0.0 | 9.00 | 16.50 | 28.50 | 67.5 | 17.9 … 21.5 |
| V group | 127 | 17.40 | 12.73 | 0.0 | 7.50 | 15.00 | 25.00 | 50.5 | 15.1 … 19.7 |
| P group | 125 | 22.04 | 15.49 | 0.0 | 10.00 | 20.00 | 30.00 | 67.5 | 19.2 … 24.8 |
| p_U_ value | | 0.023 | | | | | | |  |

| **Item 15 WURSS-21**  **AUC (12 days)** | **N** | **Mean** | **SD** | **Min** | **Q25** | **Median** | **Q75** | **Max** | **CI** |
| --- | --- | --- | --- | --- | --- | --- | --- | --- | --- |
| total | 252 | 14.62 | 14.36 | 0.0 | 3.00 | 11.00 | 21.75 | 65.5 | 12.8 … 16.5 |
| V group | 127 | 11.86 | 12.68 | 0.0 | 2.00 | 8.00 | 17.00 | 59.0 | 9.6 … 14.1 |
| P group | 125 | 17.43 | 15.44 | 0.0 | 6.00 | 14.00 | 26.00 | 65.5 | 14.8 … 20.2 |
| p_U_ value | | 0.001 | | | | | | |  |

| **Item 16 WURSS-21**  **AUC (12 days)** | **N** | **Mean** | **SD** | **Min** | **Q25** | **Median** | **Q75** | **Max** | **CI** |
| --- | --- | --- | --- | --- | --- | --- | --- | --- | --- |
| total | 252 | 13.86 | 14.15 | 0.0 | 3.00 | 10.00 | 20.50 | 65.5 | 12.1 … 15.7 |
| V group | 127 | 11.67 | 12.80 | 0.0 | 2.00 | 8.00 | 15.00 | 59.0 | 9.4 … 14.0 |
| P group | 125 | 16.09 | 15.13 | 0.0 | 4.00 | 13.00 | 23.00 | 65.5 | 13.4 … 18.8 |
| p_U_ value | | 0.006 | | | | | | |  |
| **Item 17 WURSS-21**  **AUC (12 days)** | **N** | **Mean** | **SD** | **Min** | **Q25** | **Median** | **Q75** | **Max** | **CI** |
| total | 252 | 14.09 | 14.47 | 0.0 | 3.00 | 9.50 | 21.00 | 67.5 | 12.2 … 15.9 |
| V group | 127 | 11.54 | 12.73 | 0.0 | 2.00 | 6.00 | 17.50 | 61.0 | 9.2 … 13.8 |
| P group | 125 | 16.69 | 15.66 | 0.0 | 4.00 | 13.00 | 25.00 | 67.5 | 13.9 … 19.5 |
| p_U_ value | | 0.004 | | | | | | |  |

| **Item 18 WURSS-21**  **AUC (12 days)** | **N** | **Mean** | **SD** | **Min** | **Q25** | **Median** | **Q75** | **Max** | **CI** |
| --- | --- | --- | --- | --- | --- | --- | --- | --- | --- |
| total | 252 | 13.04 | 13.88 | 0.0 | 2.00 | 9.00 | 20.00 | 65.5 | 11.3 … 14.8 |
| V group | 127 | 10.75 | 12.15 | 0.0 | 2.00 | 7.00 | 15.00 | 59.0 | 8.6 … 12.9 |
| P group | 125 | 15.36 | 15.14 | 0.0 | 3.00 | 12.00 | 24.00 | 65.5 | 12.6 … 18.1 |
| p_U_ value | | 0.007 | | | | | | |  |

| **Item 19 WURSS-21**  **AUC (12 days)** | **N** | **Mean** | **SD** | **Min** | **Q25** | **Median** | **Q75** | **Max** | **CI** |
| --- | --- | --- | --- | --- | --- | --- | --- | --- | --- |
| total | 252 | 12.83 | 13.97 | 0.0 | 2.00 | 8.50 | 18.00 | 64.5 | 11.0 … 14.6 |
| V group | 127 | 10.53 | 12.20 | 0.0 | 1.00 | 7.00 | 14.00 | 59.0 | 8.3 … 12.7 |
| P group | 125 | 15.16 | 15.27 | 0.0 | 3.00 | 11.00 | 24.00 | 64.5 | 12.4 … 17.9 |
| p_U_ value | | 0.012 | | | | | | |  |

| **Item 20 WURSS-21**  **AUC (12 days)** | **N** | **Mean** | **SD** | **Min** | **Q25** | **Median** | **Q75** | **Max** | **CI** |
| --- | --- | --- | --- | --- | --- | --- | --- | --- | --- |
| total | 252 | 12.24 | 13.85 | 0.0 | 2.00 | 8.00 | 17.50 | 65.5 | 10.5 … 14.0 |
| V group | 127 | 10.20 | 12.18 | 0.0 | 2.00 | 6.00 | 13.50 | 60.0 | 8.0 … 12.4 |
| P group | 125 | 14.32 | 15.13 | 0.0 | 3.00 | 9.00 | 23.00 | 65.5 | 11.6 … 17.0 |
| p_U_ value | | 0.024 | | | | | | |  |

**Fig 3: Mean daily sum score over 12 days based on morning and evening values for Jackson score and evening values for the 9-item WURSS-21 QoL domain**

References: Table 122 in CI30-018 and Table 170 in CI30-018.

| **Day** | **V group** | | | **P group** | | | **p_U_ – value** |
| --- | --- | --- | --- | --- | --- | --- | --- |
|  | N | Mean (SD) | Min … Max (Median) | N | Mean (SD) | Min … Max (Median) |  |
| 1 morning | 129 | 7.59 (4.68) | 0 … 20 (7.0) | 127 | 7.46 (5.34) | 0 … 21 (7.0) | 0.654 |
| 1 evening | 136 | 8.74 (4.43) | 1 … 20 (8.0) | 131 | 8.96 (5.00) | 1 … 23 (8.0) | 0.886 |
| 2 morning | 136 | 9.42 (4.52) | 1 … 21 (9.0) | 131 | 9.76 (5.14) | 0 … 24 (9.0) | 0.729 |
| 2 evening | 136 | 9.42 (4.79) | 0 … 24 (9.0) | 131 | 10.30 (5.18) | 0 … 24 (10.0) | 0.176 |
| 3 morning | 136 | 8.29 (5.07) | 0 … 22 (7.0) | 131 | 9.59 (5.31) | 0 … 22 (9.0) | **0.035** |
| 3 evening | 136 | 7.96 (5.15) | 0 … 22 (8.0) | 131 | 9.32 (5.49) | 0 … 23 (9.0) | **0.045** |
| 4 morning | 136 | 6.57 (5.45) | 0 … 23 (6.0) | 131 | 7.86 (5.48) | 0 … 20 (7.0) | **0.038** |
| 4 evening | 136 | 6.05 (5.34) | 0 … 24 (5.0) | 131 | 7.83 (5.65) | 0 … 20 (7.0) | **0.008** |
| 5 morning | 136 | 4.43 (4.66) | 0 … 20 (3.0) | 131 | 6.34 (5.51) | 0 … 20 (5.0) | **0.004** |
| 5 evening | 136 | 4.29 (4.82) | 0 … 23 (3.0) | 131 | 5.89 (5.58) | 0 … 22 (5.0) | **0.019** |
| 6 morning | 136 | 3.42 (4.41) | 0 … 19 (2.0) | 131 | 4.43 (4.80) | 0 … 21 (3.0) | 0.056 |
| 6 evening | 136 | 3.05 (4.09) | 0 … 19 (1.5) | 131 | 4.01 (4.85) | 0 … 23 (2.0) | 0.101 |
| 7 morning | 136 | 2.62 (4.02) | 0 … 16 (0.0) | 131 | 3.08 (4.18) | 0 … 22 (1.0) | 0.197 |
| 7 evening | 136 | 2.43 (4.05) | 0 … 16 (0.0) | 131 | 2.91 (4.31) | 0 … 22 (1.0) | 0.113 |
| 8 morning | 136 | 1.85 (3.58) | 0 … 18 (0.0) | 131 | 2.12 (3.54) | 0 … 18 (0.0) | 0.348 |
| 8 evening | 136 | 1.69 (3.39) | 0 … 18 (0.0) | 131 | 2.04 (3.55) | 0 … 19 (0.0) | 0.297 |
| 9 morning | 136 | 1.27 (2.72) | 0 … 15 (0.0) | 131 | 1.74 (3.15) | 0 … 15 (0.0) | 0.121 |
| 9 evening | 136 | 1.25 (2.82) | 0 … 15 (0.0) | 131 | 1.73 (3.25) | 0 … 17 (0.0) | 0.140 |
| 10 morning | 136 | 0.93 (2.47) | 0 … 13 (0.0) | 131 | 1.31 (2.83) | 0 … 16 (0.0) | 0.067 |
| 10 evening | 136 | 0.91 (2.50) | 0 … 12 (0.0) | 131 | 1.32 (3.14) | 0 … 20 (0.0) | **0.040** |
| 11 morning | 136 | 0.68 (2.04) | 0 … 12 (0.0) | 131 | 1.12 (3.02) | 0 … 20 (0.0) | **0.033** |
| 11 evening | 136 | 0.63 (1.92) | 0 … 11 (0.0) | 131 | 1.10 (3.16) | 0 … 22 (0.0) | **0.023** |
| 12 morning | 136 | 0.46 (1.43) | 0 … 8 (0.0) | 131 | 0.89 (2.84) | 0 … 21 (0.0) | 0.116 |
| 12 evening | 136 | 0.39 (1.35) | 0 … 9 (0.0) | 131 | 0.82 (2.76) | 0 … 20 (0.0) | 0.102 |
| 13 morning | 136 | 0.27 (1.05) | 0 … 7 (0.0) | 131 | 0.71 (2.61) | 0 … 17 (0.0) | 0.424 |
| 13 evening | 136 | 0.22 (0.86) | 0 … 6 (0.0) | 131 | 0.66 (2.49) | 0 … 16 (0.0) | 0.312 |
| 14 morning | 136 | 0.18 (0.86) | 0 … 6 (0.0) | 131 | 0.56 (2.13) | 0 … 16 (0.0) | 0.060 |
| 14 evening | 136 | 0.16 (0.74) | 0 … 6 (0.0) | 131 | 0.57 (2.32) | 0 … 18 (0.0) | 0.179 |
| 15 morning | 136 | 0.12 (0.67) | 0 … 6 (0.0) | 131 | 0.46 (1.92) | 0 … 14 (0.0) | 0.143 |
| 15 evening | 136 | 0.12 (0.62) | 0 … 5 (0.0) | 131 | 0.53 (2.17) | 0 … 15 (0.0) | 0.135 |
| 16 morning | 136 | 0.06 (0.42) | 0 … 4 (0.0) | 131 | 0.47 (1.92) | 0 … 13 (0.0) | **0.013** |
| 16 evening | 136 | 0.03 (0.27) | 0 … 3 (0.0) | 131 | 0.44 (2.01) | 0 … 14 (0.0) | **0.008** |
| 17 morning | 136 | 0.02 (0.26) | 0 … 3 (0.0) | 131 | 0.19 (0.99) | 0 … 9 (0.0) | **0.024** |
| 17 evening | 136 | 0.00 |  | 131 | 0.19 (0.99) | 0 … 9 (0.0) | **0.006** |
| 18 morning | 136 | 0.00 |  | 131 | 0.15 (0.96) | 0 … 10 (0.0) | **0.013** |
| 18 evening | 136 | 0.00 |  | 131 | 0.08 (0.42) | 0 … 3 (0.0) | **0.027** |
| 19 morning | 136 | 0.00 |  | 131 | 0.06 (0.41) | 0 … 3 (0.0) | 0.117 |
| 19 evening | 136 | 0.00 |  | 131 | 0.02 (0.12) | 0 … 1 (0.0) | 0.240 |
| 20 morning | 136 | 0.00 |  | 131 | 0.00 |  | 1.000 |
| 20 evening | 136 | 0.00 |  | 131 | 0.00 |  | 1.000 |

| **Day** | **V group** | | | **P group** | | | **p_U_ – value** |
| --- | --- | --- | --- | --- | --- | --- | --- |
|  | N | Mean (SD) | Min … Max (Median) | N | Mean (SD) | Min … Max (Median) |  |
| 1 | 127 | 17.68 (13.65) | 0 … 53 (14.0) | 125 | 21.20 (15.93) | 0 … 63 (20.0) | 0.113 |
| 2 | 127 | 20.43 (14.98) | 0 … 57 (19.0) | 125 | 24.66 (16.25) | 0 … 62 (23.0) | **0.038** |
| 3 | 127 | 18.37 (15.44) | 0 … 55 (15.0) | 125 | 24.12 (16.69) | 0 … 63 (23.0) | **0.006** |
| 4 | 127 | 14.52 (15.21) | 0 … 54 (10.0) | 125 | 20.75 (16.67) | 0 … 58 (17.0) | **0.002** |
| 5 | 127 | 11.40 (14.99) | 0 … 63 (3.0) | 125 | 17.01 (17.03) | 0 … 55 (13.0) | **0.007** |
| 6 | 127 | 9.35 (15.20) | 0 … 63 (1.0) | 125 | 13.42 (16.13) | 0 … 63 (9.0) | **0.015** |
| 7 | 127 | 7.08 (13.85) | 0 … 63 (0.0) | 125 | 10.27 (15.73) | 0 … 63 (2.0) | **0.019** |
| 8 | 127 | 5.02 (12.31) | 0 … 63 (0.0) | 125 | 6.86 (13.05) | 0 … 58 (0.0) | 0.068 |
| 9 | 127 | 3.54 (10.08) | 0 … 54 (0.0) | 125 | 5.78 (12.63) | 0 … 63 (0.0) | **0.037** |
| 10 | 127 | 2.90 (9.58) | 0 … 54 (0.0) | 125 | 4.09 (10.84) | 0 … 57 (0.0) | **0.018** |
| 11 | 127 | 2.68 (9.97) | 0 … 55 (0.0) | 125 | 3.35 (10.56) | 0 … 63 (0.0) | **0.034** |
| 12 | 127 | 1.18 (5.35) | 0 … 36 (0.0) | 125 | 2.36 (8.83) | 0 … 62 (0.0) | **0.037** |
| 13 | 127 | 0.68 (3.70) | 0 … 27 (0.0) | 125 | 2.07 (8.77) | 0 … 61 (0.0) | 0.147 |
| 14 | 127 | 0.60 (3.61) | 0 … 29 (0.0) | 125 | 1.81 (8.16) | 0 … 62 (0.0) | 0.114 |
| 15 | 127 | 0.39 (2.61) | 0 … 27 (0.0) | 125 | 1.50 (7.66) | 0 … 60 (0.0) | 0.197 |
| 16 | 127 | 0.24 (2.42) | 0 … 27 (0.0) | 125 | 1.30 (6.76) | 0 … 54 (0.0) | **0.036** |
| 17 | 127 | 0.21 (2.40) | 0 … 27 (0.0) | 125 | 0.52 (3.55) | 0 … 36 (0.0) | 0.104 |
| 18 | 127 | 0.21 (2.40) | 0 … 27 (0.0) | 125 | 0.14 (1.12) | 0 … 12 (0.0) | 0.368 |
| 19 | 127 | 0.00 |  | 125 | 0.08 (0.74) | 0 … 8 (0.0) | 0.245 |
| 20 | 127 | 0.00 |  | 125 | 0.02 (0.18) | 0 … 2 (0.0) | 0.496 |

**Fig 4: Daily frequency of subjects using rescue medication**

Reference: Table 16 in REP-18031.

| **Subjects**  **with CM** | **V group (N = 136)** | | **P group (N = 131)** | | **p_exF_**  **value** |
| --- | --- | --- | --- | --- | --- |
|  | Number | Percentage | Number | Percentage |  |
| **Day 1** | 24 | 17.6% | 37 | 28.2% | **0.042** |
| **Day 2** | 36 | 26.5% | 46 | 35.1% | 0.145 |
| **Day 3** | 30 | 22.1% | 48 | 36.6% | **0.011** |
| **Day 4** | 30 | 22.1% | 45 | 34.4% | **0.029** |
| **Day 5** | 24 | 17.6% | 33 | 25.2% | 0.139 |
| **Day 6** | 22 | 16.2% | 27 | 20.6% | 0.429 |
| **Day 7** | 17 | 12.5% | 21 | 16.0% | 0.484 |
| **Day 8** | 11 | 8.1% | 13 | 9.9% | 0.671 |
| **Day 9** | 9 | 6.6% | 11 | 8.4% | 0.646 |
| **Day 10** | 8 | 5.9% | 10 | 7.6% | 0.631 |
| **Day 11** | 5 | 3.7% | 10 | 7.6% | 0.190 |
| **Day 12** | 4 | 2.9% | 7 | 5.3% | 0.370 |
| **Day 13** | 3 | 2.2% | 6 | 4.6% | 0.327 |
| **Day 14** | 3 | 2.2% | 5 | 3.8% | 0.494 |
| **Day 15** | 1 | 0.7% | 4 | 3.1% | 0.207 |
| **Day 16** | 1 | 0.7% | 4 | 3.1% | 0.207 |
| **Day 17** | 0 | 0.0% | 0 | 0.0% | 1.000 |
| **Day 18** | 0 | 0.0% | 0 | 0.0% | 1.000 |
| **Day 19** | 0 | 0.0% | 0 | 0.0% | 1.000 |
| **Day 20** | 0 | 0.0% | 0 | 0.0% | 1.000 |

Supporting information

**SA: Jackson score individual items morning and evening day 1-12**

Reference: Tables 58, 66, 74, 82, 90, 98, 106 and 114 in CI30-018.

| **Sore Throat**  **Day** | **V group** | | | **P group** | | | **p_U_ value** |
| --- | --- | --- | --- | --- | --- | --- | --- |
|  | N | Mean (SD) | Min … Max (Median) | N | Mean (SD) | Min … Max (Median) |  |
| 1 morning | 128 | 1.20 (0.85) | 0 … 3 (1.0) | 126 | 1.26 (0.97) | 0 … 3 (1.0) | 0.706 |
| 1 evening | 135 | 1.41 (0.82) | 0 … 3 (1.0) | 131 | 1.49 (0.92) | 0 … 3 (1.0) | 0.505 |
| 2 morning | 136 | 1.60 (0.87) | 0 … 3 (2.0) | 131 | 1.64 (0.99) | 0 … 3 (2.0) | 0.523 |
| 2 evening | 136 | 1.54 (0.87) | 0 … 3 (2.0) | 131 | 1.72 (0.97) | 0 … 3 (2.0) | 0.075 |
| 3 morning | 136 | 1.35 (0.91) | 0 … 3 (1.0) | 131 | 1.63 (1.02) | 0 … 3 (2.0) | **0.015** |
| 3 evening | 136 | 1.32 (0.92) | 0 … 3 (1.0) | 131 | 1.55 (1.02) | 0 … 3 (2.0) | **0.041** |
| 4 morning | 136 | 1.10 (0.93) | 0 … 3 (1.0) | 131 | 1.34 (1.04) | 0 … 3 (1.0) | 0.060 |
| 4 evening | 136 | 0.99 (0.94) | 0 … 3 (1.0) | 131 | 1.34 (1.08) | 0 … 3 (1.0) | **0.007** |
| 5 morning | 136 | 0.74 (0.86) | 0 … 3 (0.5) | 131 | 1.07 (0.99) | 0 … 3 (1.0) | **0.005** |
| 5 evening | 136 | 0.67 (0.83) | 0 … 3 (0.0) | 131 | 0.94 (0.95) | 0 … 3 (1.0) | **0.018** |
| 6 morning | 136 | 0.59 (0.80) | 0 … 3 (0.0) | 131 | 0.72 (0.86) | 0 … 3 (0.0) | 0.204 |
| 6 evening | 136 | 0.52 (0.76) | 0 … 3 (0.0) | 131 | 0.63 (0.86) | 0 … 3 (0.0) | 0.354 |
| 7 morning | 136 | 0.38 (0.67) | 0 … 3 (0.0) | 131 | 0.49 (0.76) | 0 … 3 (0.0) | 0.271 |
| 7 evening | 136 | 0.37 (0.69) | 0 … 3 (0.0) | 131 | 0.47 (0.76) | 0 … 3 (0.0) | 0.185 |
| 8 morning | 136 | 0.24 (0.56) | 0 … 3 (0.0) | 131 | 0.34 (0.65) | 0 … 3 (0.0) | 0.187 |
| 8 evening | 136 | 0.21 (0.49) | 0 … 2 (0.0) | 131 | 0.33 (0.67) | 0 … 3 (0.0) | 0.195 |
| 9 morning | 136 | 0.17 (0.50) | 0 … 3 (0.0) | 131 | 0.26 (0.53) | 0 … 2 (0.0) | 0.066 |
| 9 evening | 136 | 0.15 (0.41) | 0 … 2 (0.0) | 131 | 0.26 (0.56) | 0 … 2 (0.0) | 0.087 |
| 10 morning | 136 | 0.10 (0.32) | 0 … 2 (0.0) | 131 | 0.20 (0.56) | 0 … 3 (0.0) | 0.181 |
| 10 evening | 136 | 0.09 (0.31) | 0 … 2 (0.0) | 131 | 0.20 (0.50) | 0 … 3 (0.0) | **0.046** |
| 11 morning | 136 | 0.07 (0.28) | 0 … 2 (0.0) | 131 | 0.13 (0.42) | 0 … 2 (0.0) | 0.203 |
| 11 evening | 136 | 0.06 (0.24) | 0 … 1 (0.0) | 131 | 0.10 (0.37) | 0 … 2 (0.0) | 0.491 |
| 12 morning | 136 | 0.04 (0.19) | 0 … 1 (0.0) | 131 | 0.08 (0.32) | 0 … 2 (0.0) | 0.309 |
| 12 evening | 136 | 0.04 (0.21) | 0 … 1 (0.0) | 131 | 0.08 (0.34) | 0 … 2 (0.0) | 0.598 |
| 13 morning | 136 | 0.03 (0.17) | 0 … 1 (0.0) | 131 | 0.07 (0.33) | 0 … 2 (0.0) | 0.368 |
| 13 evening | 136 | 0.03 (0.17) | 0 … 1 (0.0) | 131 | 0.07 (0.33) | 0 … 2 (0.0) | 0.368 |
| 14 morning | 136 | 0.01 (0.12) | 0 … 1 (0.0) | 131 | 0.06 (0.27) | 0 … 2 (0.0) | 0.084 |
| 14 evening | 136 | 0.01 (0.09) | 0 … 1 (0.0) | 131 | 0.05 (0.26) | 0 … 2 (0.0) | 0.056 |
| 15 morning | 136 | 0.01 (0.09) | 0 … 1 (0.0) | 131 | 0.05 (0.26) | 0 … 2 (0.0) | 0.056 |
| 15 evening | 136 | 0.01 (0.09) | 0 … 1 (0.0) | 131 | 0.05 (0.26) | 0 … 2 (0.0) | 0.056 |
| 16 morning | 136 | 0.00 |  | 131 | 0.05 (0.21) | 0 … 1 (0.0) | **0.013** |
| 16 evening | 136 | 0.00 |  | 131 | 0.04 (0.19) | 0 … 1 (0.0) | **0.027** |
| 17 morning | 136 | 0.00 |  | 131 | 0.02 (0.15) | 0 … 1 (0.0) | 0.117 |
| 17 evening | 136 | 0.00 |  | 131 | 0.02 (0.15) | 0 … 1 (0.0) | 0.117 |
| 18 morning | 136 | 0.00 |  | 131 | 0.02 (0.15) | 0 … 1 (0.0) | 0.117 |
| 18 evening | 136 | 0.00 |  | 131 | 0.02 (0.12) | 0 … 1 (0.0) | 0.240 |
| 19 morning | 136 | 0.00 |  | 131 | 0.02 (0.12) | 0 … 1 (0.0) | 0.240 |
| 19 evening | 136 | 0.00 |  | 131 | 0.00 |  | 1.000 |
| 20 morning | 136 | 0.00 |  | 131 | 0.00 |  | 1.000 |
| 20 evening | 136 | 0.00 |  | 131 | 0.00 |  | 1.000 |

| **Blocked nose**  **Day** | **V group** | | | **P group** | | | **p_U_ – value** |
| --- | --- | --- | --- | --- | --- | --- | --- |
|  | N | Mean (SD) | Min … Max (Median) | N | Mean (SD) | Min … Max (Median) |  |
| 1 morning | 128 | 1.04 (0.94) | 0 … 3 (1.0) | 123 | 1.02 (1.02) | 0 … 3 (1.0) | 0.763 |
| 1 evening | 133 | 1.20 (0.97) | 0 … 3 (1.0) | 129 | 1.21 (1.06) | 0 … 3 (1.0) | 0.930 |
| 2 morning | 136 | 1.29 (1.05) | 0 … 3 (1.0) | 131 | 1.49 (1.08) | 0 … 3 (2.0) | 0.137 |
| 2 evening | 136 | 1.32 (1.03) | 0 … 3 (1.0) | 131 | 1.57 (1.09) | 0 … 3 (2.0) | **0.045** |
| 3 morning | 136 | 1.18 (1.03) | 0 … 3 (1.0) | 131 | 1.54 (1.05) | 0 … 3 (2.0) | **0.005** |
| 3 evening | 136 | 1.15 (1.04) | 0 … 3 (1.0) | 131 | 1.43 (1.05) | 0 … 3 (1.0) | **0.029** |
| 4 morning | 136 | 0.99 (0.99) | 0 … 3 (1.0) | 131 | 1.25 (1.05) | 0 … 3 (1.0) | **0.041** |
| 4 evening | 136 | 0.92 (0.98) | 0 … 3 (1.0) | 131 | 1.23 (1.04) | 0 … 3 (1.0) | **0.013** |
| 5 morning | 136 | 0.72 (0.84) | 0 … 3 (0.5) | 131 | 0.94 (0.97) | 0 … 3 (1.0) | 0.079 |
| 5 evening | 136 | 0.68 (0.82) | 0 … 3 (0.0) | 131 | 0.87 (0.95) | 0 … 3 (1.0) | 0.137 |
| 6 morning | 136 | 0.56 (0.81) | 0 … 3 (0.0) | 131 | 0.66 (0.86) | 0 … 3 (0.0) | 0.312 |
| 6 evening | 136 | 0.52 (0.78) | 0 … 3 (0.0) | 131 | 0.56 (0.79) | 0 … 3 (0.0) | 0.590 |
| 7 morning | 136 | 0.44 (0.74) | 0 … 3 (0.0) | 131 | 0.50 (0.76) | 0 … 3 (0.0) | 0.488 |
| 7 evening | 136 | 0.40 (0.74) | 0 … 3 (0.0) | 131 | 0.47 (0.77) | 0 … 3 (0.0) | 0.355 |
| 8 morning | 136 | 0.29 (0.61) | 0 … 3 (0.0) | 131 | 0.37 (0.68) | 0 … 3 (0.0) | 0.315 |
| 8 evening | 136 | 0.30 (0.65) | 0 … 3 (0.0) | 131 | 0.31 (0.65) | 0 … 3 (0.0) | 0.923 |
| 9 morning | 136 | 0.24 (0.54) | 0 … 3 (0.0) | 131 | 0.34 (0.62) | 0 … 3 (0.0) | 0.180 |
| 9 evening | 136 | 0.21 (0.50) | 0 … 2 (0.0) | 131 | 0.29 (0.59) | 0 … 3 (0.0) | 0.178 |
| 10 morning | 136 | 0.14 (0.42) | 0 … 2 (0.0) | 131 | 0.25 (0.56) | 0 … 3 (0.0) | 0.053 |
| 10 evening | 136 | 0.14 (0.44) | 0 … 3 (0.0) | 131 | 0.24 (0.57) | 0 … 3 (0.0) | 0.124 |
| 11 morning | 136 | 0.10 (0.32) | 0 … 2 (0.0) | 131 | 0.22 (0.57) | 0 … 3 (0.0) | 0.065 |
| 11 evening | 136 | 0.11 (0.42) | 0 … 3 (0.0) | 131 | 0.20 (0.56) | 0 … 3 (0.0) | 0.141 |
| 12 morning | 136 | 0.08 (0.35) | 0 … 2 (0.0) | 131 | 0.15 (0.53) | 0 … 3 (0.0) | 0.278 |
| 12 evening | 136 | 0.08 (0.35) | 0 … 2 (0.0) | 131 | 0.12 (0.43) | 0 … 2 (0.0) | 0.419 |
| 13 morning | 136 | 0.05 (0.25) | 0 … 2 (0.0) | 131 | 0.13 (0.45) | 0 … 2 (0.0) | 0.145 |
| 13 evening | 136 | 0.04 (0.24) | 0 … 2 (0.0) | 131 | 0.14 (0.46) | 0 … 2 (0.0) | 0.053 |
| 14 morning | 136 | 0.03 (0.21) | 0 … 2 (0.0) | 131 | 0.11 (0.40) | 0 … 2 (0.0) | **0.039** |
| 14 evening | 136 | 0.04 (0.22) | 0 … 2 (0.0) | 131 | 0.11 (0.40) | 0 … 2 (0.0) | 0.080 |
| 15 morning | 136 | 0.02 (0.15) | 0 … 1 (0.0) | 131 | 0.08 (0.34) | 0 … 2 (0.0) | 0.135 |
| 15 evening | 136 | 0.01 (0.12) | 0 … 1 (0.0) | 131 | 0.08 (0.35) | 0 … 2 (0.0) | **0.037** |
| 16 morning | 136 | 0.01 (0.09) | 0 … 1 (0.0) | 131 | 0.08 (0.32) | 0 … 2 (0.0) | **0.014** |
| 16 evening | 136 | 0.00 |  | 131 | 0.06 (0.30) | 0 … 2 (0.0) | **0.013** |
| 17 morning | 136 | 0.00 |  | 131 | 0.02 (0.15) | 0 … 1 (0.0) | 0.117 |
| 17 evening | 136 | 0.00 |  | 131 | 0.02 (0.15) | 0 … 1 (0.0) | 0.117 |
| 18 morning | 136 | 0.00 |  | 131 | 0.02 (0.19) | 0 … 2 (0.0) | 0.240 |
| 18 evening | 136 | 0.00 |  | 131 | 0.01 (0.09) | 0 … 1 (0.0) | 0.491 |
| 19 morning | 136 | 0.00 |  | 131 | 0.01 (0.09) | 0 … 1 (0.0) | 0.491 |
| 19 evening | 136 | 0.00 |  | 131 | 0.01 (0.09) | 0 … 1 (0.0) | 0.491 |
| 20 morning | 136 | 0.00 |  | 131 | 0.00 |  | 1.000 |
| 20 evening | 136 | 0.00 |  | 131 | 0.00 |  | 1.000 |

| **Runny nose**  **Day** | **V group** | | | **P group** | | | **p_U_ value** |
| --- | --- | --- | --- | --- | --- | --- | --- |
|  | N | Mean (SD) | Min … Max (Median) | N | Mean (SD) | Min … Max (Median) |  |
| 1 morning | 127 | 0.92 (0.90) | 0 … 3 (1.0) | 125 | 0.98 (1.03) | 0 … 3 (1.0) | 0.830 |
| 1 evening | 136 | 1.12 (0.99) | 0 … 3 (1.0) | 127 | 1.14 (1.00) | 0 … 3 (1.0) | 0.911 |
| 2 morning | 136 | 1.30 (1.00) | 0 … 3 (1.0) | 131 | 1.32 (1.04) | 0 … 3 (1.0) | 0.918 |
| 2 evening | 136 | 1.29 (1.01) | 0 … 3 (1.0) | 131 | 1.30 (1.04) | 0 … 3 (1.0) | 0.931 |
| 3 morning | 136 | 1.18 (1.01) | 0 … 3 (1.0) | 131 | 1.36 (1.06) | 0 … 3 (1.0) | 0.170 |
| 3 evening | 136 | 1.10 (1.01) | 0 … 3 (1.0) | 131 | 1.31 (1.05) | 0 … 3 (1.0) | 0.088 |
| 4 morning | 136 | 0.96 (0.97) | 0 … 3 (1.0) | 131 | 1.18 (1.01) | 0 … 3 (1.0) | 0.060 |
| 4 evening | 136 | 0.93 (1.00) | 0 … 3 (1.0) | 131 | 1.22 (1.07) | 0 … 3 (1.0) | **0.026** |
| 5 morning | 136 | 0.72 (0.82) | 0 … 3 (1.0) | 131 | 0.92 (0.95) | 0 … 3 (1.0) | 0.101 |
| 5 evening | 136 | 0.71 (0.87) | 0 … 3 (0.0) | 131 | 0.89 (0.96) | 0 … 3 (1.0) | 0.140 |
| 6 morning | 136 | 0.54 (0.77) | 0 … 3 (0.0) | 131 | 0.66 (0.84) | 0 … 3 (0.0) | 0.230 |
| 6 evening | 136 | 0.49 (0.69) | 0 … 3 (0.0) | 131 | 0.60 (0.83) | 0 … 3 (0.0) | 0.462 |
| 7 morning | 136 | 0.43 (0.69) | 0 … 3 (0.0) | 131 | 0.46 (0.75) | 0 … 3 (0.0) | 1.000 |
| 7 evening | 136 | 0.38 (0.70) | 0 … 3 (0.0) | 131 | 0.43 (0.67) | 0 … 3 (0.0) | 0.322 |
| 8 morning | 136 | 0.31 (0.64) | 0 … 3 (0.0) | 131 | 0.33 (0.59) | 0 … 2 (0.0) | 0.528 |
| 8 evening | 136 | 0.28 (0.62) | 0 … 3 (0.0) | 131 | 0.33 (0.59) | 0 … 2 (0.0) | 0.312 |
| 9 morning | 136 | 0.20 (0.54) | 0 … 3 (0.0) | 131 | 0.23 (0.47) | 0 … 2 (0.0) | 0.256 |
| 9 evening | 136 | 0.24 (0.60) | 0 … 3 (0.0) | 131 | 0.27 (0.56) | 0 … 2 (0.0) | 0.267 |
| 10 morning | 136 | 0.18 (0.53) | 0 … 3 (0.0) | 131 | 0.22 (0.52) | 0 … 3 (0.0) | 0.228 |
| 10 evening | 136 | 0.15 (0.50) | 0 … 3 (0.0) | 131 | 0.22 (0.54) | 0 … 3 (0.0) | 0.193 |
| 11 morning | 136 | 0.12 (0.46) | 0 … 3 (0.0) | 131 | 0.16 (0.46) | 0 … 2 (0.0) | 0.302 |
| 11 evening | 136 | 0.10 (0.40) | 0 … 3 (0.0) | 131 | 0.21 (0.56) | 0 … 3 (0.0) | **0.039** |
| 12 morning | 136 | 0.05 (0.22) | 0 … 1 (0.0) | 131 | 0.19 (0.57) | 0 … 3 (0.0) | **0.029** |
| 12 evening | 136 | 0.03 (0.17) | 0 … 1 (0.0) | 131 | 0.17 (0.54) | 0 … 3 (0.0) | **0.007** |
| 13 morning | 136 | 0.03 (0.17) | 0 … 1 (0.0) | 131 | 0.13 (0.49) | 0 … 3 (0.0) | 0.055 |
| 13 evening | 136 | 0.03 (0.17) | 0 … 1 (0.0) | 131 | 0.11 (0.44) | 0 … 2 (0.0) | 0.089 |
| 14 morning | 136 | 0.04 (0.21) | 0 … 1 (0.0) | 131 | 0.09 (0.38) | 0 … 2 (0.0) | 0.425 |
| 14 evening | 136 | 0.04 (0.21) | 0 … 1 (0.0) | 131 | 0.10 (0.39) | 0 … 2 (0.0) | 0.302 |
| 15 morning | 136 | 0.02 (0.15) | 0 … 1 (0.0) | 131 | 0.08 (0.34) | 0 … 2 (0.0) | 0.135 |
| 15 evening | 136 | 0.03 (0.17) | 0 … 1 (0.0) | 131 | 0.07 (0.33) | 0 … 2 (0.0) | 0.368 |
| 16 morning | 136 | 0.01 (0.09) | 0 … 1 (0.0) | 131 | 0.06 (0.30) | 0 … 2 (0.0) | **0.049** |
| 16 evening | 136 | 0.01 (0.12) | 0 … 1 (0.0) | 131 | 0.07 (0.31) | 0 … 2 (0.0) | 0.072 |
| 17 morning | 136 | 0.01 (0.09) | 0 … 1 (0.0) | 131 | 0.03 (0.17) | 0 … 1 (0.0) | 0.207 |
| 17 evening | 136 | 0.00 |  | 131 | 0.03 (0.17) | 0 … 1 (0.0) | 0.057 |
| 18 morning | 136 | 0.00 |  | 131 | 0.02 (0.19) | 0 … 2 (0.0) | 0.240 |
| 18 evening | 136 | 0.00 |  | 131 | 0.01 (0.09) | 0 … 1 (0.0) | 0.491 |
| 19 morning | 136 | 0.00 |  | 131 | 0.01 (0.09) | 0 … 1 (0.0) | 0.491 |
| 19 evening | 136 | 0.00 |  | 131 | 0.00 |  |  |
| 20 morning | 136 | 0.00 |  | 131 | 0.00 |  |  |
| 20 evening | 136 | 0.00 |  | 131 | 0.00 |  |  |

| **Cough**  **Day** | **V group** | | | **P group** | | | **p_U_ value** |
| --- | --- | --- | --- | --- | --- | --- | --- |
|  | N | Mean (SD) | Min … Max (Median) | N | Mean (SD) | Min … Max (Median) |  |
| 1 morning | 126 | 0.97 (0.96) | 0 … 3 (1.0) | 124 | 0.83 (1.03) | 0 … 3 (0.0) | 0.131 |
| 1 evening | 134 | 1.12 (0.98) | 0 … 3 (1.0) | 130 | 0.99 (1.02) | 0 … 3 (1.0) | 0.245 |
| 2 morning | 136 | 1.27 (0.91) | 0 … 3 (1.0) | 131 | 1.18 (1.05) | 0 … 3 (1.0) | 0.405 |
| 2 evening | 136 | 1.32 (0.92) | 0 … 3 (1.0) | 131 | 1.28 (1.06) | 0 … 3 (1.0) | 0.754 |
| 3 morning | 136 | 1.24 (0.97) | 0 … 3 (1.0) | 131 | 1.29 (1.03) | 0 … 3 (1.0) | 0.718 |
| 3 evening | 136 | 1.17 (0.95) | 0 … 3 (1.0) | 131 | 1.31 (1.04) | 0 … 3 (1.0) | 0.264 |
| 4 morning | 136 | 0.99 (1.03) | 0 … 3 (1.0) | 131 | 1.21 (1.06) | 0 … 3 (1.0) | 0.081 |
| 4 evening | 136 | 0.95 (1.02) | 0 … 3 (0.0) | 131 | 1.16 (1.08) | 0 … 3 (1.0) | 0.105 |
| 5 morning | 136 | 0.72 (0.93) | 0 … 3 (0.0) | 131 | 1.08 (1.07) | 0 … 3 (1.0) | **0.004** |
| 5 evening | 136 | 0.72 (0.98) | 0 … 3 (0.0) | 131 | 1.02 (1.06) | 0 … 3 (1.0) | **0.010** |
| 6 morning | 136 | 0.60 (0.91) | 0 … 3 (0.0) | 131 | 0.88 (0.99) | 0 … 3 (1.0) | **0.010** |
| 6 evening | 136 | 0.52 (0.83) | 0 … 3 (0.0) | 131 | 0.84 (0.98) | 0 … 3 (1.0) | **0.003** |
| 7 morning | 136 | 0.50 (0.83) | 0 … 3 (0.0) | 131 | 0.69 (0.90) | 0 … 3 (0.0) | 0.057 |
| 7 evening | 136 | 0.43 (0.80) | 0 … 3 (0.0) | 131 | 0.63 (0.94) | 0 … 3 (0.0) | 0.080 |
| 8 morning | 136 | 0.37 (0.73) | 0 … 3 (0.0) | 131 | 0.42 (0.74) | 0 … 3 (0.0) | 0.431 |
| 8 evening | 136 | 0.34 (0.70) | 0 … 3 (0.0) | 131 | 0.40 (0.76) | 0 … 3 (0.0) | 0.411 |
| 9 morning | 136 | 0.26 (0.59) | 0 … 3 (0.0) | 131 | 0.41 (0.75) | 0 … 3 (0.0) | 0.113 |
| 9 evening | 136 | 0.23 (0.54) | 0 … 3 (0.0) | 131 | 0.41 (0.73) | 0 … 3 (0.0) | **0.029** |
| 10 morning | 136 | 0.19 (0.51) | 0 … 3 (0.0) | 131 | 0.35 (0.71) | 0 … 3 (0.0) | **0.050^17^** |
| 10 evening | 136 | 0.18 (0.50) | 0 … 3 (0.0) | 131 | 0.33 (0.73) | 0 … 3 (0.0) | 0.119 |
| 11 morning | 136 | 0.15 (0.49) | 0 … 3 (0.0) | 131 | 0.30 (0.66) | 0 … 3 (0.0) | **0.021** |
| 11 evening | 136 | 0.12 (0.43) | 0 … 3 (0.0) | 131 | 0.29 (0.68) | 0 … 3 (0.0) | **0.031** |
| 12 morning | 136 | 0.10 (0.37) | 0 … 2 (0.0) | 131 | 0.24 (0.65) | 0 … 3 (0.0) | 0.060 |
| 12 evening | 136 | 0.09 (0.33) | 0 … 2 (0.0) | 131 | 0.25 (0.65) | 0 … 3 (0.0) | 0.**022** |
| 13 morning | 136 | 0.05 (0.22) | 0 … 1 (0.0) | 131 | 0.18 (0.61) | 0 … 3 (0.0) | 0.104 |
| 13 evening | 136 | 0.04 (0.21) | 0 … 1 (0.0) | 131 | 0.16 (0.58) | 0 … 3 (0.0) | 0.133 |
| 14 morning | 136 | 0.04 (0.24) | 0 … 2 (0.0) | 131 | 0.13 (0.49) | 0 … 3 (0.0) | 0.122 |
| 14 evening | 136 | 0.04 (0.24) | 0 … 2 (0.0) | 131 | 0.12 (0.46) | 0 … 3 (0.0) | 0.129 |
| 15 morning | 136 | 0.04 (0.22) | 0 … 2 (0.0) | 131 | 0.11 (0.42) | 0 … 3 (0.0) | 0.081 |
| 15 evening | 136 | 0.04 (0.22) | 0 … 2 (0.0) | 131 | 0.11 (0.42) | 0 … 3 (0.0) | 0.081 |
| 16 morning | 136 | 0.02 (0.19) | 0 … 2 (0.0) | 131 | 0.11 (0.42) | 0 … 3 (0.0) | **0.015** |
| 16 evening | 136 | 0.01 (0.17) | 0 … 2 (0.0) | 131 | 0.11 (0.45) | 0 … 3 (0.0) | **0.008** |
| 17 morning | 136 | 0.01 (0.17) | 0 … 2 (0.0) | 131 | 0.08 (0.34) | 0 … 2 (0.0) | **0.033** |
| 17 evening | 136 | 0.00 |  | 131 | 0.08 (0.34) | 0 … 2 (0.0) | **0.006** |
| 18 morning | 136 | 0.00 |  | 131 | 0.06 (0.30) | 0 … 2 (0.0) | **0.013** |
| 18 evening | 136 | 0.00 |  | 131 | 0.04 (0.23) | 0 … 2 (0.0) | 0.057 |
| 19 morning | 136 | 0.00 |  | 131 | 0.02 (0.19) | 0 … 2 (0.0) | 0.240 |
| 19 evening | 136 | 0.00 |  | 131 | 0.01 (0.09) | 0 … 1 (0.0) | 0.491 |
| 20 morning | 136 | 0.00 |  | 131 | 0.00 |  | 1.000 |
| 20 evening | 136 | 0.00 |  | 131 | 0.00 |  | 1.000 |

| **Sneezing**  **Day** | **V group** | | | **P group** | | | **p_U_ value** |
| --- | --- | --- | --- | --- | --- | --- | --- |
|  | N | Mean (SD) | Min … Max (Median) | N | Mean (SD) | Min … Max (Median) |  |
| 1 morning | 129 | 0.88 (0.78) | 0 … 3 (1.0) | 124 | 0.90 (0.91) | 0 … 3 (1.0) | 0.877 |
| 1 evening | 134 | 1.02 (0.83) | 0 … 3 (1.0) | 128 | 0.97 (0.85) | 0 … 3 (1.0) | 0.620 |
| 2 morning | 136 | 1.02 (0.86) | 0 … 3 (1.0) | 131 | 1.07 (0.93) | 0 … 3 (1.0) | 0.767 |
| 2 evening | 136 | 0.99 (0.87) | 0 … 3 (1.0) | 131 | 1.08 (0.91) | 0 … 3 (1.0) | 0.390 |
| 3 morning | 136 | 0.88 (0.84) | 0 … 3 (1.0) | 131 | 1.02 (0.89) | 0 … 3 (1.0) | 0.204 |
| 3 evening | 136 | 0.86 (0.84) | 0 … 3 (1.0) | 131 | 1.02 (0.93) | 0 … 3 (1.0) | 0.211 |
| 4 morning | 136 | 0.73 (0.85) | 0 … 3 (1.0) | 131 | 0.82 (0.85) | 0 … 3 (1.0) | 0.333 |
| 4 evening | 136 | 0.58 (0.78) | 0 … 3 (0.0) | 131 | 0.76 (0.83) | 0 … 3 (1.0) | 0.060 |
| 5 morning | 136 | 0.42 (0.68) | 0 … 3 (0.0) | 131 | 0.63 (0.80) | 0 … 3 (0.0) | **0.014** |
| 5 evening | 136 | 0.38 (0.67) | 0 … 3 (0.0) | 131 | 0.57 (0.77) | 0 … 3 (0.0) | **0.033** |
| 6 morning | 136 | 0.29 (0.61) | 0 … 2 (0.0) | 131 | 0.39 (0.59) | 0 … 3 (0.0) | **0.044** |
| 6 evening | 136 | 0.26 (0.56) | 0 … 2 (0.0) | 131 | 0.36 (0.63) | 0 … 3 (0.0) | 0.122 |
| 7 morning | 136 | 0.24 (0.51) | 0 … 2 (0.0) | 131 | 0.29 (0.55) | 0 … 2 (0.0) | 0.384 |
| 7 evening | 136 | 0.26 (0.56) | 0 … 2 (0.0) | 131 | 0.24 (0.57) | 0 … 3 (0.0) | 0.776 |
| 8 morning | 136 | 0.20 (0.56) | 0 … 3 (0.0) | 131 | 0.17 (0.47) | 0 … 2 (0.0) | 0.770 |
| 8 evening | 136 | 0.15 (0.44) | 0 … 3 (0.0) | 131 | 0.15 (0.42) | 0 … 2 (0.0) | 0.926 |
| 9 morning | 136 | 0.09 (0.33) | 0 … 2 (0.0) | 131 | 0.14 (0.43) | 0 … 3 (0.0) | 0.266 |
| 9 evening | 136 | 0.12 (0.41) | 0 … 2 (0.0) | 131 | 0.12 (0.39) | 0 … 2 (0.0) | 0.810 |
| 10 morning | 136 | 0.07 (0.33) | 0 … 2 (0.0) | 131 | 0.08 (0.32) | 0 … 2 (0.0) | 0.580 |
| 10 evening | 136 | 0.07 (0.30) | 0 … 2 (0.0) | 131 | 0.10 (0.39) | 0 … 3 (0.0) | 0.408 |
| 11 morning | 136 | 0.04 (0.27) | 0 … 2 (0.0) | 131 | 0.07 (0.35) | 0 … 3 (0.0) | 0.468 |
| 11 evening | 136 | 0.04 (0.21) | 0 … 1 (0.0) | 131 | 0.08 (0.39) | 0 … 3 (0.0) | 0.598 |
| 12 morning | 136 | 0.04 (0.19) | 0 … 1 (0.0) | 131 | 0.06 (0.30) | 0 … 2 (0.0) | 0.602 |
| 12 evening | 136 | 0.01 (0.12) | 0 … 1 (0.0) | 131 | 0.05 (0.24) | 0 … 2 (0.0) | 0.231 |
| 13 morning | 136 | 0.01 (0.12) | 0 … 1 (0.0) | 131 | 0.05 (0.26) | 0 … 2 (0.0) | 0.141 |
| 13 evening | 136 | 0.01 (0.09) | 0 … 1 (0.0) | 131 | 0.05 (0.26) | 0 … 2 (0.0) | 0.056 |
| 14 morning | 136 | 0.02 (0.15) | 0 … 1 (0.0) | 131 | 0.05 (0.21) | 0 … 1 (0.0) | 0.327 |
| 14 evening | 136 | 0.01 (0.09) | 0 … 1 (0.0) | 131 | 0.05 (0.24) | 0 … 2 (0.0) | 0.101 |
| 15 morning | 136 | 0.01 (0.09) | 0 … 1 (0.0) | 131 | 0.05 (0.24) | 0 … 2 (0.0) | 0.101 |
| 15 evening | 136 | 0.01 (0.09) | 0 … 1 (0.0) | 131 | 0.05 (0.24) | 0 … 2 (0.0) | 0.101 |
| 16 morning | 136 | 0.00 |  | 131 | 0.05 (0.24) | 0 … 2 (0.0) | **0.027** |
| 16 evening | 136 | 0.00 |  | 131 | 0.05 (0.24) | 0 … 2 (0.0) | **0.027** |
| 17 morning | 136 | 0.00 |  | 131 | 0.02 (0.17) | 0 … 2 (0.0) | 0.491 |
| 17 evening | 136 | 0.00 |  | 131 | 0.01 (0.09) | 0 … 1 (0.0) | 0.491 |
| 18 morning | 136 | 0.00 |  | 131 | 0.01 (0.09) | 0 … 1 (0.0) | 0.491 |
| 18 evening | 136 | 0.00 |  | 131 | 0.00 |  | 1.000 |
| 19 morning | 136 | 0.00 |  | 131 | 0.00 |  | 1.000 |
| 19 evening | 136 | 0.00 |  | 131 | 0.00 |  | 1.000 |
| 20 morning | 136 | 0.00 |  | 131 | 0.00 |  | 1.000 |
| 20 evening | 136 | 0.00 |  | 131 | 0.00 |  | 1.000 |

| **Headache**  **Day** | **V group** | | | **P group** | | | **p_U_ value** |
| --- | --- | --- | --- | --- | --- | --- | --- |
|  | N | Mean (SD) | Min … Max (Median) | N | Mean (SD) | Min … Max (Median) |  |
| 1 morning | 128 | 1.00 (0.99) | 0 … 3 (1.0) | 127 | 0.94 (0.93) | 0 … 3 (1.0) | 0.737 |
| 1 evening | 136 | 1.05 (1.04) | 0 … 3 (1.0) | 128 | 1.20 (0.96) | 0 … 3 (1.0) | 0.180 |
| 2 morning | 136 | 1.10 (1.04) | 0 … 3 (1.0) | 131 | 1.18 (1.05) | 0 … 3 (1.0) | 0.529 |
| 2 evening | 136 | 1.07 (1.00) | 0 … 3 (1.0) | 131 | 1.21 (1.01) | 0 … 3 (1.0) | 0.283 |
| 3 morning | 136 | 0.97 (0.96) | 0 … 3 (1.0) | 131 | 1.11 (0.95) | 0 … 3 (1.0) | 0.200 |
| 3 evening | 136 | 0.99 (1.02) | 0 … 3 (1.0) | 131 | 1.05 (1.00) | 0 … 3 (1.0) | 0.597 |
| 4 morning | 136 | 0.71 (0.93) | 0 … 3 (0.0) | 131 | 0.86 (0.89) | 0 … 3 (1.0) | 0.069 |
| 4 evening | 136 | 0.67 (0.89) | 0 … 3 (0.0) | 131 | 0.86 (0.92) | 0 … 3 (1.0) | 0.071 |
| 5 morning | 136 | 0.45 (0.75) | 0 … 3 (0.0) | 131 | 0.69 (0.86) | 0 … 3 (0.0) | **0.009** |
| 5 evening | 136 | 0.43 (0.74) | 0 … 3 (0.0) | 131 | 0.69 (0.88) | 0 … 3 (0.0) | **0.007** |
| 6 morning | 136 | 0.36 (0.77) | 0 … 3 (0.0) | 131 | 0.48 (0.74) | 0 … 3 (0.0) | **0.040** |
| 6 evening | 136 | 0.30 (0.69) | 0 … 3 (0.0) | 131 | 0.38 (0.73) | 0 … 3 (0.0) | 0.214 |
| 7 morning | 136 | 0.23 (0.63) | 0 … 3 (0.0) | 131 | 0.27 (0.57) | 0 … 2 (0.0) | 0.160 |
| 7 evening | 136 | 0.24 (0.66) | 0 … 3 (0.0) | 131 | 0.25 (0.61) | 0 … 3 (0.0) | 0.503 |
| 8 morning | 136 | 0.18 (0.56) | 0 … 3 (0.0) | 131 | 0.21 (0.59) | 0 … 3 (0.0) | 0.533 |
| 8 evening | 136 | 0.15 (0.51) | 0 … 3 (0.0) | 131 | 0.22 (0.56) | 0 … 3 (0.0) | 0.131 |
| 9 morning | 136 | 0.14 (0.49) | 0 … 3 (0.0) | 131 | 0.16 (0.49) | 0 … 3 (0.0) | 0.634 |
| 9 evening | 136 | 0.12 (0.46) | 0 … 3 (0.0) | 131 | 0.14 (0.51) | 0 … 3 (0.0) | 0.911 |
| 10 morning | 136 | 0.12 (0.44) | 0 … 3 (0.0) | 131 | 0.07 (0.38) | 0 … 3 (0.0) | 0.189 |
| 10 evening | 136 | 0.11 (0.45) | 0 … 3 (0.0) | 131 | 0.08 (0.43) | 0 … 3 (0.0) | 0.538 |
| 11 morning | 136 | 0.07 (0.38) | 0 … 3 (0.0) | 131 | 0.10 (0.48) | 0 … 3 (0.0) | 0.670 |
| 11 evening | 136 | 0.07 (0.36) | 0 … 3 (0.0) | 131 | 0.08 (0.43) | 0 … 3 (0.0) | 0.974 |
| 12 morning | 136 | 0.07 (0.34) | 0 … 2 (0.0) | 131 | 0.06 (0.35) | 0 … 3 (0.0) | 0.706 |
| 12 evening | 136 | 0.07 (0.37) | 0 … 3 (0.0) | 131 | 0.07 (0.40) | 0 … 3 (0.0) | 0.905 |
| 13 morning | 136 | 0.06 (0.37) | 0 … 2 (0.0) | 131 | 0.06 (0.30) | 0 … 2 (0.0) | 0.327 |
| 13 evening | 136 | 0.03 (0.21) | 0 … 2 (0.0) | 131 | 0.05 (0.34) | 0 … 3 (0.0) | 0.571 |
| 14 morning | 136 | 0.02 (0.19) | 0 … 2 (0.0) | 131 | 0.05 (0.31) | 0 … 3 (0.0) | 0.231 |
| 14 evening | 136 | 0.01 (0.17) | 0 … 2 (0.0) | 131 | 0.05 (0.34) | 0 … 3 (0.0) | 0.177 |
| 15 morning | 136 | 0.01 (0.12) | 0 … 1 (0.0) | 131 | 0.04 (0.23) | 0 … 2 (0.0) | 0.365 |
| 15 evening | 136 | 0.01 (0.12) | 0 … 1 (0.0) | 131 | 0.06 (0.32) | 0 … 3 (0.0) | 0.165 |
| 16 morning | 136 | 0.01 (0.12) | 0 … 1 (0.0) | 131 | 0.05 (0.31) | 0 … 3 (0.0) | 0.231 |
| 16 evening | 136 | 0.00 |  | 131 | 0.06 (0.37) | 0 … 3 (0.0) | 0.057 |
| 17 morning | 136 | 0.00 |  | 131 | 0.02 (0.12) | 0 … 1 (0.0) | 0.240 |
| 17 evening | 136 | 0.00 |  | 131 | 0.02 (0.19) | 0 … 2 (0.0) | 0.240 |
| 18 morning | 136 | 0.00 |  | 131 | 0.01 (0.09) | 0 … 1 (0.0) | 0.491 |
| 18 evening | 136 | 0.00 |  | 131 | 0.01 (0.09) | 0 … 1 (0.0) | 0.491 |
| 19 morning | 136 | 0.00 |  | 131 | 0.01 (0.09) | 0 … 1 (0.0) | 0.491 |
| 19 evening | 136 | 0.00 |  | 131 | 0.00 |  | 1.000 |
| 20 morning | 136 | 0.00 |  | 131 | 0.00 |  | 1.000 |
| 20 evening | 136 | 0.00 |  | 131 | 0.00 |  | 1.000 |

| **Malaise**  **Day** | **V group** | | | **P group** | | | **p_U_ value** |
| --- | --- | --- | --- | --- | --- | --- | --- |
|  | N | Mean (SD) | Min … Max (Median) | N | Mean (SD) | Min … Max (Median) |  |
| 1 morning | 127 | 0.97 (0.99) | 0 … 3 (1.0) | 124 | 0.94 (1.03) | 0 … 3 (1.0) | 0.758 |
| 1 evening | 134 | 1.10 (0.96) | 0 … 3 (1.0) | 130 | 1.15 (1.06) | 0 … 3 (1.0) | 0.782 |
| 2 morning | 136 | 1.18 (1.01) | 0 … 3 (1.0) | 131 | 1.18 (1.01) | 0 … 3 (1.0) | 0.998 |
| 2 evening | 136 | 1.17 (1.02) | 0 … 3 (1.0) | 131 | 1.24 (1.02) | 0 … 3 (1.0) | 0.530 |
| 3 morning | 136 | 1.01 (0.97) | 0 … 3 (1.0) | 131 | 1.11 (1.01) | 0 … 3 (1.0) | 0.459 |
| 3 evening | 136 | 0.90 (0.97) | 0 … 3 (1.0) | 131 | 1.08 (1.02) | 0 … 3 (1.0) | 0.143 |
| 4 morning | 136 | 0.76 (0.88) | 0 … 3 (1.0) | 131 | 0.86 (0.97) | 0 … 3 (1.0) | 0.522 |
| 4 evening | 136 | 0.68 (0.87) | 0 … 3 (0.0) | 131 | 0.88 (0.98) | 0 … 3 (1.0) | 0.113 |
| 5 morning | 136 | 0.48 (0.70) | 0 … 3 (0.0) | 131 | 0.73 (0.88) | 0 … 3 (0.0) | **0.022** |
| 5 evening | 136 | 0.43 (0.74) | 0 … 3 (0.0) | 131 | 0.63 (0.90) | 0 … 3 (0.0) | 0.068 |
| 6 morning | 136 | 0.34 (0.67) | 0 … 3 (0.0) | 131 | 0.48 (0.77) | 0 … 3 (0.0) | 0.089 |
| 6 evening | 136 | 0.30 (0.64) | 0 … 3 (0.0) | 131 | 0.47 (0.81) | 0 … 3 (0.0) | 0.107 |
| 7 morning | 136 | 0.26 (0.64) | 0 … 3 (0.0) | 131 | 0.31 (0.67) | 0 … 3 (0.0) | 0.491 |
| 7 evening | 136 | 0.24 (0.61) | 0 … 3 (0.0) | 131 | 0.30 (0.66) | 0 … 3 (0.0) | 0.309 |
| 8 morning | 136 | 0.17 (0.55) | 0 … 3 (0.0) | 131 | 0.24 (0.58) | 0 … 3 (0.0) | 0.146 |
| 8 evening | 136 | 0.14 (0.50) | 0 … 3 (0.0) | 131 | 0.21 (0.55) | 0 … 3 (0.0) | 0.122 |
| 9 morning | 136 | 0.11 (0.42) | 0 … 3 (0.0) | 131 | 0.16 (0.51) | 0 … 3 (0.0) | 0.446 |
| 9 evening | 136 | 0.13 (0.50) | 0 … 3 (0.0) | 131 | 0.18 (0.55) | 0 … 3 (0.0) | 0.289 |
| 10 morning | 136 | 0.10 (0.43) | 0 … 3 (0.0) | 131 | 0.11 (0.44) | 0 … 3 (0.0) | 0.756 |
| 10 evening | 136 | 0.12 (0.47) | 0 … 3 (0.0) | 131 | 0.11 (0.47) | 0 … 3 (0.0) | 0.891 |
| 11 morning | 136 | 0.10 (0.40) | 0 … 3 (0.0) | 131 | 0.10 (0.46) | 0 … 3 (0.0) | 0.779 |
| 11 evening | 136 | 0.09 (0.39) | 0 … 3 (0.0) | 131 | 0.09 (0.44) | 0 … 3 (0.0) | 0.949 |
| 12 morning | 136 | 0.06 (0.27) | 0 … 2 (0.0) | 131 | 0.07 (0.38) | 0 … 3 (0.0) | 0.768 |
| 12 evening | 136 | 0.05 (0.25) | 0 … 2 (0.0) | 131 | 0.07 (0.41) | 0 … 3 (0.0) | 0.749 |
| 13 morning | 136 | 0.04 (0.22) | 0 … 2 (0.0) | 131 | 0.05 (0.31) | 0 … 2 (0.0) | 0.790 |
| 13 evening | 136 | 0.02 (0.15) | 0 … 1 (0.0) | 131 | 0.05 (0.31) | 0 … 2 (0.0) | 0.478 |
| 14 morning | 136 | 0.01 (0.09) | 0 ... 1 (0.0) | 131 | 0.05 (0.34) | 0 … 3 (0.0) | 0.148 |
| 14 evening | 136 | 0.01 (0.09) | 0 ... 1 (0.0) | 131 | 0.06 (0.35) | 0 … 3 (0.0) | 0.086 |
| 15 morning | 136 | 0.01 (0.09) | 0 … 1 (0.0) | 131 | 0.05 (0.24) | 0 … 2 (0.0) | 0.101 |
| 15 evening | 136 | 0.00 |  | 131 | 0.07 (0.33) | 0 … 2 (0.0) | **0.013** |
| 16 morning | 136 | 0.01 (0.09) | 0 ... 1 (0.0) | 131 | 0.06 (0.35) | 0 … 3 (0.0) | 0.086 |
| 16 evening | 136 | 0.00 |  | 131 | 0.05 (0.27) | 0 … 2 (0.0) | 0.057 |
| 17 morning | 136 | 0.00 |  | 131 | 0.01 (0.09) | 0 ... 1 (0.0) | 0.491 |
| 17 evening | 136 | 0.00 |  | 131 | 0.01 (0.09) | 0 ... 1 (0.0) | 0.491 |
| 18 morning | 136 | 0.00 |  | 131 | 0.01 (0.09) | 0 ... 1 (0.0) | 0.491 |
| 18 evening | 136 | 0.00 |  | 131 | 0.00 |  | 1.000 |
| 19 morning | 136 | 0.00 |  | 131 | 0.00 |  | 1.000 |
| 19 evening | 136 | 0.00 |  | 131 | 0.00 |  | 1.000 |
| 20 morning | 136 | 0.00 |  | 131 | 0.00 |  | 1.000 |
| 20 evening | 136 | 0.00 |  | 131 | 0.00 |  | 1.000 |

| **Chilliness**  **Day** | **V group** | | | **P group** | | | **p_U_ value** |
| --- | --- | --- | --- | --- | --- | --- | --- |
|  | N | Mean (SD) | Min … Max (Median) | N | Mean (SD) | Min … Max (Median) |  |
| 1 morning | 127 | 0.60 (0.87) | 0 … 3 (0.0) | 126 | 0.52 (0.86) | 0 … 3 (0.0) | 0.310 |
| 1 evening | 132 | 0.68 (0.89) | 0 … 3 (0.0) | 130 | 0.75 (0.97) | 0 … 3 (0.0) | 0.697 |
| 2 morning | 136 | 0.63 (0.84) | 0 … 3 (0.0) | 131 | 0.63 (0.96) | 0 … 3 (0.0) | 0.583 |
| 2 evening | 136 | 0.71 (0.89) | 0 … 3 (0.0) | 131 | 0.80 (1.05) | 0 … 3 (0.0) | 0.788 |
| 3 morning | 136 | 0.45 (0.79) | 0 … 3 (0.0) | 131 | 0.50 (0.82) | 0 … 3 (0.0) | 0.541 |
| 3 evening | 136 | 0.43 (0.77) | 0 … 3 (0.0) | 131 | 0.55 (0.87) | 0 … 3 (0.0) | 0.288 |
| 4 morning | 136 | 0.30 (0.72) | 0 … 3 (0.0) | 131 | 0.32 (0.62) | 0 … 2 (0.0) | 0.320 |
| 4 evening | 136 | 0.31 (0.73) | 0 … 3 (0.0) | 131 | 0.34 (0.68) | 0 … 3 (0.0) | 0.362 |
| 5 morning | 136 | 0.19 (0.55) | 0 … 3 (0.0) | 131 | 0.27 (0.59) | 0 … 3 (0.0) | 0.129 |
| 5 evening | 136 | 0.24 (0.67) | 0 … 3 (0.0) | 131 | 0.27 (0.63) | 0 … 3 (0.0) | 0.258 |
| 6 morning | 136 | 0.15 (0.56) | 0 … 3 (0.0) | 131 | 0.15 (0.53) | 0 … 3 (0.0) | 0.646 |
| 6 evening | 136 | 0.13 (0.53) | 0 … 3 (0.0) | 131 | 0.14 (0.54) | 0 … 3 (0.0) | 0.622 |
| 7 morning | 136 | 0.13 (0.56) | 0 … 3 (0.0) | 131 | 0.09 (0.41) | 0 … 3 (0.0) | 0.804 |
| 7 evening | 136 | 0.11 (0.43) | 0 … 2 (0.0) | 131 | 0.11 (0.46) | 0 … 3 (0.0) | 0.933 |
| 8 morning | 136 | 0.10 (0.44) | 0 … 3 (0.0) | 131 | 0.06 (0.27) | 0 … 2 (0.0) | 0.587 |
| 8 evening | 136 | 0.10 (0.46) | 0 … 3 (0.0) | 131 | 0.08 (0.36) | 0 … 2 (0.0) | 0.701 |
| 9 morning | 136 | 0.06 (0.32) | 0 … 2 (0.0) | 131 | 0.05 (0.30) | 0 … 3 (0.0) | 0.837 |
| 9 evening | 136 | 0.05 (0.31) | 0 … 2 (0.0) | 131 | 0.05 (0.24) | 0 … 2 (0.0) | 0.744 |
| 10 morning | 136 | 0.04 (0.26) | 0 … 2 (0.0) | 131 | 0.03 (0.17) | 0 … 1 (0.0) | 0.718 |
| 10 evening | 136 | 0.05 (0.31) | 0 … 2 (0.0) | 131 | 0.05 (0.27) | 0 … 2 (0.0) | 0.980 |
| 11 morning | 136 | 0.03 (0.17) | 0 … 1 (0.0) | 131 | 0.05 (0.30) | 0 … 3 (0.0) | 0.861 |
| 11 evening | 136 | 0.04 (0.22) | 0 … 2 (0.0) | 131 | 0.05 (0.32) | 0 … 3 (0.0) | 0.992 |
| 12 morning | 136 | 0.01 (0.12) | 0 … 1 (0.0) | 131 | 0.03 (0.21) | 0 … 2 (0.0) | 0.556 |
| 12 evening | 136 | 0.01 (0.12) | 0 … 1 (0.0) | 131 | 0.02 (0.19) | 0 … 2 (0.0) | 0.811 |
| 13 morning | 136 | 0.01 (0.12) | 0 … 1 (0.0) | 131 | 0.03 (0.21) | 0 … 2 (0.0) | 0.556 |
| 13 evening | 136 | 0.01 (0.12) | 0 … 1 (0.0) | 131 | 0.02 (0.19) | 0 … 2 (0.0) | 0.811 |
| 14 morning | 136 | 0.00 |  | 131 | 0.02 (0.12) | 0 … 1 (0.0) | 0.240 |
| 14 evening | 136 | 0.00 |  | 131 | 0.03 (0.28) | 0 … 3 (0.0) | 0.240 |
| 15 morning | 136 | 0.01 (0.09) | 0 … 1 (0.0) | 131 | 0.02 (0.12) | 0 … 1 (0.0) | 0.617 |
| 15 evening | 136 | 0.01 (0.09) | 0 … 1 (0.0) | 131 | 0.04 (0.23) | 0 … 2 (0.0) | 0.177 |
| 16 morning | 136 | 0.00 |  | 131 | 0.02 (0.15) | 0 … 1 (0.0) | 0.177 |
| 16 evening | 136 | 0.00 |  | 131 | 0.02 (0.12) | 0 … 1 (0.0) | 0.240 |
| 17 morning | 136 | 0.00 |  | 131 | 0.00 |  | 1.000 |
| 17 evening | 136 | 0.00 |  | 131 | 0.00 |  | 1.000 |
| 18 morning | 136 | 0.00 |  | 131 | 0.00 |  | 1.000 |
| 18 evening | 136 | 0.00 |  | 131 | 0.00 |  | 1.000 |
| 19 morning | 136 | 0.00 |  | 131 | 0.00 |  | 1.000 |
| 19 evening | 136 | 0.00 |  | 131 | 0.00 |  | 1.000 |
| 20 morning | 136 | 0.00 |  | 131 | 0.00 |  | 1.000 |
| 20 evening | 136 | 0.00 |  | 131 | 0.00 |  | 1.000 |

**SB:** **9-item WURSS-21 QoL domain individual items: Daily mean score day 1-12**

Reference: Tables 153, 164, 175, 186, 197, 208, 219, 230, 241 in CI30-019.

| **Item 12**  **Day** | **V group** | | | **P group** | | | **p_U_ – value** |
| --- | --- | --- | --- | --- | --- | --- | --- |
|  | N | Mean (SD) | Min … Max (Median) | N | Mean (SD) | Min … Max (Median) |  |
| 1 | 127 | 1.25 (1.62) | 0 … 5 (1.0) | 125 | 1.80 (2.00) | 0 … 7 (1.0) | 0.055 |
| 2 | 127 | 1.88 (2.06) | 0 … 7 (1.0) | 125 | 2.20 (1.97) | 0 … 7 (2.0) | 0.113 |
| 3 | 127 | 1.67 (2.04) | 0 … 7 (1.0) | 125 | 2.22 (2.06) | 0 … 7 (2.0) | **0.014** |
| 4 | 127 | 1.43 (1.95) | 0 … 7 (0.0) | 125 | 1.92 (1.95) | 0 … 6 (1.0) | **0.012** |
| 5 | 127 | 1.10 (1.86) | 0 … 7 (0.0) | 125 | 1.59 (1.88) | 0 … 6 (1.0) | **0.002** |
| 6 | 127 | 0.94 (1.82) | 0 … 7 (0.0) | 125 | 1.14 (1.67) | 0 … 7 (0.0) | **0.018** |
| 7 | 127 | 0.75 (1.69) | 0 … 7 (0.0) | 125 | 0.84 (1.62) | 0 … 7 (0.0) | 0.147 |
| 8 | 127 | 0.55 (1.52) | 0 … 7 (0.0) | 125 | 0.60 (1.30) | 0 … 6 (0.0) | 0.098 |
| 9 | 127 | 0.34 (1.12) | 0 … 6 (0.0) | 125 | 0.45 (1.20) | 0 … 7 (0.0) | 0.099 |
| 10 | 127 | 0.33 (1.20) | 0 … 7 (0.0) | 125 | 0.27 (0.92) | 0 … 6 (0.0) | 0.509 |
| 11 | 127 | 0.32 (1.18) | 0 … 7 (0.0) | 125 | 0.23 (0.98) | 0 … 7 (0.0) | 0.909 |
| 12 | 127 | 0.13 (0.62) | 0 … 4 (0.0) | 125 | 0.18 (0.83) | 0 … 6 (0.0) | 0.549 |
| 13 | 127 | 0.06 (0.39) | 0 … 3 (0.0) | 125 | 0.16 (0.85) | 0 … 6 (0.0) | 0.278 |
| 14 | 127 | 0.06 (0.38) | 0 … 3 (0.0) | 125 | 0.16 (0.80) | 0 … 6 (0.0) | 0.106 |
| 15 | 127 | 0.04 (0.29) | 0 … 3 (0.0) | 125 | 0.12 (0.73) | 0 … 5 (0.0) | 0.478 |
| 16 | 127 | 0.02 (0.27) | 0 … 3 (0.0) | 125 | 0.11 (0.64) | 0 … 5 (0.0) | 0.089 |
| 17 | 127 | 0.02 (0.27) | 0 … 3 (0.0) | 125 | 0.04 (0.37) | 0 … 4 (0.0) | 0.496 |
| 18 | 127 | 0.02 (0.27) | 0 … 3 (0.0) | 125 | 0.00 |  | 1.000 |
| 19 | 127 | 0.00 |  | 125 | 0.00 |  | 1.000 |
| 20 | 127 | 0.00 |  | 125 | 0.00 |  | 1.000 |

| **Item 13**  **Day** | **V group** | | | **P group** | | | **p_U_ – value** |
| --- | --- | --- | --- | --- | --- | --- | --- |
|  | N | Mean (SD) | Min … Max (Median) | N | Mean (SD) | Min … Max (Median) |  |
| 1 | 127 | 2.95 (2.28) | 0 … 7 (3.0) | 125 | 3.12 (2.36) | 0 … 7 (3.0) | 0.613 |
| 2 | 127 | 3.17 (2.16) | 0 … 7 (3.0) | 125 | 3.46 (2.07) | 0 … 7 (3.0) | 0.291 |
| 3 | 127 | 2.67 (2.12) | 0 … 7 (3.0) | 125 | 3.16 (2.07) | 0 … 7 (3.0) | 0.057 |
| 4 | 127 | 2.28 (2.15) | 0 … 7 (2.0) | 125 | 2.86 (2.09) | 0 … 7 (3.0) | **0.027** |
| 5 | 127 | 1.68 (2.04) | 0 … 7 (1.0) | 125 | 2.30 (2.17) | 0 … 7 (2.0) | **0.011** |
| 6 | 127 | 1.38 (1.96) | 0 … 7 (0.0) | 125 | 1.86 (2.01) | 0 … 7 (1.0) | **0.015** |
| 7 | 127 | 1.06 (1.81) | 0 … 7 (0.0) | 125 | 1.45 (1.97) | 0 … 7 (1.0) | **0.020** |
| 8 | 127 | 0.75 (1.60) | 0 … 7 (0.0) | 125 | 0.98 (1.59) | 0 … 6 (0.0) | 0.056 |
| 9 | 127 | 0.50 (1.24) | 0 … 6 (0.0) | 125 | 0.81 (1.50) | 0 … 7 (0.0) | **0.036** |
| 10 | 127 | 0.42 (1.25) | 0 … 7 (0.0) | 125 | 0.59 (1.39) | 0 … 7 (0.0) | 0.081 |
| 11 | 127 | 0.35 (1.22) | 0 … 7 (0.0) | 125 | 0.50 (1.30) | 0 … 7 (0.0) | 0.061 |
| 12 | 127 | 0.17 (0.65) | 0 … 4 (0.0) | 125 | 0.34 (1.07) | 0 … 7 (0.0) | 0.087 |
| 13 | 127 | 0.10 (0.45) | 0 … 3 (0.0) | 125 | 0.28 (1.02) | 0 … 7 (0.0) | 0.132 |
| 14 | 127 | 0.10 (0.59) | 0 … 5 (0.0) | 125 | 0.25 (0.94) | 0 … 7 (0.0) | **0.046** |
| 15 | 127 | 0.07 (0.46) | 0 … 4 (0.0) | 125 | 0.20 (0.97) | 0 … 7 (0.0) | 0.245 |
| 16 | 127 | 0.06 (0.44) | 0 … 4 (0.0) | 125 | 0.15 (0.82) | 0 … 7 (0.0) | 0.234 |
| 17 | 127 | 0.02 (0.27) | 0 … 3 (0.0) | 125 | 0.08 (0.49) | 0 … 4 (0.0) | 0.181 |
| 18 | 127 | 0.02 (0.27) | 0 … 3 (0.0) | 125 | 0.03 (0.28) | 0 … 3 (0.0) | 0.621 |
| 19 | 127 | 0.00 |  | 125 | 0.02 (0.20) | 0 … 2 (0.0) | 0.245 |
| 20 | 127 | 0.00 |  | 125 | 0.01 (0.09) | 0 … 1 (0.0) | 0.496 |

| **Item 14**  **Day** | **V group** | | | **P group** | | | **p_U_ – value** |
| --- | --- | --- | --- | --- | --- | --- | --- |
|  | N | Mean (SD) | Min … Max (Median) | N | Mean (SD) | Min … Max (Median) |  |
| 1 | 127 | 3.20 (2.12) | 0 … 7 (3.0) | 125 | 3.27 (2.20) | 0 … 7 (3.0) | 0.818 |
| 2 | 127 | 3.26 (2.16) | 0 … 7 (3.0) | 125 | 3.53 (2.09) | 0 … 7 (4.0) | 0.330 |
| 3 | 127 | 2.73 (2.17) | 0 … 7 (3.0) | 125 | 3.40 (2.08) | 0 … 7 (4.0) | **0.015** |
| 4 | 127 | 2.09 (2.05) | 0 … 7 (2.0) | 125 | 2.90 (2.15) | 0 … 7 (3.0) | **0.003** |
| 5 | 127 | 1.63 (1.88) | 0 … 7 (1.0) | 125 | 2.34 (2.18) | 0 … 7 (2.0) | **0.008** |
| 6 | 127 | 1.46 (1.88) | 0 … 7 (1.0) | 125 | 1.92 (2.05) | 0 … 7 (1.0) | 0.057 |
| 7 | 127 | 1.04 (1.70) | 0 … 7 (0.0) | 125 | 1.55 (1.97) | 0 … 7 (1.0) | **0.013** |
| 8 | 127 | 0.75 (1.50) | 0 … 7 (0.0) | 125 | 1.04 (1.67) | 0 … 6 (0.0) | 0.141 |
| 9 | 127 | 0.48 (1.17) | 0 … 6 (0.0) | 125 | 0.86 (1.61) | 0 … 7 (0.0) | 0.051 |
| 10 | 127 | 0.37 (1.13) | 0 … 6 (0.0) | 125 | 0.59 (1.32) | 0 … 6 (0.0) | 0.051 |
| 11 | 127 | 0.32 (1.11) | 0 … 7 (0.0) | 125 | 0.47 (1.27) | 0 … 7 (0.0) | 0.079 |
| 12 | 127 | 0.17 (0.64) | 0 … 4 (0.0) | 125 | 0.34 (1.10) | 0 … 7 (0.0) | 0.174 |
| 13 | 127 | 0.09 (0.44) | 0 … 3 (0.0) | 125 | 0.27 (0.99) | 0 … 7 (0.0) | 0.161 |
| 14 | 127 | 0.09 (0.49) | 0 … 3 (0.0) | 125 | 0.22 (0.92) | 0 … 7 (0.0) | 0.117 |
| 15 | 127 | 0.05 (0.30) | 0 … 3 (0.0) | 125 | 0.19 (0.85) | 0 … 7 (0.0) | 0.100 |
| 16 | 127 | 0.02 (0.27) | 0 … 3 (0.0) | 125 | 0.17 (0.80) | 0 … 6 (0.0) | **0.025** |
| 17 | 127 | 0.02 (0.27) | 0 … 3 (0.0) | 125 | 0.07 (0.46) | 0 … 4 (0.0) | 0.181 |
| 18 | 127 | 0.02 (0.27) | 0 … 3 (0.0) | 125 | 0.03 (0.28) | 0 … 1 (0.0) | 0.621 |
| 19 | 127 | 0.00 |  | 125 | 0.02 (0.18) | 0 … 1 (0.0) | 0.496 |
| 20 | 127 | 0.00 |  | 125 | 0.00 |  | 1.000 |

| **Item 15**  **Day** | **V group** | | | **P group** | | | **p_U_ – value** |
| --- | --- | --- | --- | --- | --- | --- | --- |
|  | N | Mean (SD) | Min … Max (Median) | N | Mean (SD) | Min … Max (Median) |  |
| 1 | 127 | 1.71 (1.92) | 0 … 7 (1.0) | 125 | 2.38 (2.15) | 0 … 7 (2.0) | **0.015** |
| 2 | 127 | 2.13 (1.99) | 0 … 7 (1.0) | 125 | 2.78 (2.15) | 0 … 7 (3.0) | **0.017** |
| 3 | 127 | 1.95 (1.97) | 0 … 7 (1.0) | 125 | 2.72 (2.11) | 0 … 7 (3.0) | **0.003** |
| 4 | 127 | 1.52 (1.80) | 0 … 6 (1.0) | 125 | 2.38 (2.09) | 0 … 7 (2.0) | **<0.001** |
| 5 | 127 | 1.23 (1.74) | 0 … 7 (0.0) | 125 | 1.97 (2.07) | 0 … 7 (1.0) | **0.001** |
| 6 | 127 | 1.03 (1.76) | 0 … 7 (0.0) | 125 | 1.54 (1.93) | 0 … 7 (1.0) | **0.003** |
| 7 | 127 | 0.74 (1.54) | 0 … 7 (0.0) | 125 | 1.16 (1.87) | 0 … 7 (0.0) | **0.011** |
| 8 | 127 | 0.52 (1.39) | 0 … 7 (0.0) | 125 | 0.82 (1.60) | 0 … 7 (0.0) | **0.047** |
| 9 | 127 | 0.33 (1.11) | 0 … 6 (0.0) | 125 | 0.68 (1.53) | 0 … 7 (0.0) | **0.008** |
| 10 | 127 | 0.32 (1.31) | 0 … 6 (0.0) | 125 | 0.50 (1.34) | 0 … 7 (0.0) | 0.052 |
| 11 | 127 | 0.31 (1.16) | 0 … 7 (0.0) | 125 | 0.38 (1.26) | 0 … 7 (0.0) | 0.408 |
| 12 | 127 | 0.14 (0.65) | 0 … 4 (0.0) | 125 | 0.26 (1.03) | 0 … 7 (0.0) | 0.143 |
| 13 | 127 | 0,08 (0.43) | 0 … 3 (0.0) | 125 | 0.23 (1.04) | 0 … 7 (0.0) | 0.274 |
| 14 | 127 | 0.06 (0.39) | 0 … 3 (0.0) | 125 | 0.19 (0.96) | 0 … 7 (0.0) | 0.247 |
| 15 | 127 | 0.04 (0.29) | 0 … 3 (0.0) | 125 | 0.18 (0.92) | 0 … 7 (0.0) | 0.191 |
| 16 | 127 | 0.02 (0.27) | 0 … 3 (0.0) | 125 | 0.16 (0.80) | 0 … 6 (0.0) | **0.043** |
| 17 | 127 | 0.02 (0.27) | 0 … 3 (0.0) | 125 | 0.09 (0.52) | 0 … 4 (0.0) | 0.089 |
| 18 | 127 | 0.02 (0.27) | 0 … 3 (0.0) | 125 | 0.02 (0.15) | 0 … 1 (0.0) | 0.368 |
| 19 | 127 | 0.00 |  | 125 | 0.02 (0.13) | 0 … 1 (0.0) | 0.245 |
| 20 | 127 | 0.00 |  | 125 | 0.01 (0.09) |  | 0.496 |

| **Item 16**  **Day** | **V group** | | | **P group** | | | **p_U_ – value** |
| --- | --- | --- | --- | --- | --- | --- | --- |
|  | N | Mean (SD) | Min … Max (Median) | N | Mean (SD) | Min … Max (Median) |  |
| 1 | 127 | 1.68 (1.74) | 0 … 6 (1.0) | 125 | 2.18 (1.96) | 0 … 7 (2.0) | 0.052 |
| 2 | 127 | 2.17 (1.96) | 0 … 7 (1.0) | 125 | 2.56 (2.00) | 0 … 7 (3.0) | 0.113 |
| 3 | 127 | 1.98 (1.95) | 0 … 7 (1.0) | 125 | 2.53 (2.04) | 0 … 7 (2.0) | **0.025** |
| 4 | 127 | 1.46 (1.83) | 0 … 6 (1.0) | 125 | 2.12 (1.98) | 0 … 7 (2.0) | **0.002** |
| 5 | 127 | 1.20 (1.81) | 0 … 7 (0.0) | 125 | 1.84 (2.07) | 0 … 7 (1.0) | **0.003** |
| 6 | 127 | 0.97 (1.76) | 0 … 7 (0.0) | 125 | 1.46 (1.88) | 0 … 7 (1.0) | **0.002** |
| 7 | 127 | 0.73 (1.59) | 0 … 7 (0.0) | 125 | 1.11 (1.87) | 0 … 7 (0.0) | **0.024** |
| 8 | 127 | 0.50 (1.40) | 0 … 7 (0.0) | 125 | 0.71 (1.54) | 0 … 7 (0.0) | 0.092 |
| 9 | 127 | 0.33 (1.12) | 0 … 6 (0.0) | 125 | 0.63 (1.51) | 0 … 7 (0.0) | **0.017** |
| 10 | 127 | 0.30 (1.11) | 0 … 6 (0.0) | 125 | 0.44 (1.28) | 0 … 7 (0.0) | **0.047** |
| 11 | 127 | 0.28 (1.06) | 0 … 6 (0.0) | 125 | 0.38 (1.27) | 0 … 7 (0.0) | 0.302 |
| 12 | 127 | 0.12 (0.60) | 0 … 4 (0.0) | 125 | 0.26 (1.04) | 0 … 7 (0.0) | 0.111 |
| 13 | 127 | 0.07 (0.42) | 0 … 3 (0.0) | 125 | 0.22 (1.01) | 0 … 7 (0.0) | 0.174 |
| 14 | 127 | 0.06 (0.38) | 0 … 3 (0.0) | 125 | 0.20 (0.96) | 0 … 7 (0.0) | 0.089 |
| 15 | 127 | 0.02 (0.27) | 0 … 3 (0.0) | 125 | 0.18 (0.92) | 0 … 7 (0.0) | **0.036** |
| 16 | 127 | 0.02 (0.27) | 0 … 3 (0.0) | 125 | 0.15 (0.77) | 0 … 6 (0.0) | **0.043** |
| 17 | 127 | 0.02 (0.27) | 0 … 3 (0.0) | 125 | 0.05 (0.38) | 0 … 4 (0.0) | 0.307 |
| 18 | 127 | 0.02 (0.27) | 0 … 3 (0.0) | 125 | 0.02 (0.13) | 0 … 1 (0.0) | 0.621 |
| 19 | 127 | 0.00 |  | 125 | 0.01 (0.09) | 0 … 1 (0.0) | 0.496 |
| 20 | 127 | 0.00 |  | 125 | 0.00 |  | 1.000 |

| **Item 17**  **Day** | **V group** | | | **P group** | | | **p_U_ – value** |
| --- | --- | --- | --- | --- | --- | --- | --- |
|  | N | Mean (SD) | Min … Max (Median) | N | Mean (SD) | Min … Max (Median) |  |
| 1 | 127 | 1.74 (1.89) | 0 … 6 (1.0) | 125 | 2.22 (2.06) | 0 … 7 (2.0) | 0.065 |
| 2 | 127 | 2.07 (2.00) | 0 … 7 (1.0) | 125 | 2.67 (2.11) | 0 … 7 (3.0) | **0.019** |
| 3 | 127 | 1.94 (2.03) | 0 … 7 (1.0) | 125 | 2.54 (2.21) | 0 … 7 (2.0) | **0.027** |
| 4 | 127 | 1.46 (1.85) | 0 … 6 (1.0) | 125 | 2 22 (2.17) | 0 … 7 (2.0) | **0.002** |
| 5 | 127 | 1.21 (1.83) | 0 … 7 (0.0) | 125 | 1.91 (2.11) | 0 … 7 (1.0) | **0.001** |
| 6 | 127 | 0.95 (1.77) | 0 … 7 (0.0) | 125 | 1.55 (2.05) | 0 … 7 (1.0) | **0.001** |
| 7 | 127 | 0.72 (1.57) | 0 … 7 (0.0) | 125 | 1.21 (1.91) | 0 … 7 (0.0) | **0.009** |
| 8 | 127 | 0.49 (1.38) | 0 … 7 (0.0) | 125 | 0.76 (1.57) | 0 … 7 (0.0) | **0.034** |
| 9 | 127 | 0.32 (1.10) | 0 … 6 (0.0) | 125 | 0.64 (1.52) | 0 … 7 (0.0) | **0.016** |
| 10 | 127 | 0.28 (1.04) | 0 … 6 (0.0) | 125 | 0.47 (1.34) | 0 … 7 (0.0) | **0.029** |
| 11 | 127 | 0.30 (1.16) | 0 … 7 (0.0) | 125 | 0.37 (1.25) | 0 … 7 (0.0) | 0.316 |
| 12 | 127 | 0.11 (0.58) | 0 … 4 (0.0) | 125 | 0.25 (1.04) | 0 … 7 (0.0) | 0.200 |
| 13 | 127 | 0.06 (0.39) | 0 … 3 (0.0) | 125 | 0.22 (1.02) | 0 … 7 (0.0) | 0.155 |
| 14 | 127 | 0.06 (0.44) | 0 … 4 (0.0) | 125 | 0.20 (0.96) | 0 … 7 (0.0) | **0.044** |
| 15 | 127 | 0.04 (0.29) | 0 … 3 (0.0) | 125 | 0.17 (0.91) | 0 … 7 (0.0) | 0.297 |
| 16 | 127 | 0.02 (0.27) | 0 … 3 (0.0) | 125 | 0.14 (0.76) | 0 … 6 (0.0) | **0.043** |
| 17 | 127 | 0.02 (0.27) | 0 … 3 (0.0) | 125 | 0.06 (0.39) | 0 … 4 (0.0) | 0.181 |
| 18 | 127 | 0.02 (0.27) | 0 … 3 (0.0) | 125 | 0.01 (0.09) | 0 … 1 (0.0) | 1.000 |
| 19 | 127 | 0.00 |  | 125 | 0.01 (0.09) | 0 … 1 (0.0) | 0.496 |
| 20 | 127 | 0.00 |  | 125 | 0.00 |  | 1.000 |

| **Item 18**  **Day** | **V group** | | | **P group** | | | **p_U_ – value** |
| --- | --- | --- | --- | --- | --- | --- | --- |
|  | N | Mean (SD) | Min … Max (Median) | N | Mean (SD) | Min … Max (Median) |  |
| 1 | 127 | 1.61 (1.73) | 0 … 6 (1.0) | 125 | 2.04 (1.93) | 0 … 7 (2.0) | 0.083 |
| 2 | 127 | 1.97 (1.89) | 0 … 6 (1.0) | 125 | 2.54 (2.08) | 0 … 7 (3.0) | **0.032** |
| 3 | 127 | 1.83 (1.91) | 0 … 7 (1.0) | 125 | 2.39 (2.08) | 0 … 7 (2.0) | **0.032** |
| 4 | 127 | 1.33 (1.75) | 0 … 6 (0.0) | 125 | 2.06 (2.03) | 0 … 7 (1.0) | **0.002** |
| 5 | 127 | 1.09 (1.78) | 0 … 7 (0.0) | 125 | 1.74 (2.02) | 0 … 7 (1.0) | **0.002** |
| 6 | 127 | 0.89 (1.70) | 0 … 7 (0.0) | 125 | 1.37 (1.87) | 0 … 7 (1.0) | **0.002** |
| 7 | 127 | 0.68 (1.59) | 0 … 7 (0.0) | 125 | 1.07 (1.86) | 0 … 7 (0.0) | **0.019** |
| 8 | 127 | 0.44 (1.34) | 0 … 7 (0.0) | 125 | 0.67 (1.50) | 0 … 7 (0.0) | 0.054 |
| 9 | 127 | 0.32 (1.10) | 0 … 6 (0.0) | 125 | 0.59 (1.48) | 0 … 7 (0.0) | **0.016** |
| 10 | 127 | 0.27 (1.05) | 0 … 6 (0.0) | 125 | 0.41 (1.26) | 0 … 7 (0.0) | 0.058 |
| 11 | 127 | 0.26 (1.02) | 0 … 6 (0.0) | 125 | 0.37 (1.24) | 0 … 7 (0.0) | 0.225 |
| 12 | 127 | 0.10 (0.55) | 0 … 4 (0.0) | 125 | 0.25 (1.03) | 0 … 7 (0.0) | 0.143 |
| 13 | 127 | 0.07 (0.42) | 0 … 3 (0.0) | 125 | 0.22 (1.01) | 0 … 7 (0.0) | 0.174 |
| 14 | 127 | 0.06 (0.38) | 0 … 3 (0.0) | 125 | 0.19 (0.96) | 0 … 7 (0.0) | 0.138 |
| 15 | 127 | 0.05 (0.33) | 0 … 3 (0.0) | 125 | 0.17 (0.91) | 0 … 7 (0.0) | 0.305 |
| 16 | 127 | 0.02 (0.27) | 0 … 3 (0.0) | 125 | 0.14 (0.76) | 0 … 6 (0.0) | 0.074 |
| 17 | 127 | 0.02 (0.27) | 0 … 3 (0.0) | 125 | 0.05 (0.38) | 0 … 4 (0.0) | 0.307 |
| 18 | 127 | 0.02 (0.27) | 0 … 3 (0.0) | 125 | 0.00 |  | 1.000 |
| 19 | 127 | 0.00 |  | 125 | 0.00 |  | 1.000 |
| 20 | 127 | 0.00 |  | 125 | 0.00 |  | 1.000 |

| **Item 19**  **Day** | **V group** | | | **P group** | | | **p_U_ – value** |
| --- | --- | --- | --- | --- | --- | --- | --- |
|  | N | Mean (SD) | Min … Max (Median) | N | Mean (SD) | Min … Max (Median) |  |
| 1 | 127 | 1.57 (1.76) | 0 … 6 (1.0) | 125 | 2.14 (2.08) | 0 … 7 (2.0) | **0.050^4^** |
| 2 | 127 | 1.83 (1.94) | 0 … 7 (1.0) | 125 | 2.47 (2.14) | 0 … 7 (3.0) | **0.014** |
| 3 | 127 | 1.77 (1.92) | 0 … 7 (1.0) | 125 | 2.44 (2.20) | 0 … 7 (2.0) | **0.014** |
| 4 | 127 | 1.31 (1.75) | 0 … 7 (0.0) | 125 | 2.02 (2.03) | 0 … 7 (1.0) | **0.002** |
| 5 | 127 | 1.04 (1.60) | 0 … 7 (0.0) | 125 | 1.70 (2.02) | 0 … 7 (1.0) | **0.005** |
| 6 | 127 | 0.87 (1.71) | 0 … 7 (0.0) | 125 | 1.30 (1.90) | 0 … 7 (0.0) | **0.006** |
| 7 | 127 | 0.69 (1.59) | 0 … 7 (0.0) | 125 | 0.98 (1.81) | 0 … 7 (0.0) | 0.133 |
| 8 | 127 | 0.50 (1.39) | 0 … 7 (0.0) | 125 | 0.65 (1.44) | 0 … 7 (0.0) | 0.157 |
| 9 | 127 | 0.33 (1.12) | 0 … 6 (0.0) | 125 | 0.57 (1.43) | 0 … 7 (0.0) | 0.054 |
| 10 | 127 | 0.28 (1.06) | 0 … 6 (0.0) | 125 | 0.42 (1.28) | 0 … 7 (0.0) | 0.082 |
| 11 | 127 | 0.28 (1.10) | 0 … 6 (0.0) | 125 | 0.34 (1.20) | 0 … 7 (0.0) | 0.320 |
| 12 | 127 | 0.12 (0.60) | 0 … 4 (0.0) | 125 | 0.25 (1.03) | 0 … 7 (0.0) | 0.159 |
| 13 | 127 | 0.06 (0.39) | 0 … 3 (0.0) | 125 | 0.22 (1.02) | 0 … 7 (0.0) | 0.155 |
| 14 | 127 | 0.06 (0.45) | 0 … 4 (0.0) | 125 | 0.20 (0.96) | 0 … 7 (0.0) | 0.092 |
| 15 | 127 | 0.04 (0.29) | 0 … 3 (0.0) | 125 | 0.14 (0.85) | 0 … 7 (0.0) | 0.320 |
| 16 | 127 | 0.02 (0.27) | 0 … 3 (0.0) | 125 | 0.16 (0.86) | 0 … 6 (0.0) | 0.059 |
| 17 | 127 | 0.02 (0.27) | 0 … 3 (0.0) | 125 | 0.05 (0.38) | 0 … 4 (0.0) | 0.307 |
| 18 | 127 | 0.02 (0.27) | 0 … 3 (0.0) | 125 | 0.02 (0.27) | 0 … 3 (0.0) | 1.000 |
| 19 | 127 | 0.00 |  | 125 | 0.01 (0.09) | 0 … 1 (0.0) | 0.496 |
| 20 | 127 | 0.00 |  | 125 | 0.00 |  | 1.000 |

| **Item 20**  **Day** | **V group** | | | **P group** | | | **p_U_ – value** |
| --- | --- | --- | --- | --- | --- | --- | --- |
|  | N | Mean (SD) | Min … Max (Median) | N | Mean (SD) | Min … Max (Median) |  |
| 1 | 127 | 1.50 (1.61) | 0 … 6 (1.0) | 125 | 2.00 (2.02) | 0 … 7 (1.0) | 0.101 |
| 2 | 127 | 1.85 (1.87) | 0 … 7 (1.0) | 125 | 2.34 (2.10) | 0 … 7 (2.0) | 0.073 |
| 3 | 127 | 1.68 (1.89) | 0 … 6 (1.0) | 125 | 2.30 (2.10) | 0 … 7 (2.0) | **0.013** |
| 4 | 127 | 1.28 (1.73) | 0 … 6 (0.0) | 125 | 1.90 (1.98) | 0 … 7 (1.0) | **0.003** |
| 5 | 127 | 1.01 (1.70) | 0 … 7 (0.0) | 125 | 1.62 (2.00) | 0 … 7 (1.0) | **0.002** |
| 6 | 127 | 0.87 (1.75) | 0 … 7 (0.0) | 125 | 1.23 (1.90) | 0 … 7 (0.0) | **0.019** |
| 7 | 127 | 0.64 (1,52) | 0 … 7 (0.0) | 125 | 0.90 (1.79) | 0 … 7 (0.0) | 0.206 |
| 8 | 127 | 0.46 (1.36) | 0 … 7 (0.0) | 125 | 0.63 (1.52) | 0 … 7 (0.0) | 0.181 |
| 9 | 127 | 0.32 (1.10) | 0 … 6 (0.0) | 125 | 0.56 (1.48) | 0 … 7 (0.0) | **0.049** |
| 10 | 127 | 0.28 (1.06) | 0 … 6 (0.0) | 125 | 0.39 (1.29) | 0 … 7 (0.0) | 0.160 |
| 11 | 127 | 0.28 (1.10) | 0 … 6 (0.0) | 125 | 0.32 (1.18) | 0 … 7 (0.0) | 0.406 |
| 12 | 127 | 0.13 (0.60) | 0 … 4 (0.0) | 125 | 0.23 (1.02) | 0 … 7 (0.0) | 0.337 |
| 13 | 127 | 0.08 (0.46) | 0 … 3 (0.0) | 125 | 0.22 (1.01) | 0 … 7 (0.0) | 0.178 |
| 14 | 127 | 0.06 (0.38) | 0 … 3 (0.0) | 125 | 0.19 (0.96) | 0 … 7 (0.0) | 0.138 |
| 15 | 127 | 0.04 (0.29) | 0 … 3 (0.0) | 125 | 0.15 (0.85) | 0 … 7 (0.0) | 0.210 |
| 16 | 127 | 0.02 (0.27) | 0 … 3 (0.0) | 125 | 0.11 (0.73) | 0 … 6 (0.0) | 0.184 |
| 17 | 127 | 0.02 (0.27) | 0 … 3 (0.0) | 125 | 0.04 (0.37) | 0 … 4 (0.0) | 0.496 |
| 18 | 127 | 0.02 (0.27) | 0 … 3 (0.0) | 125 | 0.00 |  | 1.000 |
| 19 | 127 | 0.00 |  | 125 | 0.00 |  | 1.000 |
| 20 | 127 | 0.00 |  | 125 | 0.00 |  | 1.000 |

**SC: Comparison between Jackson score and 9-item WURSS-21 QoL domain**

Reference: Table 126 and 186 in CI30-018

| **Day** | **V group (N = 136)** | **P group (N = 131)** | **Difference (CI)** | **p_U_ value** |
| --- | --- | --- | --- | --- |
|  | Mean (SD) | Mean (SD) |  |  |
| 1 | 8.7 (4.4) | 9.0 (5.0) | 0.2 (-1.0 … 1.4) | 0.886 |
| 2 | 18.2 (8.5) | 19.3 (9.4) | 1.1 (-1.1 … 3.3) | 0.372 |
| 3 | 26.1 (12.4) | 28.6 (13.9) | 1.6 (-0.8 … 5.7) | 0.171 |
| 4 | 32.2 (16.5) | 36.4 (18.0) | 4.2 (0.0 … 8.4) | **0.044** |
| 5 | 36.5 (19.9) | 42.3 (21.8) | 5.8 (0.8 … 10.9) | **0.022** |
| 6 | 39.5 (22.7) | 46.3 (25.0) | 6.8 (1.0 … 12.6) | **0.021** |
| 7 | 41.9 (25.4) | 49.2 (27.9) | 7.3 (0.8 … 13.7) | **0.026** |

| **Sum Score of WURSS-21**  **AUC (7 days)** | **N** | **Mean** | **SD** | **Min** | **Q25** | **Median** | **Q75** | **Max** | **CI** |
| --- | --- | --- | --- | --- | --- | --- | --- | --- | --- |
| total | 243 | 107.5 | 81.8 | 0.0 | 40.5 | 93.0 | 159.8 | 333.1 | 97.1 … 117.9 |
| V group | 121 | 91.5 | 74.1 | 0.0 | 33.0 | 80.0 | 125.0 | 281.5 | 78.1 … 104.9 |
| P group | 122 | 123.3 | 86.1 | 0.0 | 50.0 | 108.0 | 178.5 | 333.1 | 107.8 … 138.8 |
| p_U_ value | | 0.003 | | | | | | |  |
